# Impact of concomitant medications on the efficacy of immune checkpoint inhibitors: an umbrella review

**DOI:** 10.3389/fimmu.2023.1218386

**Published:** 2023-09-29

**Authors:** Honglin Li, Lei Zhang, Feiran Yang, Ruohan Zhao, Xiurong Li, Huijie Li

**Affiliations:** ^1^ First Clinical College, Shandong University of Traditional Chinese Medicine, Jinan, Shandong, China; ^2^ Department of Oncology, Affiliated Hospital of Shandong University of Traditional Chinese Medicine, Jinan, Shandong, China; ^3^ Department of Oncology, Longhua Hospital Affiliated to Shanghai University of Traditional Chinese Medicine, Shanghai, China

**Keywords:** concomitant medications, immune checkpoint inhibitors, efficacy, umbrella review, meta-analysis

## Abstract

**Introduction:**

Cancer is a major global health concern, and immune checkpoint inhibitors (ICIs) offer a promising treatment option for cancer patients. However, the efficacy of ICIs can be influenced by various factors, including the use of concomitant medications.

**Methods:**

We searched databases (PubMed, Embase, Cochrane Library, Web of Science) for systematic reviews and meta-analyses for systematic reviews and meta-analyses on the impact of concomitant medications on ICIs efficacy, published from inception to January 1, 2023. We evaluated the methodological quality of the included meta-analyses, and re-synthesized data using a random-effects model and evidence stratification.

**Results:**

We included 23 publications, comprising 11 concomitant medications and 112 associations. Class II-IV evidence suggested that antibiotics have a negative impact on ICIs efficacy. However, ICIs efficacy against melanoma, hepatocellular carcinoma, and esophageal squamous cell carcinoma was not affected, this effect was related to the exposure window (class IV). Class III evidence suggested that proton pump inhibitors have a negative impact on ICIs efficacy; nevertheless, the efficacy against melanoma and renal cell carcinoma was not affected, and the effect was related to exposure before the initiation of ICIs therapy (class II). Although class II/III evidence suggested that steroids have a negative impact, this effect was not observed when used for non-cancer indications and immune-related adverse events (class IV). Class IV evidence suggested that opioids reduce ICIs efficacy, whereas statins and probiotics may improve ICIs efficacy. ICIs efficacy was not affected by histamine 2 receptor antagonists, aspirin, metformin, β-blockers, and nonsteroidal anti-inflammatory agents.

**Conclusion:**

Current evidence suggests that the use of antibiotics, PPIs, steroids, and opioids has a negative impact on the efficacy of ICIs. However, this effect may vary depending on the type of tumor, the timing of exposure, and the intended application. Weak evidence suggests that statins and probiotics may enhance the efficacy of ICIs. Aspirin, metformin, β-blockers, and NSAIDs do not appear to affect the efficacy of ICIs. However, caution is advised in interpreting these results due to methodological limitations.

**Systematic review registration:**

https://www.crd.york.ac.uk/PROSPERO,identifier, CRD42022328681.

## 1 Introduction

Cancer poses a major threat to human health, with increasing incidence and mortality rates imposing a heavy burden on societies worldwide (1). Immune checkpoint inhibitors (ICIs) have emerged as an important treatment option, bringing new hope to patients (2). However, the efficacy of ICIs is influenced by various factors (3), including the use of concomitant medications (4).

Antibiotics, proton pump inhibitors (PPIs), and steroids are widely used in clinical practice. Multiple clinical studies and meta-analyses have suggested that these concomitant medications may significantly reduce the efficacy of ICIs (5, 6). Nevertheless, other studies did not demonstrate this negative impact (7). Two almost simultaneously published meta-analyses investigating the effect of PPIs on ICIs efficacy yielded inconsistent results (8, 9). Additionally, a meta-analysis showed that the use of antibiotics during treatment with ICIs does not shorten progression-free survival (PFS), and may even prolong it (10). Moreover, the negative effect of antibiotics may also be related to the time window of use and the type of tumor (11), thereby complicating clinical decision-making.

Notably, besides the negative impact on ICIs efficacy, some concomitant medications (e.g., statins, metformin, β-blockers, and probiotics) may exert an enhancing effect on ICIs efficacy; however, the level of evidence remains poor and controversial (12). In recent years, the number of meta-analyses focusing on the impact of concomitant medications has surged. However, the quality of these analyses is uneven, and the results are inconsistent. Moreover, most meta-analyses only analyzed and evaluated a single type of concomitant medications, thus lacking comprehensiveness and systematicity. Therefore, it is necessary to comprehensively review and summarize published systematic reviews and meta-analyses, evaluate publication bias and evidence quality, explore the effects of different concomitant medications on ICIs efficacy, and present an overview of the available evidence for clinical application.

The objectives of this review were to: 1) comprehensively analyze and summarize the existing systematic reviews and meta-analyses; 2) use a random-effects model to re-synthesize the data; 3) evaluate publication bias and evidence quality; and 4) present an overview of the available evidence regarding the impact of concomitant medications on ICIs efficacy.

## 2 Methods

The protocol for this study has been submitted to and registered in PROSPERO (Registration number: CRD42022328681). This umbrella review was conducted in accordance with the PRISMA statement (13).

### 2.1 Search strategy

We used a pre-designed strategy to conduct a comprehensive and systematic search in the PubMed, Embase, Cochrane Library, and Web of Science databases from inception to January 1, 2023. The search was limited to articles published in English. In brief, the search terms included “concomitant medications”, “ICIs”, “systematic reviews”, and “meta-analyses”. Detailed search strategies and the results obtained from PubMed are presented in **Table S1**.

### 2.2 Inclusion and exclusion criteria

The detailed inclusion criteria were as follows: (1) study design was a systematic review or meta-analysis; (2) study population included patients with cancer receiving ICIs, with the observation and control groups receiving concomitant medications and no concomitant medications, respectively; (3) concomitant medications included antibiotics, steroids, PPIs, anticoagulants, lipid-lowering agents, antihypertensive agents, antidiabetic agents, probiotics, and analgesics; and (4) the study reported at least one outcome measure, namely overall survival (OS), PFS, objective response rate (ORR), progressive disease (PD), stable disease, complete response, partial response, and disease control rate.

Publications were excluded based on the following criteria: (1) animal experiments; (2) individual case reports; (3) network meta-analyses; (4) original clinical trials; (5) case reports; and (6) articles not published in English.

### 2.3 Literature screening and data extraction

Two reviewers (HLL and LZ) independently conducted literature screening based on the inclusion and exclusion criteria. After reading the title and abstract, full-text manuscripts were obtained for further evaluation. Any discrepancies were resolved through discussion with a third reviewer (HJL), reaching a consensus.

One reviewer (HLL) performed data extraction, and another reviewer (LZ) checked the data. The extracted data included the first author, year of publication, number of included studies, number of included patients, age, sex distribution, tumor type, co-medications type, follow-up duration, outcome measures, pooled effect size and 95% confidence interval (CI), quality assessment tool, conflict of interest, and funding information. For original studies included in the systematic review and meta-analysis, the extracted information included the first author, year of publication, sample size, effect size, and 95% CI. In addition, we also screened the reference lists of included studies to ensure that all available publications were included in the analysis.

### 2.4 Quality assessment

Two reviewers (HLL, LZ) independently conducted methodological quality assessments using Assessment of Multiple Systematic Reviews (AMSTAR) 2 (14), which evaluates 16 items sequentially (**Table S2**). Items 2, 4, 7, 9, 11, 13, and 15 are the critical domains. High quality is attributed to instances with no or just one non-critical weakness. Moderate quality applies to scenarios with multiple non-critical defects. Instances with one critical flaw, with or without non-critical weaknesses, are deemed of low quality. Critically low quality is assigned to situations featuring more than one critical flaw, with or without non-critical weaknesses. Any discrepancies were resolved through discussion with a third reviewer (HJL), reaching a consensus.

### 2.5 Removal of overlapping meta-analyses

In recent years, numerous meta-analyses on this topic have emerged. However, overlapping original studies for the same outcome measures may lead to bias. Therefore, we addressed this issue in our analysis. As in previously published umbrella reviews (15), we used the citation matrix and corrected covered area (CCA) to calculate the degree of overlap (16). If the CCA was >15%, we retained the most recent publication with the highest number of included studies and level of methodological quality. If the CCA was <15%, we retained both; however, in case of complete overlap, we retained the publication with the highest number of included studies.

### 2.6 Evidence grading

As previously described in an umbrella review (17), we categorized the evidence into five classes which are described below. Convincing evidence (class I) was characterized by a significant combined effect size (*p* < 10^−6^), significant effect size in the largest study (*p* < 0.05), low heterogeneity (I^2^ < 50%), 95% prediction interval (PI) that did not include the null value, no evidence of significant publication bias (*p* > 0.1) as indicated by Egger’s regression test, and >1,000 patients included in the meta-analysis. Highly suggestive evidence (class II) was characterized by a significant combined effect size (*p* < 10^−6^), significant effect size in the largest study (*p* < 0.05), and >1,000 patients included in the meta-analysis, but did not meet class I criteria. Suggestive evidence (class III) was characterized by a significant combined effect size (*p* < 10^−3^) and >1,000 patients included in the meta-analysis, but did not meet class I or II criteria. Weak evidence (class IV) was characterized by a significant combined effect size (*p* < 0.05), but did not meet class I–III criteria. Non-significant evidence (class ns) was characterized by no significant combined effect size (*p* > 0.05).

### 2.7 Statistical analysis

The DerSimonian–Laird (DL) method can underestimate the 95% CI when the number of included studies is small (18, 19). Therefore, we used the Hartung–Knapp–Sidik–Jonkman (HKSJ) method to analyze meta-data for fewer than five individual studies, and the DL method for more than five studies (20). Heterogeneity was assessed by calculating the I^2^ and 95% PI. An I^2^ >50% indicated significant heterogeneity, and the 95% PI predicted the potential range of true effects in the future.

For the assessment of publication bias, we first conducted Egger’s regression test. Subsequently, we evaluated all meta-analyses using contour-enhanced funnel plots. As in prior umbrella reviews (17, 21, 22), we assumed that small-study effects were present when Egger’s test yielded *p*-values <0.1. Therefore, we used the “trim-and-fill” method to re-estimate the effect size and 95% CI, thereby mitigating the potential impact of publication bias on the true results.

Furthermore, we conducted a test of excess significance to evaluate whether the number of observations of statistically significant results is greater than its expected number. In this test, *p*-values <0.1 indicated statistical significance (22). Additionally, we performed sensitivity analyses on meta-analyses with evidence grades I–III. This was achieved by excluding individual original studies with total sample sizes <100, re-synthesizing the data, and re-grading the evidence to test the robustness of the results and mitigate the bias introduced by studies with small samples. All data analyses were conducted using R software (version 4.1.1) using the ‘metaumbrella’ R package (23, 24), and *p*-values were two-tailed.

## 3 Results

### 3.1 Literature selection

**Figure 1:**
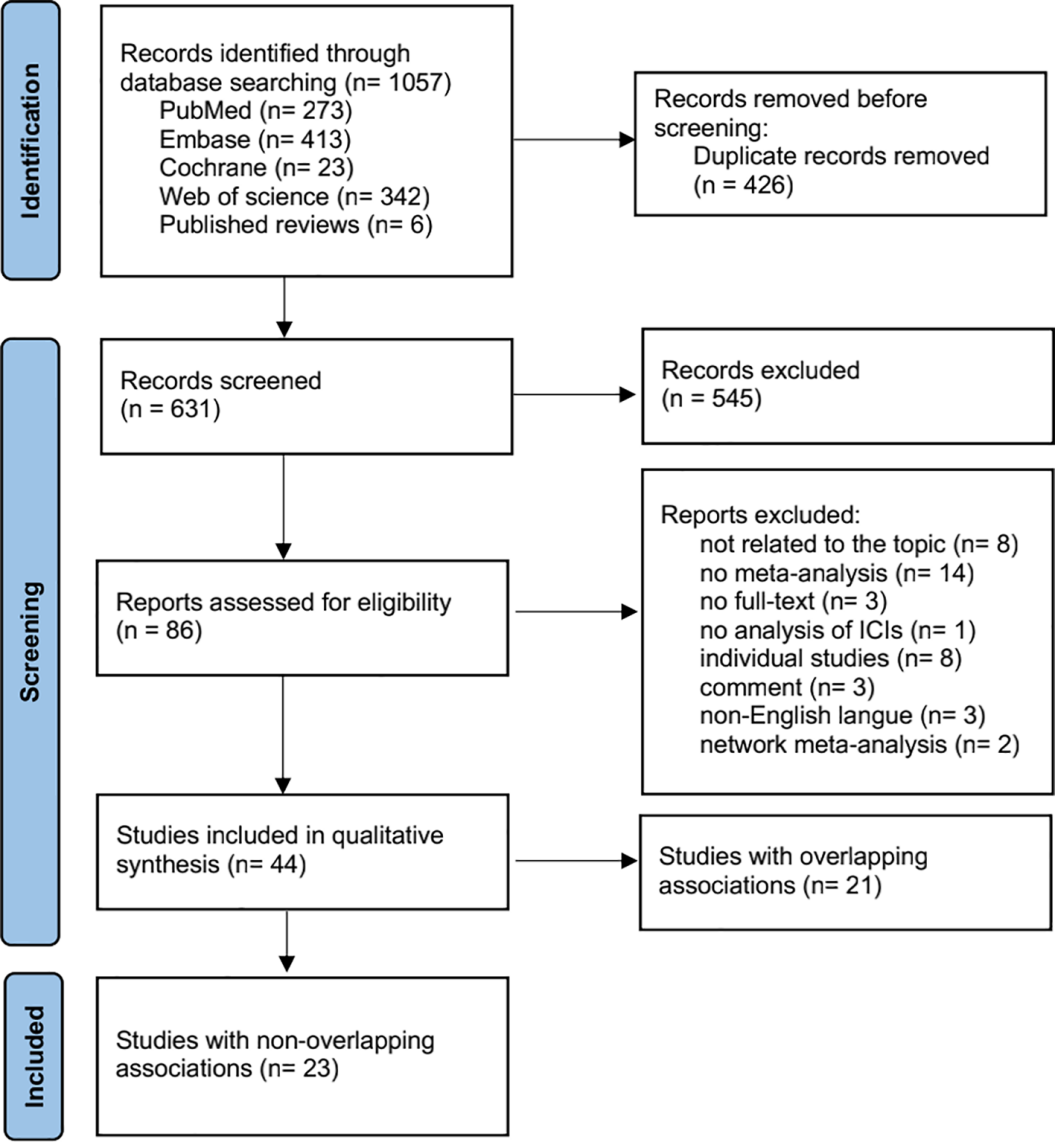
Flow chart of literature screening. ICIs, immune checkpoint inhibitors.

As shown in **Figure 1**, we obtained a total of 1,057 publications from four electronic databases and reference lists. After removing duplicates, 631 publications were selected for preliminary screening based on their titles and abstracts. Subsequently, 545 and 42 publications were excluded by the two reviewers after title/abstract and full-text reading, respectively. **Table S3** lists the excluded publications and exclusion criteria. We further removed overlapping studies by calculating the CCA (**Table S4**), resulting in a final set of 23 publications.

### 3.2 Basic characteristics

**Table 1** summarizes the basic characteristics of the 23 included publications (10–12, 25–44). These publications were from China (n=19), the United States of America (n=2), Australia (n=1), and France (n=1).

**Table 1:** Basic characteristics of included meta-analyses.

PMID	Author (year)	CM	Protocol	NO. of studies	Cancer type	Subgroup analysis	NO. of cases(CM+/CM-)	Outcome	Age (year)	NO. of male	Funding	Quality appraisal tool	Reporting guidelines	AMSTAR 2
35860836	Chen 2022 (25)	PPIs	CRD42020181618, INPLASY2020100088	33	Multiple cancer	Exposure window; ICIs types; cancer types	7383/8574	OS, PFS	NA	NA	Y	NOS	PRISMA	Critically low
36225582	Zhang 2022 (26)	PPIs	CRD42022332633	6	UC	NA	759/1221	OS, PFS	Median 67-72	1523	Y	NOS	PRISMA	Critically low
36301729	Deng 2022 (27)	PPIs/H2RAs	CRD42022329373	30	Multiple cancer	ICIs types; cancer types	6928/9219	OS, PFS, ORR	Median 57-70	NA	NA	NOS	MOOSE	Low
34323149	Chen 2021 (28)	ATB	NA	14	NSCLC	Exposure window; ICIs types; cancer types	430/1221^#^	OS, PFS	NA	NA	Y	NA	PRISMA	Critically low
NA	Crespin 2021 (29)	ATB	NA	35	NSCLC	Exposure window	2733/9512^#^	OS, PFS, ORR	NA	NA	NA	NA	NA	Critically low
33549013	Huang 2020 (10)	ATB	CRD42020155823	30	Multiple cancer	Exposure window	2129/6861^#^	PFS, OS	NA	NA	Y	NA	NA	Critically low
32173463	Lurienne 2020 (30)	ATB	CRD42019145675	23	NSCLC	Exposure window	1275/4469	PFS, OS	Media 62-75	NA	NA	NOS	PRISMA	Low
33771672	Tsikala-Vafea 2021 (31)	ATB	CRD42020166473	41	Multiple cancer	Exposure window; cancer types	2226/7391^#^	PFS, OS, response rate, disease progression	NA	NA	NA	NOS	PRISMA	Critically low
31865400	Wilson 2020 (32)	ATB	NA	18	Multiple cancer	Exposure window	826/2063	PFS, OS	Media 52-68	1270	NA	NA	NA	Critically low
33465728	Wu 2021 (11)	ATB	NA	44	Multiple cancer	Exposure window; ICIs types; cancer types	2929/9563	OS, PFS, ORR	Media 52-69	NA	NA	NA	NA	Critically low
36505435	Luo 2022 (33)	ATB	CRD42022349577	6	RCC	NA	242/862	PFS, OS, ORR	Media 61-63	NA	Y	NOS	PRISMA	Critically low
36059512	Zhang 2022 (34)	ATB	CRD42022311948	6	HCC	NA	352/723	PFS, OS, ORR, DCR	NA	NA	Y	NOS	PRISMA	Critically low
35967438	Zhou 2022 (35)	ATB	CRD42022330156	45	Multiple cancer	Exposure window; ICIs types	2298/6761^#^	OS, PFS	NA	NA	Y	NOS	NA	Critically low
33631792	Jessurun 2021 (36)	Steroids	NA	15	Multiple cancer	NA	354/736^#^	OS, PFS	Media 54-66	538	NA	NOS	PRISMA	Critically low
34358857	Wang 2021 (37)	Steroids	NA	32	Multiple cancer	Clinical features; ICIs types; cancer types	1830/6505	OS, PFS	Media 52-74	NA	Y	NOS	PRISMA	Critically low
34138497	Zhang 2021 (38)	Steroids	NA	14	NSCLC	NA	1072/3934	OS, PFS	Media 63-70.5	NA	NA	NOS	PRISMA	Critically low
34377596	Zhang 2021 (39)	Metformin, Statin, Low-dose aspirin, NSAIDs, β-blockers	NA	13	Multiple cancer	CM types; cancer types;	782/2551^#^	OS, PFS	NA	NA	Y	NOS	NA	Critically low
36330916	Yan 2022 (40)	β-blockers	NA	11	Multiple cancer	NA	3418/6817	OS, PFS	Media 57.5-73.7	NA	Y	NOS	PRISMA	Critically low
35855055	Zhang 2022 (12)	Statins	INPLASY202250110	8	NSCLC	NA	513/1869	OS, PFS	Media 65-71	NA	NA	NOS	MOOSE	Critically low
36110546	Zhang 2022 (41)	Probiotics	CRD42022316104	6	Multiple cancer	Cancer types	159/964	OS, PFS, ORR, DCR	NA	806	NA	NOS	PRISMA	Critically low
35770869	Wan 2022 (42)	Probiotics	NA	5	NSCLC	NA	103/928	OS, PFS, ORR	NA	NA	NA	NOS	PRISMA	Critically low
35603146	Mao 2022 (43)	Analgesics (opioids, NSAIDs)	CRD42021288940	11	Multiple cancer	NA	1004/3400	OS, PFS, ORR	NA	NA	NA	NOS	PRISMA	Critically low
36538147	Ju 2022 (44)	Opioids	NA	7	Multiple cancer	NA	620/2070	OS, PFS	Media 64-70	1817	NA	NOS	PRISMA	Critically low

^#^ Review reported incomplete data on sample size.

AMSTAR, assessment of multiple systematic reviews; ATB, antibiotics; CM, concomitant medication; DCR disease control rate; H2RAs, H2 receptor antagonists; HR, hazard ratio; ICIs, immune check inhibitors; NA, Not Available; MOOSE, meta-analyses of observational studies in epidemiology; NO., number; NOS, the Newcastle-Ottawa scale; NSAIDs, nonsteroidal anti-inflammatory drugs; NSCLC, non-small cell lung cancer; OS, overall survival; PFS, progression-free survival; PPIs, proton pump inhibitors; PRISMA, preferred reporting items for systematic reviews and meta-analyses; ORR, objective response rate; OR, odds ratio; RRC, renal cell carcinoma; UC, urothelial carcinoma; Y, Yes.

The 112 associations of antibiotics (n = 40), PPIs (n = 23), histamine 2 receptor antagonists (H2RAs; n = 3), steroids (n = 21), statins (n = 4), aspirin (n = 2), metformin (n = 2), β-blockers (n = 2), probiotics (n = 9), nonsteroidal anti-inflammatory agents (NSAIDs; n = 3) and opioids (n=3) are presented in **Table S5**. The number of original studies included ranged 5–45; the median age of the patients ranged 52–75 years. In addition, we compiled a list of 110 associations (antibiotics [n =69], PPIs [n = 28], steroids [n = 2], β-blockers [n =4], NSAIDs [n = 2], opioids [n = 2], and probiotics [n = 3]) that were excluded due to overlap.

### 3.3 Quality assessment

**Figure S1** shows the methodological quality assessment of the final 23 included publications and the 21 excluded publications due to overlap. Two articles on PPIs/H2RAs and one article on antibiotics were identified as low-quality publications due to the lack of a list of excluded literature. The remaining 41 publications were deemed to be of critically low quality, 27 (66%) were not registered with a protocol before conducting the meta-analysis, 35 (85%) did not discuss the sources of bias risk in detail, and 11 (27%) did not include tests and analyses on publication bias.

### 3.4 Overlapping associations

In the overlapping associations, 26 (24%) yielded inconsistent results with the included data. Regarding exposure to PPIs, 13 associations yielded inconsistent findings. Of note, two meta-analyses showed convincing evidence (class I) that PPIs reduced OS and PFS in any exposure window. Nevertheless, they were excluded due to the small number of included studies and overlapping. Regarding exposure to antibiotics, 13 associations yielded inconsistent results, mainly focusing on the initiation of ICIs therapy and RCC. Further details are provided in **Table S6** and **Figures S2**, **S3**, **S4**.

### 3.5 Antibiotics

**Figure 2:**
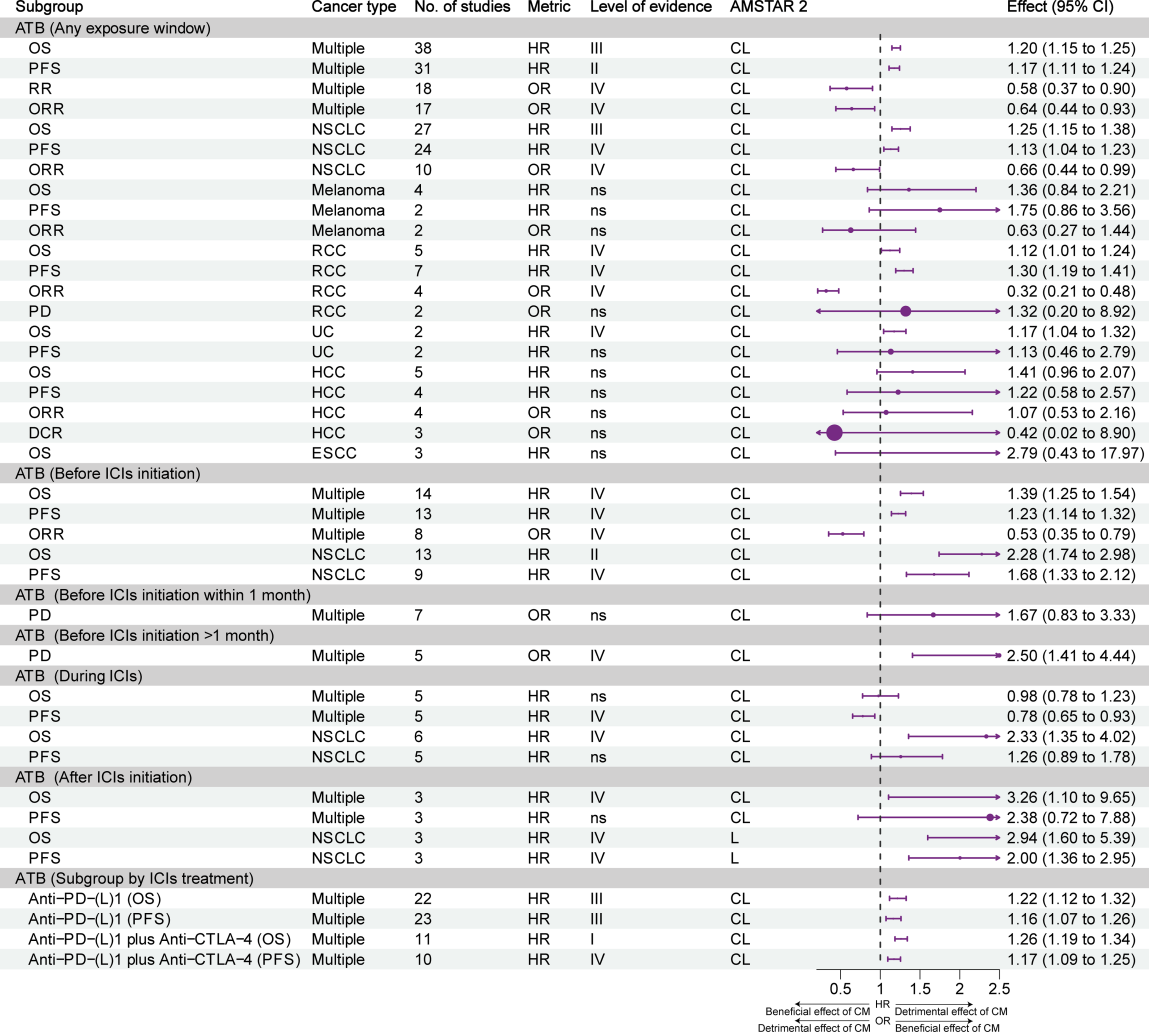
Forest plot of the prognosis of patients with cancer patients receiving ICIs and antibiotics, and subgroup analysis by cancer type and exposure window. ATB, antibiotic; CI, confidence interval; CL, critically low; DCR, disease control rate; ESCC, esophageal squamous cell carcinoma; HCC, hepatocellular carcinoma; HR, hazard ratio; ICIs, immune checkpoint inhibitors; L, low; NSCLC, non-small cell lung cancer; OR, odds ratio; ORR, objective response rate; OS, overall survival; PD, progressive disease; PFS, progression-free survival; RCC, renal cell carcinoma; UC, urothelial carcinoma; CM, concomitant medications.

**Figure 2** shows the effects of different exposure windows to antibiotics on the prognosis of different types of cancer and the level of evidence. Overall, based on all exposure windows and without distinguishing cancer types, suggestive evidence (class III) indicated that exposure to antibiotics reduced the OS of patients; highly suggestive evidence (class II) indicated reduced PFS; and weak evidence (class IV) showed reduced ORR and response rate. For non-small cell lung cancer (NSCLC), suggestive evidence (class III) showed that antibiotics reduced OS, and weak evidence (class IV) showed reduced PFS and ORR. For renal cell carcinoma (RCC), weak evidence (class IV) showed that antibiotics reduced OS, PFS, and ORR, but did not affect PD. For urothelial carcinoma (UC), weak evidence (class IV) showed that antibiotics reduced OS, but did not affect PFS. The prognosis of melanoma, hepatocellular carcinoma (HCC), and esophageal squamous cell carcinoma was not affected by antibiotics.

The negative effects of exposure to antibiotics before the initiation of ICIs were evident. Without distinguishing tumor types, weak evidence (class IV) indicated that antibiotics reduced OS, PFS, and ORR. Highly suggestive evidence (class II) and weak evidence (class IV) showed that antibiotics reduced the OS and PFS of patients with NSCLC. Weak evidence (class IV) suggested that the use of antibiotics within 1 month before the initiation of ICIs promoted PD in patients with cancer, while the use of antibiotics >1 month prior to the initiation of ICIs did not affect PD.

Regarding exposure to antibiotics during ICIs therapy, weak evidence (class IV) showed that antibiotics prolonged the PFS of patients with cancer, but did not exert an effect on OS. When NSCLC was analyzed separately, weak evidence (class IV) showed that antibiotics shortened OS, but did not affect PFS.

Concerning exposure to antibiotics after the initiation of treatment with ICIs, weak evidence (class IV) showed that antibiotics reduced the OS of patients with cancer, but did not exert an effect on PFS. Weak evidence (class IV) showed that antibiotics reduced the OS and PFS of patients with NSCLC.

Subgroup analysis of ICIs treatment showed that antibiotics reduced OS and PFS, whether with PD-(L)1 inhibitors alone (suggestive evidence, class II) or in combination with CTLA-4 inhibitors (Convincing and weak evidence, class I and IV).

### 3.6 PPIs/H2RAs

**Figure 3:**
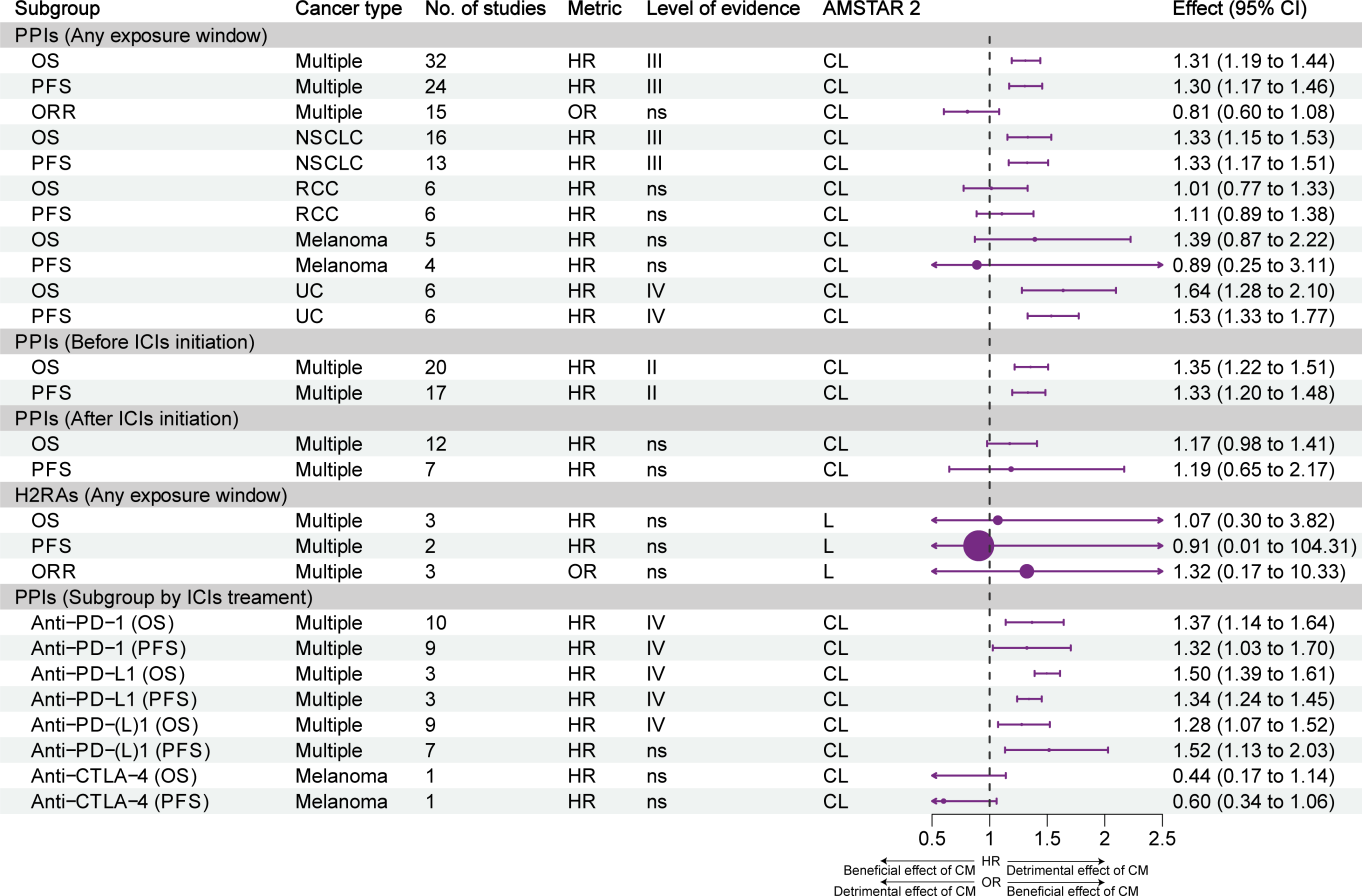
Forest plot of the prognosis of patients with cancer receiving ICIs and PPIs, and subgroup analysis by cancer type and exposure window. CI, confidence interval; CL, critically low; H2RAs, histamine 2 receptor antagonists; HR, hazard ratio; ICIs, immune checkpoint inhibitors; L, low; NSCLC, non-small cell lung cancer; OR, odds ratio; ORR, objective response rate; OS, overall survival; PFS, progression-free survival; PPI, proton pump inhibitor; RCC, renal cell carcinoma; UC, urothelial carcinoma; CM, concomitant medications.

**Figure 3** illustrates the effect of different exposure windows to PPIs/H2RAs on the prognosis of different types of tumors and the stratification of evidence. Overall, based on all exposure windows, suggestive evidence (class III) showed that the use of PPIs reduced OS and PFS in multiple types of cancer; however, it did not affect the ORR. Suggestive evidence (class III) showed that PPIs reduced OS and PFS in NSCLC and UC; nevertheless, the OS and PFS of patients with melanoma and RCC were not affected. Highly suggestive evidence (class II) showed that the OS and PFS of patients with cancer were reduced in the subgroup that used PPIs within 60 days before the initiation of ICIs therapy. However, these effects were not observed in the subgroup that used PPIs after the initiation of treatment with ICIs. Exposure to H2RAs did not affect the efficacy of ICIs. Subgroup analysis of ICIs treatment showed that PPIs reduced OS and PFS in combination with PD-1 or PD-L1 inhibitors alone (weak evidence, class IV), but not CTLA-4 inhibitors alone.

### 3.7 Steroids

**Figure 4:**
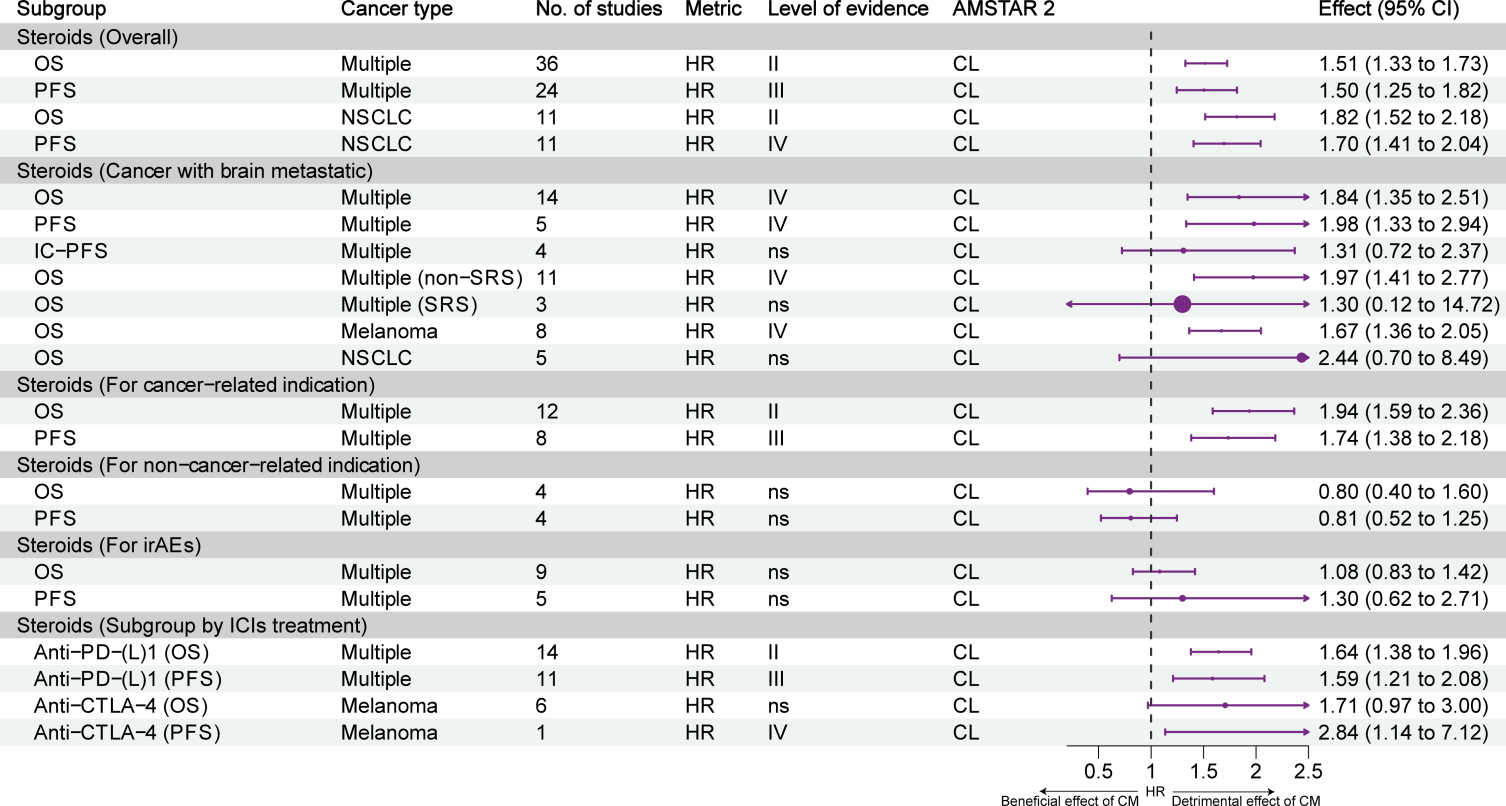
Forest plot of the prognosis of patients with cancer receiving ICIs and steroids, and subgroup analysis. CL, critically low; HR, hazard ratio; IC-PFS, intracranial progression-free survival; irAEs, immune-related adverse events; L, low; NSCLC, non-small cell lung cancer; OS, overall survival; PFS, progression-free survival; SRS, stereotactic radiosurgery; CM, concomitant medications.

**Figure 4** presents the effect of exposure to steroids on the prognosis of different types of tumors and the stratification of evidence. Overall, highly suggestive evidence (class II) and suggestive evidence (class III) showed that the use of steroids reduced OS and PFS in patients with cancer. Highly suggestive evidence (class II) and weak evidence (class IV) showed that steroids reduced the OS and PFS of patients with NSCLC. For cancer patients with brain metastasis, weak evidence (class IV) showed that steroids reduced OS and PFS, but not intracranial PFS. Moreover, steroids were associated with a reduction of OS in patients with brain metastasis who did not undergo stereotactic radiosurgery (SRS); however, the OS of patients who underwent SRS was not affected. Weak evidence (class IV) showed that steroids were associated with a reduction of OS in patients with melanoma but not in NSCLC patients with brain metastasis. Highly suggestive evidence (class II) and suggestive evidence (class III) showed that the use of steroids for cancer indications reduced the OS and PFS of patients. Nonetheless, the use of steroids for non-cancer indications and immune-related adverse events (irAEs) did not result in such effects. Subgroup analysis of ICIs treatment showed that steroids reduced OS and PFS in combination with PD-(L)1 inhibitors alone (highly suggestive and suggestive evidence, class II and III), reduced OS but not PFS in combination with CTLA-4 inhibitors alone (weak evidence, class IV).

### 3.8 Other concomitant medications

**Figure 5:**
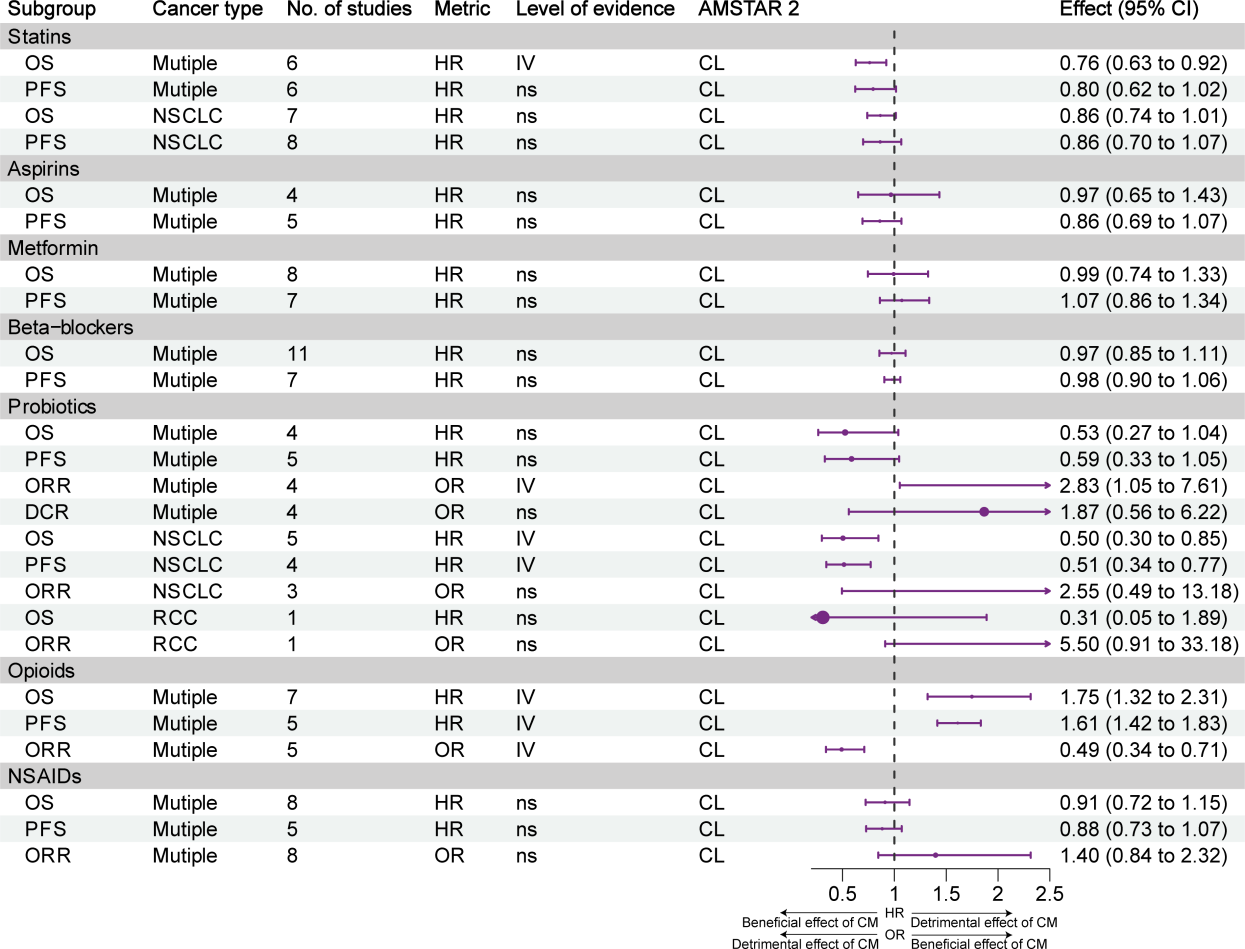
Forest plot of the prognosis of patients with cancer receiving ICIs and other concomitant medications (statins, aspirins, metformin, β-blockers, probiotics, opioids, NSAIDs), and subgroup analysis. CL, critically low; HR, hazard ratio; L, low; NSAIDs, nonsteroidal anti-inflammatory agents; NSCLC, non-small cell lung cancer; OR, odds ratio; OS, overall survival; PFS, progression-free survival; RCC, renal cell carcinoma; CM, concomitant medications. **Supplementary Figure** and table legends.

**Figure 5** illustrates the impact and evidence grading of exposure to statins, aspirin, metformin, β-blockers, probiotics, opioids, and NSAIDs on the prognosis of different types of tumors. Weak evidence (class IV) suggested that statin use was associated with prolonged OS, but not PFS, in patients with cancer. Although statin use was linked to a trend for improvement in OS and PFS in NSCLC, the effect was not statistically significant. Weak evidence (class IV) indicated that probiotics increased the ORR, OS, and PFS of patients with NSCLC; however, they did not have an impact on the OS and ORR of patients with RCC. Although the use of probiotics was associated with a trend for improvement in OS and PFS in multiple types of cancer, the effect was not statistically significant. Weak evidence (class IV) suggested that the use of opioids was associated with a decrease in OS, PFS, and ORR. The use of aspirin, metformin, β-blockers, and NSAIDs did not have an impact on the efficacy of ICIs.

### 3.9 Publication bias

**Figures S5**, **S6**, **S7**, **S9**, **S10** show an enhanced funnel plot of the meta-analyses that included more than three original studies. According to the results of the Egger’s regression test, 24 associations were characterized by a small sample effect (**Table S7**). The subsequent analysis using the ‘trim-and-fill’ method showed that the results of four associations lost statistical significance after adding studies. These included the effect of exposure to PPIs on OS in NSCLC, the effect of exposure to antibiotics on OS in multiple cancer types, the effect of exposure to antibiotics before the initiation of ICIs therapy on OS in NSCLC, and the effect of exposure to antibiotics on OS in RCC. The results of a associations gained statistical significance after adding studies (i.e., effect of exposure to PPIs on OS in melanoma.

### 3.10 Sensitivity analysis

**Table S8** shows the sensitivity analysis of associations with evidence grading of II–III by removing small sample studies. The evidence grading for the effect of exposure to PPIs in any exposure window on PFS decreased from class II to III; the evidence grading for the effect of exposure to PPIs on OS in NSCLC decreased from class III to IV; regarding the effect on PFS, the evidence grading increased from class III to II. The evidence grading for the effect of exposure to antibiotics in any exposure window on PFS decreased from class II to III.

## 4 Discussion

The efficacy of ICIs is influenced by numerous factors, including the tumor mutational burden, programmed death- ligand 1 (PD-L1) expression, and DNA mismatch repair gene defects (45). As a potentially controllable external factor, the interaction between ICIs and concomitant medications should be considered to ensure treatment effectiveness.

To our knowledge, this is the first umbrella review of the effects of 11 concomitant medications on the efficacy of ICIs. We used rigorous quality assessment and exclusion of overlapping studies, included a total of 23 published articles, re-conducted meta-analysis synthesis and assessment of the certainty of evidence, and combed the available findings. Overall, exposure to antibiotics, PPIs, steroids, and opioids was identified as the main factor leading to a decrease in the efficacy of ICIs. Statins and probiotics may exert positive effects, while treatment with H2RAs, metformin, β-blockers, aspirin, and NSAIDs did not affect the efficacy.

Analyses demonstrated that antibiotics have a significant impact on the efficacy of ICIs against NSCLC, RCC, and UC, but not against melanoma, HCC, and esophageal squamous cell carcinoma. Recent studies have shown that the use of antibiotics in patients with HCC early in the course of treatment with ICIs can improve prognosis, possibly by reducing the abundance of bacteria with immune inhibitory functions (46). Exposure to antibiotics before or after the initiation of ICIs is associated with a decline in prognosis, while the negative effects of exposure to antibiotics during ICIs use are more limited. The study conducted by Hogue et al. (47), which was included in the meta-analysis, played a critical role in the results, nevertheless, this is an abstract article that provides insufficient information and may have significant bias. We found that three associations had significant publication bias. The statistical significance of the results of these associations were lost after re-analysis using the trim and fill method, indicating that the negative effects of antibiotics on ICIs may be overestimated.

The mechanism by which antibiotics affect the efficacy of ICIs includes changes in the gut microbiota and inhibition of immune cell responses (48). A retrospective study showed that antibiotics had a negative impact on the prognosis of treatment with ICIs, but not chemotherapy. This, to some extent, ruled out the interference of potential adverse prognostic factors on the results (5). Different types of antibiotics may exert different negative effects, and studies have shown that quinolones, carbapenems, and cephalosporins do not decrease OS or PFS (49). Additionally, studies have demonstrated that the negative effects of antibiotics vary depending on the expression of PD-L1; patients with high PD-L1 expression were more susceptible to the effects of antibiotics (50). However, subgroup analysis based on PD-L1 expression was not included in our meta-analysis. The possibility of infection in patients cannot be completely avoided. Consequently, there is an urgent need to develop antibiotics that do not affect the efficacy of ICIs.

We found that the effect of PPIs on ICIs efficacy is also related to the exposure window and tumor type. The use of PPIs prior to the initiation of ICIs therapy significantly affects the prognosis of patients. The mechanism that underlies the effects on efficacy may involve changes in gut microbiota diversity and immune suppression caused by PPIs (51). However, compared with the direct effect of antibiotics, PPIs may indirectly change the gut microbiota by inhibiting stomach acid (52). Hopkins et al. (53) found that the negative impact of PPIs on prognosis may be related to the decrease in CD19+ and CD16+ CD56+ immune cell counts caused by PPIs. Nonetheless, it is also possible that the indication of PPIs is a poor prognostic factor for patients. PPIs exert statistically significant effects on NSCLC and UC, whereas they do not affect melanoma and RCC. This difference may be due to the smaller number of studies included in the analysis. Nevertheless, preclinical studies have shown that PPIs have an inhibitory effect on melanoma, which may counteract their negative effect on the gut microbiota (54). In addition, studies have found that chronic use of PPIs in patients with RCC is associated with an increased risk of ICI-induced colitis (55). Currently available evidence suggests that H2RAs do not reduce the benefits of ICIs therapy. However, caution is needed due to the small number of studies included in the analysis.

Steroids are commonly used to treat serious adverse events caused by ICIs and relieve clinical symptoms (56). Evidence suggests that the use of steroids reduces the efficacy of ICIs; however, this effect must be analyzed in different settings. For patients with brain metastases, although steroids decrease OS and PFS, there is no significant impact on intracranial-PFS. Subgroup analysis of NSCLC patients with brain metastases showed that steroids did not significantly reduce the OS after treatment with ICIs. In addition, the use of steroids in patients treated with ICIs in combination with SRS did not have a negative effect on efficacy. This may be due to the protective effect of SRS on the central nervous system and the reversal of local immune suppression (57). Steroids are also used for non-cancer indications (e.g., autoimmune diseases, chronic obstructive pulmonary disease, hypersensitivity reactions) and irAEs, which do not reduce the efficacy of ICIs, possibly because cancer indications (e.g., brain metastases, respiratory distress, bone metastases, and anorexia) are poor prognostic factors for patients (58).

Although preclinical studies have found that metformin can synergize with programmed death-1 (PD-1) inhibitors (59), some retrospective studies have shown that metformin does not affect the efficacy of ICIs. A recent study suggested that metformin exerts a significant synergistic effect compared with non-metformin hypoglycemic drugs (60). Another study yielded similar results; however, the use of dipeptidyl peptidase-4 (DDP4) inhibitor was identified as a potential confounding factor (61). In addition, whether the adjuvant effect of metformin is particularly important for obese patients should be further investigated (62). In this umbrella review, only one meta-analysis on metformin was included, which did not show an effect on the efficacy of ICIs. Overall, currently available evidence supports that metformin does not reduce the efficacy of ICIs. However, prospective studies with larger sample sizes and the elimination of confounding factors may be required to evaluate the potential enhancing effect of metformin on ICIs efficacy.

Our study showed weak evidence supporting that opioids may reduce the efficacy of ICIs. The possible mechanisms by which opioids exert this effect include immunosuppression of the tumor microenvironment (63, 64) and changes in the gut microbiome (65–67). Svaton et al. suggested that NSAIDs may enhance the efficacy of ICIs (68). Nevertheless, the currently available evidence does not indicate statistically significant effects. Further investigation is required to determine whether this is related to the timing and duration of NSAID use. In addition, clinicians should avoid the overuse of opioid drugs in patients receiving treatment with ICIs.

The present results indicated that β-blockers do not affect the efficacy of ICIs. Notably, apart from studies on β-blockers, there are currently no meta-analyses on angiotensin-converting enzyme (ACE) inhibitors and angiotensin II receptor blockers. A study suggested that ACE inhibitors may have a positive effect; however, this effect did not reach statistical significance (69). Another study indicated that the use of ACE inhibitors promotes immune suppression and reduces efficacy (70), while angiotensin II receptor blockers do not have a significant impact (71). Prospective studies with large sample sizes are warranted to confirm these findings.

Weak evidence suggests that statins and probiotics can improve the OS of patients receiving treatment with ICIs. Cantini et al. showed that high-intensity statins were associated with improved ICIs efficacy (72). In patients with NSCLC, statins have been linked to a trend for improvement in the efficacy of ICIs; however, this effect did not reach statistical significance. Statins may also improve ICIs efficacy by modulating the gut microbiome (73). In addition, preclinical studies have found that statins can lower the expression of PD-1 and cytotoxic T-lymphocyte associated protein-4 (CTLA-4) on T cells, increase antigen uptake by dendritic cells, and synergize with PD-1 inhibitors in terms of anti-tumor activity (74, 75). Gandhi and colleagues’ study showed that β blockers, metformin, aspirin, and statins had no effect on the efficacy of ICIs (76). In a mixed analysis group of multiple cancer types, probiotics did not exert a statistically significant effect; nevertheless, a trend towards enhanced efficacy was observed. In patients with NSCLC, probiotics may prolong OS and PFS; nevertheless, the evidence supporting this conclusion is currently weak. Dysbiosis of the gut microbiota is widely considered a key mechanism for the decreased efficacy of ICIs when used in combination with other drugs. Preclinical studies have highlighted that manipulation of the gut microbiota may improve the efficacy of ICIs (77). Additionally, further research is required to examine whether probiotics or statins can reverse the negative effects of other co-medications on ICIs.

This study has several limitations that should be acknowledged. Firstly, the methodological quality of the included publications was considered low or critically low. This low quality was mainly due to insufficient reporting of bias risks or the lack of a list of excluded literature. We partially addressed this limitation by re-evaluating bias risks. Secondly, due to the difficulty in conducting randomized controlled trials for concurrent medications, the included original studies were retrospective or prospective cohort studies; thus, bias risks are inevitable. There may be reverse causation; the use of antibiotics, PPIs, steroids, and opioids may be necessary due to the presence of poor prognostic factors in patients. This reverse causation may have affected the results. Therefore, caution is needed when interpreting the results. Thirdly, the impact of concurrent medications on the incidence of irAEs was not included in the meta-analyses; hence, future studies should address this topic. Finally, in addition to the concurrent medications included in our study, there are retrospective studies suggesting that vitamin D may improve ICIs efficacy (78). However, these studies were not included in our review due to the lack of meta-analyses. Therefore, more high-quality prospective studies are warranted to support the currently available evidence and avoid the impact of confounding factors.

For primary clinical studies and meta-analyses, more comprehensive evaluations are needed in the future, such as distinguishing the specific type and dose of concomitant medications, the patient’s performance status, and past disease history, so as to draw more accurate conclusions. Variations in concomitant medication effects on ICIs efficacy across cancer types require recognition, likely due to limited studies and differing ICIs regimens. These intriguing findings necessitate elucidation via rigorous clinical trials and preclinical studies. Additionally, disparate effects on other cancers, such as breast and endometrial cancer, warrant further investigation (79, 80).

## 5 Conclusions

Current evidence suggests that the use of antibiotics, PPIs, steroids, and opioids has a negative impact on the efficacy of ICIs. However, this effect may vary depending on the type of tumor, the timing of exposure, and the indication. Weak evidence suggests that statins and probiotics may enhance the efficacy of ICIs. H2 receptor antagonists, aspirin, metformin, β-blockers, and NSAIDs do not appear to affect the efficacy of ICIs. However, due to publication bias and methodological limitations, caution is advised in interpreting these results.

## Data availability statement

The original contributions presented in the study are included in the article/**Supplementary Material**. Further inquiries can be directed to the corresponding author.

## Author contributions

HLL: Conceptualization, Methodology, Software, Formal analysis, Data Curation, Writing - Original Draft, Visualization. LZ: Conceptualization, Methodology, Software, Visualization, Writing - Original Draft. FY: Data Curation, Project administration. RZ: Software, Visualization. XL Conceptualization, Validation, Supervision, Project administration. HJL: Conceptualization, Validation, Supervision, Project administration, Formal analysis, Validation, Supervision.

## Supplementary material

Supplementary Figure 1 AMSTAR 2 quality appraisal scores. AMSTAR, assessment of multiple systematic reviews. CDW, critical domains weaknesses (negatively answered); CL, critically low; L, low.

Supplementary Figure 2 Forest plot of the impact of exposure to antibiotics on ICIs efficacy, excluded due to overlap. CL, critically low; HR, hazard ratio; ICIs, immune checkpoint inhibitor; L, low; NSCLC, non-small cell lung cancer; OR, odds ratio; ORR, objective response rate; OS, overall survival; PFS, progression-free survival; RCC, renal cell carcinoma; CM, concomitant medications.

Supplementary Figure 3 Forest plot of the impact of exposure to PPIs on ICIs efficacy, excluded due to overlap. CL, critically low; HR, hazard ratio; ICIs, immune checkpoint inhibitor; L, low; NSCLC, non-small cell lung cancer; OS, overall survival; PFS, progression-free survival; PPI, proton pump inhibitor; UC, urothelial carcinoma; CM, concomitant medications.

Supplementary Figure 4 Forest plot of the impact of exposure to steroids, NSAIDs, β-blockers, probiotics, and opioids on ICIs efficacy, excluded due to overlap. CL, critically low; HR, hazard ratio; ICIs, immune checkpoint inhibitor; NSAIDs, nonsteroidal anti-inflammatory agents; NSCLC, non-small cell lung cancer; OR, odds ratio; ORR, objective response rate; OS, overall survival; PFS, progression-free survival; CM, concomitant medications.

Supplementary Figure 5 Funnel plots for publication bias regarding the exposure of patients with multiple types of cancer to ATB. (**A**. OS of ATB with any exposure window; **B**. PFS of ATB with any exposure window; **C**. ORR of ATB with any exposure window; **D**. RR of ATB with any exposure window; **E**. OS of ATB before ICIs initiation; **F**. PFS of ATB before ICIs initiation; **G**. ORR of ATB before ICIs initiation; **H**. OS of ATB during ICIs; I. PFS of ATB during ICIs; **J**. OS of ATB after ICIs initiation; **K**. PFS of ATB after ICIs initiation; **L**. PD of ATB before ICIs initiation >1 month; M. PD of ATB before ICIs initiation <1 month).

Supplementary Figure 6 Funnel plots for publication bias regarding the exposure of patients with NSCLC to ATB. (**A**. OS of ATB with any exposure window; **B**. PFS of ATB with any exposure window; **C**. OS of ATB before ICIs initiation; **D**. PFS of ATB before ICIs initiation; **E**. OS of ATB after ICIs initiation; **F**. PFS of ATB after ICIs initiation; **G**. OS of ATB during ICIs; **H**. PFS of ATB during ICIs).

Supplementary Figure 7 Funnel plots for publication bias regarding the exposure of patients with RCC, HCC, ESCC, or melanoma to ATB. (**A**. OS of ATB with any exposure window in RCC patients; **B**. PFS of ATB with any exposure window in RCC patients; **C**. ORR of ATB with any exposure window in RCC patients; **D**. OS of ATB with any exposure window in HCC patients; **E**. PFS of ATB with any exposure window in HCC patients; **F**. ORR of ATB with any exposure window in HCC patients; **G**. DCR of ATB with any exposure window in HCC patients; **H**. OS of ATB with any exposure window in ESCC patients; **I**. OS of ATB with any exposure window in melanoma patients).

Supplementary Figure 8 Funnel plots for publication bias regarding the exposure of patients with multiple types of cancer to PPIs/H2RAs. (**A**. OS of PPIs with any exposure window in multiple cancer types; **B**. PFS of PPIs with any exposure window in multiple cancer types; **C**. ORR of PPIs with any exposure window in multiple cancer types; **D**. OS of PPIs exposure [-60, NA] in multiple cancer types; **E**. PFS of PPIs exposure [-60, NA] in multiple cancer types; **F**. OS of PPIs exposure [0, NA] in multiple cancer types; **G**. PFS of PPIs exposure [0, NA] in multiple cancer types; **H**. OS of PPIs with any exposure window in NSCLC patients; **I**. PFS of PPIs with any exposure window in NSCLC patients; **J**. OS of PPIs with any exposure window in RCC patients; **K**. PFS of PPIs with any exposure window in RCC patients; **L**. OS of PPIs with any exposure window in melanoma patients; **M**. PFS of PPIs with any exposure window in melanoma patients; **N**. OS of PPIs with any exposure window in UC patients; **O**. PFS of PPIs with any exposure window in UC patients; **P**. ORR of H2RAs with any exposure window in multiple cancer types; **Q**. ORR of H2RAs with any exposure window in multiple cancer types).

Supplementary Figure 9 Funnel plots for publication bias regarding the exposure of patients with multiple types of cancer to steroids. (**A**. OS of steroids exposure in multiple cancer types; **B**. PFS of steroids exposure in multiple cancer types; **C**. OS of steroids exposure in multiple cancer types with brain metastasis patients; **D**. PFS of steroids exposure in multiple cancer types with brain metastasis patients; **E**. IC-PFS of steroids exposure in multiple cancer types with brain metastasis patients; **F**. OS of steroids exposure in multiple cancer types with brain metastasis patients no-receiving SRS; **G**. OS of steroids exposure in multiple cancer types with brain metastasis patients receiving SRS; **H**. OS of steroids exposure for cancer related indication in multiple cancer types; **I**. PFS of steroids exposure for cancer related indication in multiple cancer types; **J**. OS of steroids exposure for irAEs in multiple cancer types; **K**. PFS of steroids exposure for irAEs in multiple cancer types; **L**. OS of steroids exposure for non-cancer related indication in multiple cancer types; **M**. PFS of steroids exposure for non-cancer related indication in multiple cancer types; **N**. OS of steroids exposure in melanoma with brain metastasis patients; **O**. OS of steroids exposure in NSCLC with brain metastasis patients; **P**. OS of steroids exposure in NSCLC patients; **Q**. PFS of steroids exposure in NSCLC patients).

Supplementary Figure 10 Funnel plots for publication bias regarding the exposure of patients with multiple types of cancer to aspirin, β-blockers, metformin, NSAIDs, opioids, probiotics, and statins. NSAIDs, nonsteroidal anti-inflammatory agents. (**A**. OS of aspirins exposure in multiple cancer types; **B**. PFS of aspirins exposure in multiple cancer types; **C**. OS of β-blockers exposure in multiple cancer types; **D**. PFS of β-blockers exposure in multiple cancer types; **E**. OS of metformin exposure in multiple cancer types; **F**. PFS of metformin exposure in multiple cancer types; **G**. OS of NSAIDs exposure in multiple cancer types; **H**. PFS of NSAIDs exposure in multiple cancer types; **I**. ORR of NSAIDs exposure in multiple cancer types; **J**. OS of opioids exposure in multiple cancer types; **K**. PFS of opioids exposure in multiple cancer types; **L**. ORR of opioids exposure in multiple cancer types; **M**. OS of probiotics exposure in NSCLC patients; **N**. PFS of probiotics exposure in NSCLC patients; **O**. ORR of probiotics exposure in multiple cancer types; **P**. DCR of probiotics exposure in multiple cancer types; **Q**. OS of statins exposure in NSCLC patients; **R**. PFS of statins exposure in NSCLC patients; **S**. OS of statins exposure in multiple cancer types; **T**. PFS of statins exposure in multiple cancer types).
