# Supplementary material for: Impact of concomitant medications on the efficacy of immune checkpoint inhibitors: an umbrella review
Source: Front Immunol. 2023 Sep 29;14:1218386. doi: 10.3389/fimmu.2023.1218386 (PMC10570520; doi:10.3389/fimmu.2023.1218386)
Supplement: Supplementary file 1 [file DataSheet_1.zip › Supplementary_Materials/Table S5.docx]

**Table S5.** Detailed results and evidence of included meta-analyses regarding the effects of concomitant medications on the efficacy of ICIs.

| **Author** | **CM** | **ICIs treatment** | **Cancer type** | **No. of studies** | **No. of patients (CM+/CM-)** | **Outcome** | **Metric** | **Reported MA model** | **Reported p-Value** | **Reported SE (95% CI)** | **Reported I^2^(%) (p-Value)** | **Effect (95% CI) of largest study** | **consistence of individual study (Y/N)** | **RA Model** | **RA p-value** | **RA effect (95% CI)** | **RA I2** | **95% Prediction Interval** | **Egger p-Value** | **TES p-value** | **Level of evidence** |
| --- | --- | --- | --- | --- | --- | --- | --- | --- | --- | --- | --- | --- | --- | --- | --- | --- | --- | --- | --- | --- | --- |
| Chen, Baoqing 2022 | PPI | Anti-PD-(L)1; Anti-CTLA-4 | Multiple | 32 | 6969/7997* | OS | HR | Random | <0.001 | 1.31(1.19-1.44) | 72.08(0.04) | 0.96(0.89-1.04) | N | DL | 2.17E-08 | 1.31[1.192, 1.44] | 72.003 | [0.85, 2.017] | 0.0697 | 0.0000571 | III |
| Chen, Baoqing 2022 | PPI | Anti-PD-(L)1; Anti-CTLA-4 | Multiple | 24 | 3145/4417* | PFS | HR | Random | <0.001 | 1.30(1.17-1.46) | 62.92(0.04) | 1.26(1.07-1.48) | N | DL | 0.00000194 | 1.305[1.169, 1.456] | 62.982 | [0.852, 1.998] | 0.67 | 0.101 | III |
| Deng 2022 | PPI | Anti-PD-(L)1; Anti-CTLA-4 | Multiple | 15 | 1716/2260 | ORR | OR | Random | 0.15 | 0.81(0.60-1.08) | 68(<0.0001) | 0.72(0.55-0.95) | N | DL | 0.152 | 0.807[0.602, 1.082] | 67.499 | [0.308, 2.115] | 0.588 | 0.000941 | ns |
| Chen, Baoqing 2022 | PPI [-60,NA] | Anti-PD-(L)1; Anti-CTLA-4 | Multiple | 20 | 3153/4689 | OS | HR | Random | <0.001 | 1.35(1.22-1.51) | 58.2(NA) | 1.28(1.13-1.45) | N | DL | 2.78E-08 | 1.355[1.217, 1.508] | 57.971 | [0.94, 1.952] | 0.754 | 0.031 | II |
| Chen, Baoqing 2022 | PPI [-60,NA] | Anti-PD-(L)1; Anti-CTLA-4 | Multiple | 17 | 1886/3090* | PFS | HR | Random | <0.001 | 1.33(1.20-1.48) | 48(NA) | 1.33(1.20-1.48) | N | DL | 0.000000201 | 1.332[1.196, 1.484] | 48.019 | [0.959, 1.85] | 0.732 | 0.307 | II |
| Chen, Baoqing 2022 | PPI [0,NA] | Anti-PD-(L)1; Anti-CTLA-4 | Multiple | 12 | 2765/2165* | OS | HR | Random | 0.09 | 1.18(0.98-1.41) | 70.10(NA) | 0.96(0.89-1.04) | N | DL | 0.0853 | 1.174[0.978, 1.411] | 69.925 | [0.684, 2.017] | 0.241 | 0.139 | ns |
| Chen, Baoqing 2022 | PPI [0,NA] | Anti-PD-(L)1; Anti-CTLA-4 | Multiple | 7 | 294/330 | PFS | HR | Random | 0.58 | 1.19(0.65-2.17) | 83(NA) | 0.3(0.11-0.79) | N | DL | 0.578 | 1.186[0.649, 2.168] | 83.258 | [0.156, 9.024] | 0.88 | 0.477 | ns |
| Chen, Baoqing 2022 | PPI | Anti-PD-(L)1 | NSCLC | 16 | 4506/4404 | OS | HR | Random | <0.001 | 1.33(1.15-1.54) | 74.70(NA) | 0.96(0.89-1.04) | N | DL | 0.0000885 | 1.331[1.154, 1.535] | 74.62 | [0.825, 2.148] | 0.0371 | 0.00609 | III |
| Chen, Baoqing 2022 | PPI | Anti-PD-(L)1 | NSCLC | 13 | 1361/1969 | PFS | HR | Random | <0.001 | 1.33(1.17-1.51) | 43(NA) | 1.32(1.13-1.54) | N | DL | 0.0000172 | 1.326[1.166, 1.508] | 43.18 | [0.946, 1.858] | 0.52 | 0.61 | III |
| Chen, Baoqing 2022 | PPI | Anti-PD-(L)1; Anti-CTLA-4 | RCC | 6 | 198/207 | OS | HR | Random | 0.92 | 1.01(0.77-1.33) | 0(NA) | 0.81(0.53-1.24) | Y | DL | 0.917 | 1.015[0.774, 1.33] | 0 | [0.692, 1.488] | 0.586 | 0.66 | ns |
| Chen, Baoqing 2022 | PPI | Anti-PD-(L)1; Anti-CTLA-4 | RCC | 6 | 181/169 | PFS | HR | Random | 0.37 | 1.11(0.89-1.38) | 0(NA) | 1.05(0.73-1.51) | Y | DL | 0.372 | 1.106[0.886, 1.381] | 0 | [0.808, 1.515] | 0.533 | 0.722 | ns |
| Chen, Baoqing 2022 | PPI | Anti-PD-(L)1; Anti-CTLA-4 | Melanoma | 5 | 149/328* | OS | HR | Random | 0.16 | 1.39(0.87-2.22) | 66.4(NA) | 1.83(1.20-2.79) | N | DL | 0.167 | 1.392[0.871, 2.224] | 66.627 | [0.307, 6.318] | 0.0794 | 0.57 | ns |
| Chen, Baoqing 2022 | PPI | Anti-PD-(L)1; Anti-CTLA-4 | Melanoma | 4 | 63/182* | PFS | HR | Random | 0.75 | 0.89(0.44-1.81) | 83.3(NA) | 1.62(1.20-2.19) | N | HKSJ | 0.785 | 0.889[0.254, 3.113] | 83.317 | [0.034, 23.193] | 0.244 | 0.0711 | ns |
| Chen, Baoqing 2022 | PPI | Anti-PD-1 | Multiple | 10 | 1146/1422 | OS | HR | Fixed | <0.01 | 1.36(1.12,1.64) | 47.5(NA) | [1.258, 1.769] | N | DL | 0.0008 | 1.368[1.139, 1.643] | 45.873 | [0.842, 2.223] | 0.582 | 0.422 | IV |
| Chen, Baoqing 2022 | PPI | Anti-PD-1 | Multiple | 9 | 854/1066* | PFS | HR | Fixed | <0.001 | 1.32(1.16,1.51) | 17.5(NA) | [1.13, 1.54] | N | DL | 0.0000362 | 1.323[1.027, 1.704] | 16.789 | [1.027, 1.704] | 0.802 | 0.478 | IV |
| Chen, Baoqing 2022 | PPI | Anti-PD-L1 | Multiple | 3 | 810/1591 | OS | HR | Fixed | <0.001 | 1.50(1.33,1.68) | 0(NA) | [1.27, 1.82] | Y | HKSJ | 0.00181 | 1.496[1.39, 1.611] | 0 | [1.203, 1.861] | 0.761 | 0.371 | IV |
| Chen, Baoqing 2022 | PPI | Anti-PD-L1 | Multiple | 3 | 810/1591 | PFS | HR | Fixed | <0.001 | 1.34(1.22,1.48) | 0(NA) | [1.179, 1.619] | Y | HKSJ | 0.00393 | 1.341[1.239, 1.452] | 0 | [1.061, 1.695] | 0.835 | 0.311 | IV |
| Chen, Baoqing 2022 | PPI | Anti-PD-(L)1 | Multiple | 9 | 3884/3144 | OS | HR | Random | <0.01 | 1.28(1.07,1.52) | 74.73(NA) | [0.889, 1.039] | N | DL | 0.00592 | 1.278[1.073, 1.522] | 74.825 | [0.776, 2.104] | 0.0492 | 0.0000188 | IV |
| Chen, Baoqing 2022 | PPI | Anti-PD-(L)1 | Multiple | 7 | 902/846 | PFS | HR | Random | <0.001 | 1.52(1.13,2.03) | 71.5(NA) | [1.071, 1.481] | N | DL | 0.00506 | 1.516[1.133, 2.028] | 71.525 | [0.633, 3.63] | 0.226 | 0.311 | ns |
| Chen, Baoqing 2022 | PPI | Anti-CTLA4 | Melanoma | 1 | 17/63 | OS | HR | Fixed | 0.09 | 0.44(0.17,1.14) | NA | [0.17, 1.14] | Y | HKSJ | 0.091 | 0.44[0.17, 1.14] | only 1 study | < 3 studies | < 3 studies | NA | ns |
| Chen, Baoqing 2022 | PPI | Anti-CTLA4 | Melanoma | 1 | 17/63 | PFS | HR | Fixed | 0.92 | 1.04(0.49,2.20) | NA | [0.34, 1.06] | Y | HKSJ | 0.0785 | 0.6[0.34, 1.06] | only 1 study | < 3 studies | < 3 studies | NA | ns |
| Zhang 2022 | PPI | Anti-PD-(L)1 | UC | 6 | 647/1028 | OS | HR | Fixed | NA | 1.59(1.37-1.84) | 37.4(0.157) | 1.52(1.27-1.83) | N | DL | 0.0000893 | 1.638[1.28, 2.097] | 38.308 | [0.882, 3.042] | 0.584 | 0.0000856 | IV |
| Zhang 2022 | PPI | Anti-PD-(L)1 | UC | 6 | 647/1028 | PFS | HR | Fixed | NA | 1.51(1.33-1.71) | 7.4(0.369) | 1.38(1.18-1.62) | N | DL | 5.06E-09 | 1.535[1.33, 1.773] | 7.13 | [1.192, 1.977] | 0.0331 | 0.000348 | IV |
| Deng 2022 | H2RAs | Anti-PD-(L)1 | Multiple | 3 | 1451/2938 | OS | HR | Random | 0.81 | 1.07(0.61-1.87) | 85(0.001) | 1.65(1.46-1.87) | N | HKSJ | 0.838 | 1.071[0.301, 3.817] | 85.496 | [0.001, 956.346] | 0.0701 | 0.764 | ns |
| Deng 2022 | H2RAs | Anti-PD-(L)1 | Multiple | 2 | 430/741 | PFS | HR | Fixed | 0.93 | 1.02(0.72-1.42) | 21(0.26) | 1.05(0.74-1.45) | N | HKSJ | 0.833 | 0.905[0.008, 104.309] | 19.753 | < 3 studies | < 3 studies | NA | ns |
| Deng 2022 | H2RAs | Anti-PD-(L)1 | Multiple | 3 | 76/839 | ORR | OR | Fixed | 0.82 | 1.06(0.64-1.74) | 52(0.13) | 0.84(0.46-1.52) | Y | HKSJ | 0.618 | 1.323[0.169, 10.334] | 51.795 | [0.169, 10.334] | 0.385 | 0.0932 | ns |
| Wu 2021 | ATB | Anti-PD-(L)1; Anti-CTLA-4 | Multiple | 38 | 3019/9287* | OS | HR | Random | NA | 1.20(1.15-1.25) | 84(<0.01) | 1.07(0.77-1.48) | N | DL | 1.17E-15 | 1.198[1.146, 1.253] | 83.962 | [0.956, 1.503] | 0.000000401 | 0 | III |
| Wu 2021 | ATB | Anti-PD-(L)1; Anti-CTLA-4 | Multiple | 31 | 1663/4454* | PFS | HR | Random | NA | 1.18(1.11-1.25) | 74(0.0205) | 1.07(0.99-1.16) | N | DL | 1.81E-08 | 1.174[1.11, 1.242] | 73.038 | [0.877, 1.572] | 0.00808 | 5.54E-11 | II |
| Tsikala 2021 | ATB | Anti-PD-(L)1; Anti-CTLA-4 | Multiple | 18 | 564/1556 | RR | OR | Random | <0.009 | 0.54(0.34-0.86) | 62(0.0003) | 0.48(0.19-1.20) | N | DL | 0.0159 | 0.576[0.368, 0.902] | 59.456 | [0.116, 2.857] | 0.377 | 0.11 | IV |
| Wu 2021 | ATB | Anti-PD-(L)1; Anti-CTLA-4 | Multiple | 17 | 543/1547 | ORR | OR | Random | NA | 0.61(0.42-0.90) | 43(0.03) | 0.48(0.19-1.20) | N | DL | 0.0188 | 0.64[0.441, 0.929] | 38.086 | [0.22, 1.865] | 0.686 | 0.212 | IV |
| Wu 2021 | ATB (Before ICIs initiation) | Anti-PD-(L)1; Anti-CTLA-4 | Multiple | 14 | 520/1885 | OS | HR | Random | NA | 1.39(1.26-1.54) | 67(<0.01) | 1.34(1.17-1.53) | N | DL | 3.76E-10 | 1.391[1.255, 1.542] | 67.546 | [0.973, 1.99] | 0.426 | 0.491 | IV |
| Wu 2021 | ATB (Before ICIs initiation) | Anti-PD-(L)1; Anti-CTLA-4 | Multiple | 13 | 382/1349 | PFS | HR | Random | NA | 1.23(1.14-1.32) | 42(0.05) | 1.12(0.96-1.3) | N | DL | 4.24E-08 | 1.226[1.14, 1.319] | 42.419 | [1.004, 1.497] | 0.0657 | 0.025 | IV |
| Wu 2021 | ATB (Before ICIs initiation) | Anti-PD-(L)1; Anti-CTLA-4 | Multiple | 8 | 249/811 | ORR | OR | Fixed | NA | 0.47(0.32-0.71) | 0(0.72) | 0.48(0.19-1.20) | N | DL | 0.0021 | 0.527[0.35, 0.793] | 0 | [0.316, 0.877] | 0.983 | 0.675 | IV |
| Tsikala 2021 | ATB (within 1month prior to ICIs) | Anti-PD-(L)1; Anti-CTLA-4 | Multiple | 7 | 156/753 | PD | OR | Random | 0.15 | 1.67(0.83-3.33) | 67(0.006) | 1.44(0.77-2.73) | N | DL | 0.147 | 1.667[0.835, 3.329] | 66.974 | [0.202, 13.777] | 0.742 | 0.756 | ns |
| Tsikala 2021 | ATB (> 1month prior to ICIs) | Anti-PD-(L)1; Anti-CTLA-4 | Multiple | 5 | 215/585 | PD | OR | Random | 0.002 | 2.5(1.40-4.44) | 58(0.05) | 3.42(1.51-7.77) | N | DL | 0.00182 | 2.497[1.405, 4.438] | 58.163 | [0.402, 15.49] | 0.983 | 0.0136 | IV |
| Huang, Litang 2021 | ATB (During ICIs use) | Anti-PD-(L)1; Anti-CTLA-4 | Multiple | 5 | 269/457* | PFS | HR | Random | NR | 0.78(0.65-0.93) | 12.1(0.337) | 0.70(0.50-0.98) | N | DL | 0.00602 | 0.779[0.652, 0.931] | 11.923 | [0.54, 1.123] | 0.252 | 0.194 | IV |
| Huang, Litang 2021 | ATB (During ICIs use) | Anti-PD-(L)1; Anti-CTLA-4 | Multiple | 5 | 152/536* | OS | HR | Random | NR | 0.98(0.78-1.24) | 24.9(0.255) | 1.10(0.75-1.60) | Y | DL | 0.839 | 0.977[0.777, 1.228] | 27.246 | [0.552, 1.729] | 0.345 | 0.702 | ns |
| Zhou 2022 | ATB [0,30] | Anti-PD-(L)1; Anti-CTLA-4 | Multiple | 3 | 74/360 | OS | HR | Random | 0.002 | 2.44(1.38-4.34) | 7(0.30) | 4.4(2.95-6.57) | N | HKSJ | 0.0425 | 3.264[1.104, 9.653] | 49.91 | [0.017, 621.446] | 0.522 | 0.865 | IV |
| Zhou 2022 | ATB [0,30] | Anti-PD-(L)1; Anti-CTLA-4 | Multiple | 3 | 74/360 | PFS | HR | Random | 0.005 | 2.38(1.30-4.36) | 73(0.02) | 1.5(1.1-2.04) | Y | HKSJ | 0.0892 | 2.382[0.719, 7.885] | 73.053 | [0.003, 2034.15] | 0.0895 | 0.00112 | ns |
| Wu 2021 | ATB | Anti-PD-(L)1; Anti-CTLA-4 | NSCLC | 27 | 1232/4492* | OS | HR | Random | NA | 1.26(1.15-1.38) | 79(<0.01) | 1.07(0.77-1.48) | N | DL | 0.00000113 | 1.255[1.146, 1.376] | 78.405 | [0.814, 1.937] | 0.0264 | 0.0000441 | III |
| Wu 2021 | ATB | Anti-PD-(L)1; Anti-CTLA-4 | NSCLC | 24 | 1081/2634* | PFS | HR | Random | NA | 1.13(1.04-1.23) | 78(<0.01) | 1.07(0.99-1.16) | N | DL | 0.00283 | 1.132[1.044, 1.229] | 78.141 | [0.783, 1.637] | 0.129 | 0.000146 | IV |
| Lurienne 2020 | ATB [0,60] | Anti-PD-(L)1 | NSCLC | 3 | 97/2077 | OS | HR | Random | NR | 2.94(1.60-5.40) | 0(0.72) | 3.41(1.39-8.39) | N | HKSJ | 0.0168 | 2.935[1.597, 5.395] | 0 | [0.487, 17.709] | 0.219 | 0.0305 | IV |
| Lurienne 2020 | ATB [0,60] | Anti-PD-(L)1 | NSCLC | 3 | 73/252 | PFS | HR | Random | NR | 2.00(1.34-2.99) | 0.0(0.007) | 1.63(0.71-3.72) | N | HKSJ | 0.0164 | 2.003[1.359, 2.952] | 0 | [0.638, 6.295] | 0.974 | 0.356 | IV |
| Chen 2021 | ATB (During ICIs use) | Anti-PD-(L)1; Anti-CTLA-4 | NSCLC | 6 | 193/713 | OS | HR | Random | NR | 1.87(1.43-2.44) | 69.9(0.005) | 1.74(0.75-3.99) | N | DL | 0.00224 | 2.334[1.355, 4.018] | 69.742 | [0.425, 12.804] | 0.146 | 0.346 | IV |
| Chen 2021 | ATB (During ICIs use) | Anti-PD-(L)1; Anti-CTLA-4 | NSCLC | 5 | 142/568 | PFS | HR | Random | NR | 1.20(0.99-1.45) | 65.5(0.021) | 0.86(0.61-1.22) | N | DL | 0.198 | 1.257[0.887, 1.782] | 60.355 | [0.418, 3.783] | 0.451 | 0.513 | ns |
| Crespin 2021 | ATB [-60,0] | Anti-PD-(L)1; Anti-CTLA-4 | NSCLC | 13 | 1318/4926 | OS | HR | Random | NR | 2.36(1.68-3.31) | 80(<0.01) | 1.31(1.20-1.44) | N | DL | 1.99E-09 | 2.277[1.74, 2.98] | 79.811 | [0.944, 5.494] | 0.000583 | 1.41E-10 | II |
| Crespin 2021 | ATB [-60,0] | Anti-PD-(L)1; Anti-CTLA-4 | NSCLC | 9 | 388/1686 | PFS | HR | Random | NR | 1.75(1.29-2.37) | 53(0.03) | 1.29(1.04-1.60) | N | DL | 0.0000132 | 1.677[1.329, 2.116] | 52.568 | [0.904, 3.11] | 0.00855 | 0.000138 | IV |
| Crespin 2021 | ATB | Anti-PD-(L)1; Anti-CTLA-4 | NSCLC | 10 | 718/2556 | ORR | OR | Random | NR | 0.66(0.44-0.99) | 3(0.42) | 0.57(0.37-0.87) | Y | DL | 0.0234 | 0.675[0.481, 0.948] | 15.89 | [0.356, 1.28] | 0.649 | 0.696 | IV |
| Wu 2021 | ATB | Anti-PD-(L)1; Anti-CTLA-4 | Melanoma | 4 | 147/1672* | OS | HR | Random | NA | 1.36(1.06-1.75) | 44(0.14) | 0.86(0.47-1.57) | N | HKSJ | 0.136 | 1.36[0.839, 2.205] | 44.421 | [0.509, 3.638] | 0.891 | 0.29 | ns |
| Wu 2021 | ATB | Anti-PD-(L)1; Anti-CTLA-4 | Melanoma | 2 | 10/64* | PFS | HR | Fixed | NA | 1.75(1.34-2.29) | 0(0.68) | 1.84(1.28-2.63) | Y | HKSJ | 0.0634 | 1.749[0.86, 3.556] | 0 | < 3 studies | < 3 studies | NA | ns |
| Wu 2021 | ATB | Anti-PD-(L)1; Anti-CTLA-4 | Melanoma | 2 | 56/126 | ORR | OR | Fixed | NA | 0.37(0.12-1.10) | 40(0.20) | 0.64(0.18-2.28) | N | HKSJ | 0.089 | 0.628[0.273, 1.444] | 0 | [0.273, 1.444] | < 3 studies | NA | ns |
| Wu 2021 | ATB | Anti-PD-(L)1; Anti-CTLA-4 | RCC | 5 | 100/424 | OS | HR | Fixed | NA | 1.12(1.01-1.25) | 0(0.62) | 1.17(0.88-1.56) | Y | DL | 0.028 | 1.122[1.013, 1.244] | 0 | [0.95, 1.326] | 0.0242 | 0.838 | IV |
| Wu 2021 | ATB | Anti-PD-(L)1; Anti-CTLA-4 | RCC | 7 | 145/465 | PFS | HR | Fixed | NA | 1.29(1.19-1.40) | 4(0.40) | 1.34(1.08-1.66) | Y | DL | 1.19E-09 | 1.299[1.194, 1.413] | 5.288 | [1.139, 1.481] | 0.00228 | 7.11E-08 | IV |
| Wu 2021 | ATB | Anti-PD-(L)1; Anti-CTLA-4 | RCC | 4 | 63/304 | ORR | OR | Fixed | NA | 0.30(0.14-0.67) | 0(0.97) | 0.28(0.09-0.85) | N | HKSJ | 0.00291 | 0.318[0.212, 0.477] | 0 | [0.212, 0.477] | 0.466 | 0.544 | IV |
| Wu 2021 | ATB | Anti-PD-(L)1 | Multiple | 22 | 1213/2749* | OS | HR | Random | NA | 1.22(1.12,1.32) | 87(<0.01) | [0.042, 0.132] | N | DL | 0.00000787 | 1.216[1.116, 1.325] | 87.437 | [0.847, 1.746] | 0.00194 | 0 | III |
| Wu 2021 | ATB | Anti-PD-(L)1 | Multiple | 23 | 1205/2738* | PFS | HR | Random | NA | 1.16(1.07,1.26) | 81(<0.01) | [0.012, 0.092] | N | DL | 0.000292 | 1.162[1.071, 1.26] | 80.639 | [0.818, 1.651] | 0.0918 | 1.97E-12 | III |
| Wu 2021 | ATB | Anti-PD-(L)1 plus Anti-CTLA-4 | Multiple | 11 | 1004/1789 | OS | HR | Fixed | NA | 1.25(1.19,1.33) | 15(0.30) | [0.048, 0.162] | N | DL | 2.09E-13 | 1.26[1.185, 1.34] | 13.137 | [1.128, 1.408] | 0.552 | 0.772 | I |
| Wu 2021 | ATB | Anti-PD-(L)1 plus Anti-CTLA-4 | Multiple | 10 | 326/1104 | PFS | HR | Fixed | NA | 1.18(1.11,1.25) | 32(0.16) | [-0.034, 0.119] | N | DL | 0.00000639 | 1.172[1.094, 1.255] | 16.642 | [1.027, 1.337] | 0.00074 | 0.321 | IV |
| Luo 2022 | ATB | Anti-PD-(L)1; Anti-CTLA-4 | RCC | 2 | 133/655 | PD | OR | Fixed | NA | 1.18(0.97-1.44) | 44(0.18) | 1.12(0.91-1.38) | Y | HKSJ | 0.316 | 1.32[0.195, 8.919] | 0 | [0.195, 8.919] | < 3 studies | NA | ns |
| Wu 2021 | ATB | Anti-PD-(L)1 | UC | 2 | 247/642 | OS | HR | Fixed | NA | 1.17(1.09-1.27) | 0(0.83) | 1.17(1.08-1.27) | N | HKSJ | 0.0373 | 1.174[1.042, 1.323] | 0 | < 3 studies | < 3 studies | NA | IV |
| Wu 2021 | ATB | Anti-PD-(L)1 | UC | 2 | 247/642 | PFS | HR | Fixed | NA | 1.11(1.03-1.19) | 23(0.25) | 1.1(1.02-1.18) | N | HKSJ | 0.331 | 1.132[0.459, 2.793] | 23.195 | < 3 studies | < 3 studies | NA | ns |
| Zhang, Lilong 2022 | ATB | Anti-PD-(L)1; Anti-CTLA-4 | HCC | 5 | 332/684 | OS | HR | Random | NA | 1.41(0.96-2.08) | 62(0.032) | 0.96(0.73-1.26) | N | DL | 0.0821 | 1.407[0.957, 2.067] | 61.335 | [0.426, 4.648] | 0.419 | 0.597 | ns |
| Zhang, Lilong 2022 | ATB | Anti-PD-(L)1; Anti-CTLA-4 | HCC | 4 | 223/398 | PFS | HR | Random | NA | 1.21(0.73-2.00) | 72.7(0.012) | 0.75(0.60-0.94) | N | HKSJ | 0.451 | 1.224[0.582, 2.573] | 73.773 | [0.149, 10.054] | 0.0218 | 0.0154 | ns |
| Zhang, Lilong 2022 | ATB | Anti-PD-(L)1; Anti-CTLA-4 | HCC | 4 | NA | ORR | OR | NA | NA | 1.06(0.69-1.64) | 0(0.408) | 1.3(0.79-2.13) | Y | HKSJ | 0.771 | 1.073[0.533, 2.158] | 0 | [0.533, 2.158] | 0.153 | 0.647 | ns |
| Zhang, Lilong 2022 | ATB | Anti-PD-(L)1; Anti-CTLA-4 | HCC | 3 | NA | DCR | OR | NA | NA | 0.42(0.09-2.06) | 83.2(0.003) | 1.36(0.92-2.01) | N | HKSJ | 0.348 | 0.423[0.02, 8.897] | 83.447 | [0.02, 8.897] | 0.132 | 0.363 | ns |
| Zhou 2022 | ATB | Anti-PD-(L)1 | ESCC | 3 | 83/127* | OS | HR | Random | 0.03 | 2.80(1.08-7.25) | 90(<0.001) | 1.26(0.95-1.67) | N | HKSJ | 0.141 | 2.795[0.435, 17.972] | 90.204 | [0, 271592.82] | 0.0867 | 0.222 | ns |
| Wang 2021 | Steroids | Anti-PD-(L)1; Anti-CTLA-4 | Multiple | 24 | 773/4423* | PFS | HR | Random | NA | 1.51(1.25-1.82) | 75.9(<0.0001) | 1.31(1.07-1.62) | N | DL | 0.0000233 | 1.505[1.245, 1.819] | 76.373 | [0.669, 3.386] | 0.72 | 0.00000009 | III |
| Wang 2021 | Steroids | Anti-PD-(L)1; Anti-CTLA-4 | Multiple | 36 | 1976/6722* | OS | HR | Random | NA | 1.51(1.33-1.73) | 70.6(<0.0001) | 1.46(1.16-1.84) | N | DL | 6.26E-10 | 1.513[1.327, 1.725] | 70.646 | [0.839, 2.727] | 0.15 | 0.000281 | II |
| Jessurun 2021 | Steroids | Anti-PD-(L)1; Anti-CTLA-4 | Multiple Brain metastatic | 14 | 349/730* | OS | HR | Random | 0.007 | 1.84(1.22-2.77) | 50(0.02) | 2.37(0.83-6.74) | N | DL | 0.000125 | 1.838[1.347, 2.508] | 49.895 | [0.76, 4.446] | 0.747 | 0.59 | IV |
| Jessurun 2021 | Steroids | Anti-PD-(L)1; Anti-CTLA-4 | Multiple Brain metastatic | 5 | 166/365 | PFS | HR | Random | 0.007 | 2.00(1.37-2.91) | 0(0.75) | 2.78(0.93-8.27) | N | DL | 0.000694 | 1.981[1.335, 2.942] | 0 | [1.043, 3.764] | 0.868 | 0.705 | IV |
| Jessurun 2021 | Steroids | Anti-PD-(L)1; Anti-CTLA-4 | Multiple Brain metastatic | 4 | 81/187 | IC-PFS | HR | Random | 0.5 | 1.31(0.42-4.07) | 53(0.09) | 1.97(0.94-4.15) | N | HKSJ | 0.374 | 1.309[0.723, 2.369] | 52.79 | [0.135, 12.645] | 0.51 | 0.867 | ns |
| Jessurun 2021 | Steroids | Anti-PD-(L)1; Anti-CTLA-4 | Melanoma Brain metastatic | 8 | 143/281* | OS | HR | Random | NA | 1.67(1.49-1.87) | 0(0.98) | 1.69(1.29-2.22) | N | DL | 0.000000728 | 1.671[1.364, 2.047] | 0 | [1.297, 2.153] | 0.392 | 0.671 | IV |
| Jessurun 2021 | Steroids | Anti-PD-(L)1; Anti-CTLA-4 | NSCLC Brain metastatic | 5 | 137/369 | OS | HR | Random | NA | 2.43(0.38-15.77) | 82(<0.01) | 2.37(0.83-6.74) | N | DL | 0.163 | 2.435[0.698, 8.493] | 82.111 | [0.025, 236.032] | 0.192 | 0.79 | ns |
| Jessurun 2021 | Steroids non-SRS | Anti-PD-(L)1; Anti-CTLA-4 | Multiple Brain metastatic | 11 | 235/564* | OS | HR | Random | NA | 1.97(1.28-3.05) | 38(0.09) | 2.37(0.83-6.74) | N | DL | 0.0000773 | 1.974[1.409, 2.766] | 38.333 | [0.874, 4.46] | 0.347 | 0.556 | IV |
| Jessurun 2021 | Steroids SRS | Anti-PD-(L)1; Anti-CTLA-4 | Multiple Brain metastatic | 3 | 114/166 | OS | HR | Random | NA | 1.30(0.11-14.77) | 79(<0.01) | 2.46(1.44-4.20) | N | HKSJ | 0.689 | 1.299[0.115, 14.722] | 79.315 | [0, 124557.298] | 0.0106 | 0.602 | ns |
| Wang 2021 | Steroids cancer indication | Anti-PD-(L)1; Anti-CTLA-4 | Multiple | 12 | 584/2559* | OS | HR | Random | NA | 1.94(1.59-2.36) | 51.5(0.02) | 1.46(1.16-1.84) | N | DL | 7.98E-11 | 1.937[1.587, 2.364] | 51.542 | [1.133, 3.311] | 0.526 | 0.0693 | II |
| Wang 2021 | Steroids cancer indication | Anti-PD-(L)1; Anti-CTLA-4 | Multiple | 8 | 375/2172* | PFS | HR | Random | NA | 1.73(1.38-2.18) | 64.4(0.006) | 1.31(1.07-1.62) | N | DL | 0.00000206 | 1.737[1.383, 2.183] | 64.363 | [0.922, 3.274] | 0.0297 | 0.00000339 | III |
| Wang 2021 | Steroids non-cancer indication | Anti-PD-(L)1; Anti-CTLA-4 | Multiple | 4 | 95/681* | OS | HR | Random | NA | 0.79(0.51-1.21) | 36.4(0.194) | 0.91(0.47-1.79) | N | HKSJ | 0.374 | 0.796[0.396, 1.599] | 29.557 | [0.203, 3.113] | 0.961 | 0.268 | ns |
| Wang 2021 | Steroids non-cancer indication | Anti-PD-(L)1; Anti-CTLA-4 | Multiple | 4 | 95/681* | PFS | HR | Random | NA | 0.83(0.64-1.07) | 0(0.407) | 0.96(0.68-1.36) | Y | HKSJ | 0.214 | 0.807[0.523, 1.246] | 10.708 | [0.395, 1.646] | 0.466 | 0.787 | ns |
| Wang 2021 | Steroids cancer irAEs | Anti-PD-(L)1; Anti-CTLA-4 | Multiple | 5 | 123/448 | PFS | HR | Random | NA | 1.30(0.63-2.70) | 75.9(<0.0001) | 0.66(0.36-1.22) | N | DL | 0.483 | 1.3[0.625, 2.707] | 80.391 | [0.092, 18.317] | 0.897 | 0.00000133 | ns |
| Wang 2021 | Steroids cancer irAEs | Anti-PD-(L)1; Anti-CTLA-4 | Multiple | 9 | 461/725* | OS | HR | Random | NA | 1.11(0.83-1.47) | 31.7(0.165) | 1.69(0.58-4.94) | N | DL | 0.563 | 1.083[0.827, 1.419] | 25.101 | [0.612, 1.916] | 0.169 | 0.256 | ns |
| Wang 2021 | Steroids | Anti-PD-(L)1 | Multiple | 11 | 248/1535* | PFS | HR | Random | <0.001 | 1.60(1.24,2.064) | 75.6 | [1.43, 2.07] | N | DL | 0.00086 | 1.586[1.209, 2.081] | 77.583 | [0.639, 3.935] | 0.945 | 0.00000177 | III |
| Wang 2021 | Steroids | Anti-CTLA-4 | Melanoma | 1 | 14/50 | PFS | HR | Random | 0.019 | 2.780(1.300,7.140) | NA | [1.135, 7.123] | Y | HKSJ | 0.0257 | 2.844[1.135, 7.123] | only 1 study | < 3 studies | < 3 studies | NA | IV |
| Wang 2021 | Steroids | Anti-PD-(L)1 | Multiple | 14 | 889/3276 | OS | HR | Random | <0.001 | 1.509(1.256,1.813) | 69.7 | [1.76, 2.65] | N | DL | 2.28E-08 | 1.643[1.38, 1.955] | 64.21 | [0.947, 2.848] | 0.613 | 0.0699 | II |
| Wang 2021 | Steroids | Anti-CTLA-4 | Melanoma | 6 | 214/399 | OS | HR | Random | 0.059 | 1.726(0.979,3.042) | 73.4 | [0.739, 1.41] | N | DL | 0.0635 | 1.706[0.97, 3.001] | 72.378 | [0.302, 9.635] | 0.186 | 0.256 | ns |
| Zhang, Hongchuan 2021 | Steroids | Anti-PD-(L)1; Anti-CTLA-4 | NSCLC | 11 | 1009/3433* | OS | HR | Random | NA | 1.82(1.51-2.18) | 62.6(0.003) | 1.46(1.16-1.84) | N | DL | 1.01E-10 | 1.816[1.516, 2.177] | 62.489 | [1.054, 3.13] | 0.0498 | 0.0000252 | II |
| Zhang, Hongchuan 2021 | Steroids | Anti-PD-(L)1; Anti-CTLA-4 | NSCLC | 11 | 607/3060 | PFS | HR | Random | NA | 1.69(1.41-2.04) | 57.3(0.009) | 1.31(1.06-1.61) | N | DL | 3.37E-08 | 1.695[1.405, 2.044] | 57.385 | [0.981, 2.929] | 0.0152 | 2.68E-09 | IV |
| Zhang, Yongchao 2021 | Statins | Anti-PD-(L)1; Anti-CTLA-4 | Multiple | 6 | 383/1482 | OS | HR | Fixed | 0.005 | 0.76(0.63-0.92) | 0(0.43) | 0.79(0.62-1.01) | N | DL | 0.0054 | 0.761[0.627, 0.922] | 0 | [0.579, 0.999] | 0.415 | 0.57 | IV |
| Zhang, Yongchao 2021 | Statins | Anti-PD-(L)1; Anti-CTLA-4 | Multiple | 6 | 383/1482 | PFS | HR | Fixed | 0.036 | 0.86(0.75-0.99) | 49.34(0.08) | 0.87(0.72-1.06) | N | DL | 0.0671 | 0.795[0.622, 1.016] | 49.322 | [0.412, 1.536] | 0.148 | 0.145 | ns |
| Zhang, Lei 2022 | Statins | Anti-PD-(L)1; Anti-CTLA-4 | NSCLC | 8 | 513/1869 | PFS | HR | Random | 0.17 | 0.86(0.70-1.07) | 62(0.009) | 0.99(0.83-1.18) | N | DL | 0.173 | 0.863[0.698, 1.067] | 62.28 | [0.464, 1.606] | 0.647 | 0.0541 | ns |
| Zhang, Lei 2022 | Statins | Anti-PD-(L)1; Anti-CTLA-4 | NSCLC | 7 | 503/1812 | OS | HR | Random | 0.07 | 0.86(0.74-1.01) | 29(0.20) | 1.06(0.88-1.28) | Y | DL | 0.074 | 0.865[0.739, 1.014] | 29.018 | [0.609, 1.23] | 0.312 | 0.522 | ns |
| Zhang, Yongchao 2021 | Aspirins | Anti-PD-(L)1; Anti-CTLA-4 | Multiple | 4 | 317/1401 | OS | HR | Fixed | 0.514 | 0.93(0.76-1.15) | 9.24(0.35) | 0.85(0.67-1.07) | Y | HKSJ | 0.802 | 0.967[0.651, 1.434] | 10.272 | [0.651, 1.434] | 0.18 | 0.706 | ns |
| Zhang, Yongchao 2021 | Aspirins | Anti-PD-(L)1; Anti-CTLA-4 | Multiple | 5 | 378/1557 | PFS | HR | Fixed | 0.024 | 0.84(0.72-0.98) | 38.3(0.17) | 0.79(0.64-0.98) | N | DL | 0.174 | 0.86[0.693, 1.069] | 37.279 | [0.48, 1.543] | 0.356 | 0.0772 | ns |
| Zhang, Yongchao 2021 | Metformin | Anti-PD-(L)1; Anti-CTLA-4 | Multiple | 8 | 266/2133 | OS | HR | Fixed | 0.46 | 1.07(0.89-1.30) | 40.28(0.11) | 1.24(0.95-1.61) | Y | DL | 0.962 | 0.993[0.744, 1.326] | 40.22 | [0.488, 2.021] | 0.227 | 0.819 | ns |
| Zhang, Yongchao 2021 | Metformin | Anti-PD-(L)1; Anti-CTLA-4 | Multiple | 7 | 249/1778 | PFS | HR | Fixed | 0.51 | 1.08(0.92-1.27) | 29.24(0.21) | 1.13(0.89-1.43) | Y | DL | 0.537 | 1.072[0.86, 1.336] | 29.342 | [0.652, 1.763] | 0.848 | 0.796 | ns |
| Yan 2022 | Beta blockers | Anti-PD-(L)1; Anti-CTLA-4 | Multiple | 11 | 3418/6817 | OS | HR | Random | NA | 0.97(0.85-1.11) | 56.5(0.011) | 1.39(1.1-1.76) | N | DL | 0.68 | 0.973[0.855, 1.108] | 56.43 | [0.666, 1.421] | 0.0907 | 0.225 | ns |
| Yan 2022 | Beta blockers | Anti-PD-(L)1; Anti-CTLA-4 | Multiple | 7 | 1252/4545 | PFS | HR | Random | NA | 0.98(0.90-1.06) | 3.2(0.401) | 1.01(0.9-1.13) | N | DL | 0.568 | 0.977[0.903, 1.058] | 2.567 | [0.872, 1.095] | 0.0369 | 0.087 | ns |
| Zhang, Lilong 2022 | Probiotics | Anti-PD-(L)1; Anti-CTLA-4 | Multiple | 4 | 96/569 | OS | HR | Random | NA | 0.53(0.34-0.81) | 22.8(0.274) | 0.61(0.48-0.76) | N | HKSJ | 0.0574 | 0.525[0.265, 1.038] | 22.544 | [0.265, 1.038] | 0.518 | 0.156 | ns |
| Zhang, Lilong 2022 | Probiotics | Anti-PD-(L)1; Anti-CTLA-4 | Multiple | 5 | 145/678 | PFS | HR | Random | NA | 0.59(0.33-1.05) | 82.6(<0.0001) | 1.31(0.46-3.68) | N | DL | 0.0706 | 0.587[0.329, 1.046] | 82.516 | [0.078, 4.418] | 0.836 | 0.221 | ns |
| Zhang, Lilong 2022 | Probiotics | Anti-PD-(L)1; Anti-CTLA-4 | Multiple | 4 | 104/637 | ORR | OR | Random | NA | 2.83(1.58-5.08) | 37.4(0.188) | 0.89(0.24-3.29) | N | HKSJ | 0.0442 | 2.829[1.052, 7.609] | 37.278 | [1.052, 7.609] | 0.534 | 0.632 | IV |
| Zhang, Lilong 2022 | Probiotics | Anti-PD-(L)1; Anti-CTLA-4 | Multiple | 4 | 139/460 | DCR | OR | Random | NA | 1.87(0.89-3.92) | 69.1(0.021) | 2.78(1.79-4.35) | N | HKSJ | 0.197 | 1.867[0.56, 6.224] | 69.443 | [0.56, 6.224] | 0.851 | 0.381 | ns |
| Wan 2022 | Probiotics | Anti-PD-(L)1; Anti-CTLA-4 | NSCLC | 5 | 103/928 | OS | HR | Random | NA | 0.50(0.30-0.85) | 64.8(0.023) | 0.83(0.5-1.38) | N | DL | 0.00952 | 0.504[0.3, 0.846] | 65.186 | [0.099, 2.555] | 0.498 | 0.0308 | IV |
| Wan 2022 | Probiotics | Anti-PD-(L)1; Anti-CTLA-4 | NSCLC | 4 | 89/642 | PFS | HR | Random | NA | 0.51(0.42-0.61) | 15(0.317) | 0.5(0.41-0.61) | N | HKSJ | 0.0136 | 0.514[0.343, 0.771] | 12.09 | [0.343, 0.771] | 0.569 | 0.598 | IV |
| Zhang, Lilong 2022 | Probiotics | Anti-PD-(L)1; Anti-CTLA-4 | NSCLC | 3 | 85/627 | ORR | OR | Random | NA | 2.55(1.28-5.09) | 54.5(0.111) | 0.89(0.24-3.29) | N | HKSJ | 0.134 | 2.551[0.494, 13.181] | 54.438 | [0.494, 13.181] | 0.108 | 0.496 | ns |
| Zhang, Lilong 2022 | Probiotics | Anti-PD-(L)1; Anti-CTLA-4 | RCC | 1 | 19/10 | OS | HR | Random | NA | 0.31(0.05-1.89) | NA | 0.31(0.05-1.89) | Y | HKSJ | 0.204 | 0.31[0.051, 1.89] | only 1 study | [0.051, 1.89] | < 3 studies | NA | ns |
| Zhang, Lilong 2022 | Probiotics | Anti-PD-(L)1; Anti-CTLA-4 | RCC | 1 | 19/10 | ORR | OR | Random | NA | 5.50(0.91-33.18) | NA | 5.50(0.91-33.18) | Y | HKSJ | 0.063 | 5.5[0.912, 33.18] | only 1 study | [0.912, 33.18] | < 3 studies | NA | ns |
| Ju 2022 | Opioids | Anti-PD-(L)1; Anti-CTLA-4 | Multiple | 7 | 620/2070 | OS | HR | Random | <0.0001 | 1.75(1.32-2.31) | 81(<0.0001) | 1.53(1.11-2.11) | N | DL | 0.0000954 | 1.748[1.321, 2.315] | 80.441 | [0.693, 4.409] | 0.9 | 0.435 | IV |
| Ju 2022 | Opioids | Anti-PD-(L)1; Anti-CTLA-4 | Multiple | 5 | 333/1685 | PFS | HR | Fixed | <0.0002 | 1.61(1.41-1.83) | 0(0.63) | 1.71(1.28-2.28) | Y | DL | 6.34E-13 | 1.611[1.415, 1.834] | 0 | [1.304, 1.989] | 0.014 | 0.0684 | IV |
| Mao 2022 | Opioids | Anti-PD-(L)1; Anti-CTLA-4 | Multiple | 5 | 435/1518 | ORR | OR | Fixed | NA | 0.49(0.37-0.65) | 32(0.210) | 0.76(0.43-1.33) | N | DL | 0.000153 | 0.491[0.34, 0.71] | 32.376 | [0.188, 1.28] | 0.32 | 0.33 | IV |
| Mao 2022 | NSAIDs | Anti-PD-(L)1; Anti-CTLA-4 | Multiple | 8 | 514/1835 | OS | HR | Fixed | NA | 0.90(0.71-1.14) | 54(0.030) | 1.30(0.92-1.83) | N | DL | 0.423 | 0.91[0.723, 1.146] | 52.055 | [0.487, 1.699] | 0.0164 | 0.379 | ns |
| Mao 2022 | NSAIDs | Anti-PD-(L)1; Anti-CTLA-4 | Multiple | 5 | 322/1600 | PFS | HR | Fixed | NA | 0.90(0.77-1.06) | 21(0.280) | 1.07(0.78-1.74) | Y | DL | 0.207 | 0.883[0.728, 1.071] | 14.829 | [0.583, 1.338] | 0.892 | 0.253 | ns |
| Mao 2022 | NSAIDs | Anti-PD-(L)1; Anti-CTLA-4 | Multiple | 8 | 462/1760 | ORR | OR | Random | NA | 1.40(0.84-2.32) | 75(<0.001) | 0.61(0.33-1.11) | N | DL | 0.193 | 1.398[0.845, 2.316] | 74.766 | [0.277, 7.061] | 0.0366 | 0.35 | ns |

^*^ Review reported incomplete data on sample size.

**Abbreviation:** ATB, antibiotics; CM, concomitant medications; CI, confidence interval; DCR, disease control rate; DL, DerSimonian-Laird method; ES, effect size; ESCC, esophageal squamous cell carcinoma; H2RAs, Histamine 2 receptor antagonists; HCC, hepatocellular carcinoma; HR, hazard ratio; HKSJ, Hartung-Knapp-Sidik-Jonkman method; IC-PFS, intracranial progression-free survival; irAEs, immune-related adverse events; MA, meta-analysis; N, No; Y, Yes; NA, not available; NSAIDs, nonsteroidal anti-inflammatory agents; NSCLC, non-small cell lung cancer; ORR, objective response rate; OR, odds ratio; OS, overall survival; PD, progression disease; PI, prediction Interval; PPIs, proton pump inhibitors; PFS, progression-free survival; RA, re-analysis; RCC, renal cell carcinoma; RR, response to treatment rate; SRS, stereotactic radiotherapy; TES, test of excess significance; UC, urothelial carcinoma; Ⅱ, highly suggestive evidence (class Ⅱ); Ⅲ, suggestive evidence (class Ⅲ); Ⅳ, weak evidence (class Ⅳ); ns, non-significant (class ns).
