# Supplementary material for: Impact of concomitant medications on the efficacy of immune checkpoint inhibitors: an umbrella review
Source: Front Immunol. 2023 Sep 29;14:1218386. doi: 10.3389/fimmu.2023.1218386 (PMC10570520; doi:10.3389/fimmu.2023.1218386)
Supplement: Supplementary file 1 [file DataSheet_1.zip › Supplementary_Materials/Table S8.docx]

**Table S8.** Sensitivity analysis of the impact of concomitant medications on ICIs efficacy

| **CM** | **Outcome** | **Cancer type** | **Sensitivity analysis** | **No. of primary studies** | **No. of patients** | **Metric** | **RA p-value** | **RA ES (95% CI)** | **RA I2** | **95% PI** | **Egger p-Value** | **TES p-value** | **Level of evidence** |
| --- | --- | --- | --- | --- | --- | --- | --- | --- | --- | --- | --- | --- | --- |
| PPIs (any exposure window) | OS | Multiple | full analysis | 35 | 6969/7997* | HR | 2.17E-08 | 1.31[1.192, 1.44] | 72.003 | [0.85, 2.017] | 0.0697 | 0.0000571 | III |
|  |  |  | minus small-sized studies | 26 | 6714/7683* | HR | 4.20E-08 | 1.309 [1.189, 1.441] | 75.452 | [0.857, 2] | 3.48E-02 | 0.000119 | III |
|  | PFS |  | full analysis | 26 | 3145/4417* | HR | 0.00000194 | 1.305[1.169, 1.456] | 62.982 | [0.852, 1.998] | 0.67 | 0.101 | III |
|  |  |  | minus small-sized studies | 17 | 2890/4103* | HR | 0.000000801 | 1.301[1.172, 1.445] | 60.031 | [0.91, 1.86] | 0.387 | 0.0102 | II |
| PPIs [-60, NA] | OS | Multiple | full analysis | 20 | 3153/4689 | HR | 2.78E-08 | 1.355[1.217, 1.508] | 57.971 | [0.94, 1.952] | 0.754 | 0.031 | II |
|  |  |  | minus small-sized studies | 17 | 3085/4591 | HR | 9.28E-08 | 1.35[1.209, 1.507] | 62.496 | [0.926, 1.967] | 0.811 | 0.073 | II |
|  | PFS |  | full analysis | 17 | 1886/3090* | HR | 0.000000201 | 1.332[1.196, 1.484] | 48.019 | [0.959, 1.85] | 0.732 | 0.307 | II |
|  |  |  | minus small-sized studies | 14 | 1818/2992 | HR | 0.000000271 | 1.353[1.206, 1.518] | 55.603 | [0.946, 1.936] | 0.835 | 0.238 | II |
| PPIs (any exposure window) | OS | NSCLC | full analysis | 16 | 4506/4404 | HR | 0.0000885 | 1.331[1.154, 1.535] | 74.62 | [0.825, 2.148] | 0.0371 | 0.00609 | III |
|  |  |  | minus small-sized studies | 12 | 4350/4283 | HR | 0.00135 | 1.27[1.097, 1.469] | 75.452 | [0.788, 2.045] | 0.161 | 0.114 | IV |
|  | PFS |  | full analysis | 13 | 1361/1969 | HR | 0.0000172 | 1.326[1.166, 1.508] | 43.18 | [0.946, 1.858] | 0.52 | 0.61 | III |
|  |  |  | minus small-sized studies | 9 | 1205/1848 | HR | 6.47E-09 | 1.28[1.178, 1.391] | 0 | [1.158, 1.415] | 0.0602 | 0.696 | II |
| ATB [-60,0] | OS | NSCLC | full analysis | 13 | 1318/4926 | HR | 1.99E-09 | 2.277[1.74, 2.98] | 79.811 | [0.944, 5.494] | 0.000583 | 1.41E-10 | II |
|  |  |  | minus small-sized studies | 9 | 1252/4672 | HR | 2.90E-07 | 2.252[1.652, 3.072] | 84.845 | [0.844, 6.013] | 0.00372 | 2.71E-10 | II |
| ATB (any exposure window) | OS | Multiple | full analysis | 38 | 3019/9287* | HR | 1.17E-15 | 1.198[1.146, 1.253] | 83.962 | [0.956, 1.503] | 0.000000401 | 0 | III |
|  |  |  | minus small-sized studies | 29 | 2899/8919* | HR | 8.97E-12 | 1.168[1.117, 1.221] | 84.377 | [-0.031, 0.202] | 0.0000499 | 0 | III |
|  | PFS |  | full analysis | 31 | 1663/4454* | HR | 1.81E-08 | 1.174[1.11, 1.242] | 73.038 | [0.877, 1.572] | 0.00808 | 5.54E-11 | II |
|  |  |  | minus small-sized studies | 21 | 1513/4028 | HR | 2.03E-04 | 1.115[1.053, 1.182] | 72.29 | [0.856, 1.453] | 0.316 | 0.000209 | III |
| ATB | OS | NSCLC | full analysis | 27 | 1232/4492* | HR | 0.00000113 | 1.255[1.146, 1.376] | 78.405 | [0.814, 1.937] | 0.0264 | 0.0000441 | III |
|  |  |  | minus small-sized studies | 22 | 1171/4317* | HR | 1.36E-04 | 1.207[1.096, 1.33] | 80.044 | [-0.134, 0.341] | 0.158 | 0.00777 | III |
| Steroids (overall) | OS | Multiple | full analysis | 36 | 1976/6722* | HR | 6.26E-10 | 1.513[1.327, 1.725] | 70.646 | [0.839, 2.727] | 0.15 | 0.000281 | II |
|  |  |  | minus small-sized studies | 30 | 1895/6462 | HR | 1.36E-08 | 1.47[1.287, 1.679] | 72.445 | [0.829, 2.608] | 0.299 | 0.000826 | II |
|  | PFS |  | full analysis | 24 | 773/4423* | HR | 0.0000233 | 1.505[1.245, 1.819] | 76.373 | [0.669, 3.386] | 0.72 | 0.00000009 | III |
|  |  |  | minus small-sized studies | 21 | 731/4260* | HR | 4.53E-04 | 1.42[1.167, 1.727] | 77.818 | [0.631, 3.194] | 0.833 | 0.000131 | III |
|  | OS | NSCLC | full analysis | 11 | 1009/3433* | HR | 1.01E-10 | 1.816[1.516, 2.177] | 62.489 | [1.054, 3.13] | 0.0498 | 0.0000252 | II |
|  |  |  | minus small-sized studies | NC | NC | NC | NC | NC | NC | NC | NC | NC | NC |
| Steroids (cancer indication) | OS | Multiple | full analysis | 12 | 584/2559* | HR | 7.98E-11 | 1.937[1.587, 2.364] | 51.542 | [1.133, 3.311] | 0.526 | 0.0693 | II |
|  |  |  | minus small-sized studies | 9 | 547/2426 | HR | 1.70E-11 | 1.885[1.567, 2.268] | 45.823 | [1.186, 2.999] | 0.596 | 0.0479 | II |
|  | PFS |  | full analysis | 8 | 375/2172* | HR | 0.00000206 | 1.737[1.383, 2.183] | 64.363 | [0.922, 3.274] | 0.0297 | 0.00000339 | III |
|  |  |  | minus small-sized studies | 7 | 365/2115* | HR | 9.37E-06 | 1.658[1.326, 2.073] | 63.967 | [0.886, 3.101] | 0.0763 | 0.000169 | III |

* Review reported incomplete data on sample size.

**Abbreviation:** CM, concomitant medications; CI, confidence interval; ES, effect size; HR, hazard ratio; NSCLC, non-small cell lung cancer; NC, not computable because no individual studies with a sample size of less than 100 were included in the meta-analysis; OS, overall survival; PFS, progression-free survival; PI, prediction Interval; RA, re-analysis; TES, test of excess significance; Ⅱ, highly suggestive evidence (class Ⅱ); Ⅲ, suggestive evidence (class Ⅲ); Ⅳ, weak evidence (class Ⅳ); ns, non-significant (class ns).
