# Supplementary material for: Impact of concomitant medications on the efficacy of immune checkpoint inhibitors: an umbrella review
Source: Front Immunol. 2023 Sep 29;14:1218386. doi: 10.3389/fimmu.2023.1218386 (PMC10570520; doi:10.3389/fimmu.2023.1218386)
Supplement: Supplementary file 1 [file DataSheet_1.zip › Supplementary_Materials/Table S4.docx]

**Table S4.** Citation matrices for meta-analyses with overlapping associations

**1. OS/PFS of PPIs + ICIs, multiple cancer**

| **Meta-analysis** | Li, Chao 2020 ^1^ | Li, Manyu 2020 ^2^ | Liu 2022 ^3^ | Qin 2021 ^4^ | Chen 2022 ^5^ | Wu 2022 ^6^ | Deng 2022 ^7^ |
| --- | --- | --- | --- | --- | --- | --- | --- |
| **Overlapping associations** | OS/PFS of PPIs + ICIs, multiple cancer | | | | | | |
| **Individual study** |  | | | | | | |
| Afzal 2019 | 1 | 1 | 1 | 0 | 1 | 0 | 1 |
| Araujo 2021 | 0 | 0 | 1 | 0 | 1 | 0 | 1 |
| Baek 2022 | 0 | 0 | 0 | 0 | 1 | 0 | 1 |
| Buti 2021 | 0 | 0 | 1 | 1 | 1 | 0 | 1 |
| Castro 2021 | 0 | 0 | 0 | 0 | 1 | 0 | 0 |
| Chalabi 2020 | 1 | 1 | 1 | 1 | 1 | 0 | 1 |
| Conde-Estevez 2020 | 0 | 0 | 0 | 0 | 1 | 0 | 0 |
| Cortellini 2020 | 0 | 0 | 1 | 1 | 1 | 1 | 1 |
| Cortellini 2021 | 0 | 0 | 0 | 0 | 1 | 0 | 1 |
| Failing 2016 | 1 | 1 | 1 | 0 | 1 | 0 | 1 |
| Gaucher 2021 | 0 | 0 | 0 | 0 | 1 | 0 | 0 |
| Giorgdan 2021 | 0 | 0 | 0 | 0 | 1 | 0 | 1 |
| Hakozaki 2019 | 0 | 1 | 1 | 1 | 0 | 0 | 1 |
| Hopkins 2020 | 0 | 0 | 1 | 1 | 1 | 0 | 1 |
| Hopkins 2022 | 0 | 0 | 0 | 0 | 1 | 1 | 0 |
| Hopkins 2021 | 0 | 0 | 0 | 0 | 0 | 1 | 1 |
| Hossain 2020 | 0 | 0 | 0 | 0 | 1 | 0 | 1 |
| Husain 2021 | 0 | 0 | 1 | 0 | 1 | 0 | 1 |
| Iglesias 2020 | 1 | 1 | 1 | 1 | 1 | 0 | 1 |
| Jun 2021 | 0 | 0 | 1 | 0 | 1 | 0 | 1 |
| Kostine 2021 | 0 | 0 | 0 | 0 | 1 | 0 | 1 |
| Kulkarni 2019 | 0 | 0 | 0 | 0 | 1 | 0 | 0 |
| Miura 2021 | 0 | 0 | 1 | 0 | 1 | 0 | 0 |
| Mollica 2021 | 0 | 0 | 0 | 0 | 1 | 0 | 1 |
| Nguyen 2019 | 0 | 0 | 0 | 0 | 1 | 0 | 0 |
| Peng 2021 | 0 | 0 | 1 | 0 | 1 | 0 | 1 |
| Routy 2017 | 0 | 0 | 0 | 0 | 1 | 0 | 0 |
| Ruiz 2021 | 0 | 0 | 1 | 0 | 1 | 1 | 1 |
| Spakowicz 2020 | 0 | 0 | 0 | 0 | 1 | 0 | 0 |
| Stein 2020 | 0 | 0 | 0 | 0 | 1 | 0 | 0 |
| Stokes 2021 | 0 | 0 | 1 | 0 | 1 | 0 | 1 |
| Svaton 2020 | 0 | 1 | 1 |  | 1 | 1 | 1 |
| Takada 2022 | 0 | 0 | 0 | 0 | 1 | 0 | 1 |
| Zhao 2019 | 1 | 1 | 1 | 1 | 1 | 1 | 1 |
| Fukuokaya 2022 | 0 | 0 | 0 | 0 | 0 | 0 | 1 |
| Tomisaki 2022 | 0 | 0 | 0 | 0 | 0 | 0 | 1 |
| **Decision to retain** | ✘ | ✘ | ✘ | ✘ | **✓** | ✘ | ✘ |
| **Grand Total (N)** | 100 |  | | | | | |
| **Rows (r)** | 36 |  |  |  |  |  |  |
| **Columns (c)** | 7 |  |  |  |  |  |  |
| **CCA** | 29.6% |  |  |  |  |  |  |

**2. OS/PFS of PPIs + ICIs, melanoma**

| **Meta-analysis** | Li, Manyu 2020 ^2^ | Chen 2022 ^5^ |
| --- | --- | --- |
| **Overlapping associations** | OS/PFS of PPIs+ICIs, Melanoma | |
| **Individual study** |  | |
| Afzal 2019 | 1 | 1 |
| Failing 2016 | 1 | 1 |
| Stein 2020 | 0 | 1 |
| Nguyen 2019 | 0 | 1 |
| Kostine 2021 | 0 | 1 |
| **Decision to retain** | ✘ | **✓** |
| **Grand Total (N)** | 7 |  |
| **Rows (r)** | 5 |  |
| **Columns (c)** | 2 |  |
| **CCA** | 40% |  |

**3. OS/PFS of PPIs + ICIs, NSCLC**

| **Meta-analysis** | Li, Chao 2020 ^1^ | Li, Manyu 2020 ^2^ | Wei 2022 ^8^ | Hu 2022 ^9^ | Chen 2022 ^5^ | Dar 2022 ^10^ |
| --- | --- | --- | --- | --- | --- | --- |
| **Overlapping associations** | OS/PFS of PPIs+ICIs, NSCLC | | | | | |
| **Individual study** |  | | | | | |
| Baek 2022 | 0 | 0 | 0 | 0 | 1 | 0 |
| Castro 2021 | 0 | 0 | 0 | 0 | 1 | 0 |
| Chalabi 2020 | 1 | 1 | 1 | 1 | 1 | 1 |
| Conde 2020 | 0 | 0 | 0 | 0 | 1 | 0 |
| Cortellini 2021 | 0 | 0 | 1 | 1 | 1 | 0 |
| Giorgdan 2021 | 0 | 0 | 0 | 1 | 0 | 0 |
| Hakozaki 2019 | 0 | 1 | 1 | 1 | 0 | 0 |
| Hopkins 2022 | 0 | 0 | 0 | 1 | 1 | 1 |
| Hossain 2020 | 0 | 0 | 0 | 0 | 1 | 0 |
| Husain 2021 | 0 | 0 | 0 | 1 | 0 | 0 |
| Kostine 2021 | 0 | 0 | 0 | 0 | 1 | 0 |
| Kulkarni 2019 | 0 | 0 | 0 | 0 | 1 | 0 |
| Miura 2021 | 0 | 0 | 0 | 1 | 1 | 0 |
| Peng 2022 | 0 | 0 | 0 | 0 | 1 | 1 |
| Routy 2017 | 0 | 0 | 0 | 0 | 1 | 0 |
| Stokes 2021 | 0 | 0 | 0 | 1 | 1 | 0 |
| Svaton 2020 | 0 | 1 | 1 | 1 | 1 | 0 |
| Takada 2022 | 0 | 0 | 0 | 0 | 1 | 0 |
| Zhao 2019 | 1 | 1 | 1 | 1 | 1 | 1 |
| **Decision to retain** | ✘ | ✘ | ✘ | ✘ | **✓** | ✘ |
| **Grand Total (N)** | 41 |  | | | | |
| **Rows (r)** | 19 |  |  |  |  |  |
| **Columns (c)** | 6 |  |  |  |  |  |
| **CCA** | 23.2% |  |  |  |  |  |

**4. OS/PFS of PPIs + ICIs, UC**

| **Meta-analysis** | Chen 2022 ^5^ | Rizzo 2022 ^11^ | Zhang 2022 ^12^ |
| --- | --- | --- | --- |
| **Overlapping associations** | OS/PFS of PPIs+ICIs, UC | | |
| **Individual study** |  | | |
| Hopkins 2020 | 1 | 1 | 1 |
| Lida 2021 | 1 | 0 | 0 |
| Ruiz 2021 | 1 | 1 | 1 |
| Routy 2017 | 1 | 0 | 0 |
| Okuyama 2022 | 0 | 0 | 1 |
| Tomisaki 2022 | 0 | 0 | 1 |
| Fukuokaya 2022 | 0 | 0 | 1 |
| Kunimistu 2022 | 0 | 0 | 1 |
| **Decision to retain** | ✘ | ✘ | **✓** |
| **Grand Total (N)** | 12 |  | |
| **Rows (r)** | 8 |  |  |
| **Columns (c)** | 3 |  |  |
| **CCA** | 25% |  |  |

**5.** **ORR of ATB + ICIs, multiple cancer**

| **Meta-analysis** | Wu 2021 ^13^ | Xu 2020 ^14^ | Yu 2021 ^15^ |
| --- | --- | --- | --- |
| **Overlapping associations** | ORR of PPIs+ICIs, multiple cancer | | |
| **Individusl study** |  | | |
| Agarwal 2019 | 0 | 0 | 1 |
| Ahmed 2018 | 1 | 1 | 1 |
| Derosa 2020 | 1 | 0 | 0 |
| Elkrief 2019 | 1 | 1 | 1 |
| Galli 2019 | 1 | 0 | 1 |
| Iglesias 2020 | 1 | 0 | 0 |
| Kaderbhai 2017 | 1 | 1 | 1 |
| Kapoor 2019 | 1 | 1 | 0 |
| Kim 1 2019 | 1 | 0 | 0 |
| Krief 2019 | 0 | 0 | 1 |
| Kulkarni 2019 | 1 | 0 | 1 |
| Lalani 2020 | 1 | 0 | 1 |
| Pinato 2019 | 0 | 0 | 1 |
| Ruiz 2020 | 1 | 0 | 0 |
| Schett 2020 | 1 | 1 | 0 |
| Thompson 2017 | 1 | 1 | 1 |
| Ueda 2019 | 1 | 0 | 0 |
| Weinstock 2019 | 0 | 0 | 1 |
| Zhao 2019 | 1 | 1 | 1 |
| **Decision to retain** | **✓** | ✘ | ✘ |
| **Grand Total (N)** | 34 |  | |
| **Rows (r)** | 19 |  |  |
| **Columns (c)** | 3 |  |  |
| **CCA** | 39.55% |  |  |

**6.** **OS/PFS of ATB** **before ICIs initiation, multiple cancer**

| **Meta-analysis** | Wu 2021 ^13^ | Huang, Xuanzhang 2019 ^16^ | Huang, Litang 2021 ^17^ | Wilson 2019 ^18^ | Huang, Litang 2021 ^17^ | Zhou 2022 ^19^ | Huang, Litang 2021 ^17^ |
| --- | --- | --- | --- | --- | --- | --- | --- |
| **Overlapping associations** | before ICIs initiation | before ICIs initiation | [-30,0] | [-42,0] | [-60,0] | [-60,0] | [-90,0] |
| **Individual study** |  | | | | | | |
| Agarwal 2019 | 0 | 1 | 0 | 0 | 0 | 0 | 0 |
| Barron 2019 | 0 | 0 | 1 | 0 | 0 | 0 | 0 |
| Cren 2020 | 0 | 0 | 0 | 0 | 0 | 1 | 0 |
| Derosa RCC 2020 | 1 | 0 | 0 | 0 | 0 | 0 | 0 |
| Derosa RCC 2018 | 1 | 1 | 1 | 1 | 1 | 0 | 0 |
| Derosa NSCLC 2018 | 1 | 0 | 1 | 1 | 1 | 0 | 0 |
| Elkrief 2019 | 1 | 1 | 1 | 1 | 0 | 0 | 0 |
| Facchinetti 2020 | 0 | 0 | 0 | 0 | 1 | 0 | 0 |
| Greally 2019 | 1 | 0 | 0 | 0 | 0 | 0 | 0 |
| Hakozaki 2019 | 0 | 1 | 1 | 0 | 0 | 0 | 0 |
| Hakozaki 2018 | 0 | 0 | 1 | 0 | 0 | 0 | 0 |
| Hogue 2019 | 1 | 0 | 0 | 0 | 0 | 0 | 1 |
| Kapoor 2020 | 0 | 0 | 1 | 0 | 0 | 0 | 0 |
| Khan 2020 | 0 | 0 | 0 | 0 | 1 | 0 | 0 |
| Kim 2019 | 1 | 1 | 1 | 0 | 1 | 1 | 0 |
| Kulkarni 2018 | 0 | 0 | 0 | 0 | 0 | 0 | 1 |
| Ouaknine 2019 | 0 | 0 | 0 | 0 | 0 | 1 | 0 |
| Pinato 2019 | 0 | 1 | 1 | 1 | 0 | 0 | 0 |
| Quentin 2021 | 0 | 0 | 0 | 0 | 0 | 1 | 0 |
| Schett 2020 | 1 | 0 | 0 | 0 | 1 | 1 | 0 |
| Sen 2018 | 1 | 1 | 1 | 1 | 0 | 0 | 0 |
| Swami 2020 | 0 | 0 | 0 | 0 | 1 | 0 | 0 |
| Thompson 2017 | 1 | 1 | 0 | 1 | 0 | 0 | 0 |
| Tinsley 2018 | 0 | 1 | 0 | 0 | 0 | 0 | 0 |
| Ueda 2019 | 1 | 0 | 0 | 0 | 0 | 0 | 0 |
| Zhao 2019 | 0 | 0 | 0 | 1 | 0 | 0 | 0 |
| **Decision to retain** | **✓** | ✘ | ✘ | ✘ | ✘ | ✘ | ✘ |
| **Grand Total (N)** | 51 |  | | | | | |
| **Rows (r)** | 26 |  |  |  |  |  |  |
| **Columns (c)** | 7 |  |  |  |  |  |  |
| **CCA** | 16.0% |  |  |  |  |  |  |

**7. OS/PFS of ATB + ICIs, multiple cancer, any exposure window**

| **Meta-analysis** | Wilson 2019 ^18^ | Huang, Xuanzhang 2019 ^16^ | Petrelli 2020 ^20^ | Tsikala 2021 ^21^ | Xu 2020 ^14^ | Yang 2020 ^22^ | Yu 2021 ^15^ | Wu 2021 ^13^ | Jiang 2022 ^23^ | Zhou 2022 ^19^ |
| --- | --- | --- | --- | --- | --- | --- | --- | --- | --- | --- |
| **Overlapping associations** | OS/PFS of ATB+ICIs, multiple cancer, any exposure window | | | | | | | | | |
| **Individual study** |  | | | | | | | | | |
| Abu-Sbeih 2019 | 0 | 0 | 1 | 1 | 0 | 0 | 0 | 1 | 0 | 0 |
| Agarwal 2019 | 0 | 1 | 0 | 0 | 0 | 1 | 1 | 0 | 0 | 0 |
| Ahmed 2018 | 1 | 1 | 1 | 0 | 1 | 1 | 1 | 1 | 0 | 1 |
| Bagley melanoma 2019 | 0 | 0 | 0 | 1 | 0 | 0 | 0 | 1 | 0 | 0 |
| Bagley NSCLC 2019 | 0 | 0 | 0 | 1 | 0 | 0 | 0 | 1 | 0 | 0 |
| Barron 2019 | 0 | 0 | 0 | 0 | 0 | 0 | 0 | 0 | 0 | 1 |
| Castello 2021 | 0 | 0 | 0 | 0 | 0 | 0 | 0 | 0 | 0 | 1 |
| Chalabi 2018 | 0 | 0 | 0 | 0 | 1 | 1 | 1 | 1 | 0 | 0 |
| Chambers 2021 | 0 | 0 | 0 | 0 | 0 | 0 | 0 | 0 | 1 | 1 |
| Cortellini 2021 | 0 | 0 | 0 | 0 | 0 | 0 | 0 | 0 | 1 | 1 |
| Cren 2020 | 0 | 0 | 0 | 0 | 0 | 0 | 0 | 0 | 0 | 0 |
| Derosa NSCLC 2018 | 1 | 1 | 1 | 1 | 1 | 1 | 1 | 1 | 1 | 1 |
| Derosa RCC 2018 | 1 | 1 | 0 | 1 | 1 | 1 | 1 | 1 | 1 | 0 |
| Do 2018 | 1 | 1 | 0 | 0 | 0 | 1 | 1 | 1 | 0 | 0 |
| Elkrief 2019 | 1 | 1 | 1 | 0 | 1 | 1 | 1 | 1 | 1 | 1 |
| Facchinetti 2020 | 0 | 0 | 0 | 1 | 0 | 0 | 0 | 0 | 0 | 1 |
| Fessas 2021 | 0 | 0 | 0 | 0 | 0 | 0 | 0 | 0 | 0 | 1 |
| Galli 2019 | 0 | 0 | 1 | 0 | 1 | 1 | 0 | 1 | 0 | 1 |
| Gaucher 2021 | 0 | 0 | 0 | 0 | 0 | 0 | 0 | 0 | 0 | 0 |
| Geum 2021 | 0 | 0 | 0 | 0 | 0 | 0 | 0 | 0 | 0 | 0 |
| Greally 2019 | 0 | 0 | 0 | 0 | 0 | 0 | 1 | 1 | 0 | 0 |
| Guo 2019 | 0 | 0 | 1 | 1 | 0 | 1 | 1 | 1 | 0 | 0 |
| Guven 2021 | 0 | 0 | 0 | 0 | 0 | 0 | 0 | 0 | 0 | 1 |
| Hakozaki 2019 | 1 | 1 | 1 | 1 | 1 | 1 | 0 | 1 | 0 | 1 |
| Hamada 2021 | 0 | 0 | 0 | 0 | 0 | 0 | 0 | 0 | 1 | 1 |
| Hemadri 2019 | 1 | 0 | 0 | 0 | 0 | 0 | 0 | 0 | 0 | 1 |
| Hogue 2019 | 0 | 0 | 0 | 0 | 0 | 0 | 0 | 1 | 0 | 1 |
| Hopkins 2020 | 0 | 0 | 0 | 0 | 0 | 0 | 0 | 1 | 0 | 0 |
| Huemer 2019 | 1 | 1 | 1 | 0 | 1 | 1 | 1 | 1 | 0 | 0 |
| Iglesias 2020 | 0 | 0 | 0 | 1 | 0 | 1 | 0 | 1 | 0 | 1 |
| Kapoor 2020 | 0 | 0 | 0 | 0 | 0 | 0 | 0 | 1 | 0 | 1 |
| Kim 2018 | 0 | 1 | 0 | 1 | 1 | 1 | 1 | 1 | 1 | 0 |
| Kim 2021 | 0 | 0 | 0 | 0 | 0 | 0 | 0 | 0 | 0 | 1 |
| Kulkarni 2019 | 1 | 0 | 0 | 0 | 0 | 1 | 1 | 1 | 0 | 0 |
| Lalani 2018 | 1 | 1 | 0 | 1 | 0 | 1 | 0 | 1 | 1 | 0 |
| Lu Ms 2020 | 0 | 0 | 0 | 0 | 0 | 0 | 0 | 0 | 0 | 1 |
| Masini 2019 | 1 | 0 | 0 | 0 | 0 | 1 | 1 | 1 | 0 | 0 |
| Mielgo Rubio 2018 | 1 | 0 | 0 | 0 | 0 | 1 | 0 | 1 | 0 | 0 |
| Mohiuddin 2020 | 0 | 0 | 0 | 1 | 0 | 0 | 0 | 1 | 1 | 1 |
| Nyein 2022 | 0 | 0 | 0 | 0 | 0 | 0 | 0 | 0 | 0 | 0 |
| Ochi 2021 | 0 | 0 | 0 | 0 | 0 | 0 | 0 | 0 | 0 | 0 |
| Ouaknine 2019 | 0 | 0 | 1 | 1 | 0 | 1 | 0 | 1 | 0 | 0 |
| Perez ruiz 2020 | 0 | 0 | 0 | 0 | 0 | 0 | 0 | 1 | 0 | 0 |
| Pinato 2018 | 1 | 1 | 1 | 1 | 1 | 1 | 1 | 1 | 0 | 0 |
| Pomej 2021 | 0 | 0 | 0 | 0 | 0 | 0 | 0 | 0 | 0 | 1 |
| Quentin 2021 | 0 | 0 | 0 | 0 | 0 | 0 | 0 | 0 | 0 | 0 |
| Riudavates 2019 | 0 | 0 | 0 | 0 | 0 | 0 | 0 | 1 | 0 | 0 |
| Rounis 2019 | 0 | 1 | 0 | 1 | 0 | 1 | 0 | 1 | 0 | 1 |
| Routy NSCLC 2018 | 1 | 1 | 0 | 1 | 1 | 1 | 1 | 1 | 0 | 1 |
| Routy UC 2018 | 1 | 1 | 0 | 0 | 1 | 1 | 0 | 1 | 0 | 1 |
| Rubio 2019 | 0 | 0 | 0 | 0 | 0 | 0 | 0 | 0 | 0 | 1 |
| Ruiz 2020 | 0 | 0 | 0 | 0 | 0 | 0 | 0 | 1 | 0 | 1 |
| Schett 2019 | 1 | 1 | 0 | 1 | 1 | 1 | 1 | 1 | 1 | 1 |
| Sen 2018 | 1 | 1 | 1 | 0 | 1 | 1 | 1 | 1 | 0 | 1 |
| Spakowicz 2020 | 0 | 0 | 0 | 1 | 0 | 0 | 0 | 1 | 0 | 1 |
| Svaton 2020 | 0 | 0 | 0 | 1 | 0 | 0 | 0 | 1 | 0 | 1 |
| Swami 2020 | 0 | 0 | 0 | 0 | 0 | 0 | 0 | 0 | 1 | 1 |
| Thompson 2018 | 1 | 1 | 0 | 1 | 1 | 1 | 0 | 1 | 0 | 1 |
| Tinsley 2019 | 1 | 1 | 1 | 1 | 0 | 1 | 1 | 1 | 1 | 1 |
| Ueda 2019 | 0 | 0 | 0 | 0 | 0 | 0 | 0 | 0 | 1 | 1 |
| Weinstock 2019 | 0 | 0 | 0 | 0 | 0 | 0 | 1 | 0 | 0 | 1 |
| Zhao 2019 | 1 | 1 | 1 | 1 | 1 | 1 | 1 | 1 | 0 | 1 |
| **Decision to retain** | ✘ | ✘ | ✘ | ✘ | ✘ | ✘ | ✘ | **✓** | ✘ | ✘ |
| **Grand Total (N)** | 225 |  | | | | | | | | |
| **Rows (r)** | 62 |  |  |  |  |  |  |  |  |  |
| **Columns (c)** | 10 |  |  |  |  |  |  |  |  |  |
| **CCA** | 29.2% |  |  |  |  |  |  |  |  |  |

**8. ORR of ATB + ICIs, NSCLC, any exposure window**

| **Meta-analysis** | Wu 2021 ^13^ | Crespin 2021 ^24^ |
| --- | --- | --- |
| **Overlapping associations** | ORR of ATB+ICIs, NSCLC, any exposure window | |
| **Individual study** |  | |
| Derosa 2018 | 1 | 1 |
| Galli 2019 | 1 | 1 |
| Kaderbhai 2017 | 1 | 0 |
| Kim 2019 | 1 | 0 |
| Kulkarni 2019 | 1 | 0 |
| Ruiz 2020 | 1 | 1 |
| Schett 2020 | 1 | 1 |
| Thompson 2017 | 1 | 1 |
| Zhao 2019 | 1 | 1 |
| Castello 2021 | 0 | 1 |
| Cortellini 2021 | 0 | 1 |
| Ouaknine 2019 | 0 | 1 |
| Rounis 2021 | 0 | 1 |
| **Decision to retain** | ✘ | **✓** |
| **Grand Total (N)** | 19 |  |
| **Rows (r)** | 13 |  |
| **Columns (c)** | 2 |  |
| **CCA** | 46.2% |  |

**9. OS/PFS of ATB, NSCLC, any exposure window**

| **Meta-analysis** | Lurienne 2020 ^25^ | Xu 2020 ^14^ | Wu 2021 ^13^ | Jiang 2022 ^23^ | Zhou 2022 ^19^ | Chen 2021 ^26^ |
| --- | --- | --- | --- | --- | --- | --- |
| **Overlapping associations** | OS/PFS of ATB, NSCLC, any exposure window | | | | | |
| **Individual study** |  | | | | | |
| Bagley 2019 | 1 | 0 | 1 | 0 | 0 | 0 |
| Barron 2019 | 1 | 0 | 0 | 0 | 0 | 0 |
| Castello 2021 | 0 | 0 | 0 | 0 | 1 | 0 |
| Chalabi 2018 | 1 | 1 | 1 | 0 | 1 | 0 |
| Cortellini 2021 | 0 | 0 | 0 | 1 | 0 | 0 |
| Derosa 2018 | 1 | 1 | 1 | 1 | 1 | 1 |
| Do 2019 | 1 | 0 | 1 | 0 | 0 | 0 |
| Fidler 2019 | 1 | 0 | 0 | 0 | 0 | 0 |
| Galli 2019 | 1 | 1 | 1 | 0 | 0 | 1 |
| Geum 2021 | 0 | 0 | 0 | 0 | 1 | 0 |
| Hakozaki 2019 | 1 | 1 | 1 | 0 | 1 | 1 |
| Hamada 2021 | 0 | 0 | 0 | 1 | 1 | 0 |
| Hogue 2019 | 0 | 0 | 1 | 0 | 0 | 0 |
| Huemer 2019 | 1 | 1 | 1 | 0 | 1 | 1 |
| Kim 2018 | 0 | 1 | 1 | 0 | 1 | 1 |
| Kulkarni 2019 | 1 | 0 | 1 | 0 | 1 | 0 |
| Kulkarni 2020 | 0 | 0 | 0 | 0 | 1 | 0 |
| Lu 2020 | 0 | 0 | 0 | 0 | 1 | 0 |
| Metges 2019 | 1 | 0 | 0 | 0 | 0 | 0 |
| Mielgo 2018 | 1 | 0 | 1 | 0 | 0 | 0 |
| Mielgo 2019 | 1 | 0 | 1 | 0 | 0 | 0 |
| Nyein 2022 | 0 | 0 | 0 | 0 | 1 | 0 |
| Ochi 2021 | 0 | 0 | 0 | 0 | 1 | 0 |
| Ouaknine 2018 | 1 | 0 | 1 | 0 | 1 | 1 |
| Pinato 2019 | 1 | 0 | 1 | 0 | 0 | 1 |
| Riudavets 2019 | 1 | 0 | 1 | 0 | 0 | 0 |
| Rounis 2019 | 1 | 0 | 1 | 0 | 0 | 1 |
| Routy 2018 | 1 | 1 | 1 | 0 | 0 | 1 |
| Rubio 2019 | 0 | 0 | 0 | 0 | 1 | 0 |
| Ruiz 2020 | 0 | 0 | 1 | 0 | 0 | 0 |
| Schett 2019 | 1 | 1 | 1 | 1 | 1 | 1 |
| Svaton 2020 | 0 | 0 | 1 | 0 | 0 | 0 |
| Thompson 2017 | 1 | 1 | 1 | 0 | 0 | 1 |
| Zhao 2019 | 1 | 1 | 1 | 0 | 1 | 1 |
| **Decision to retain** | ✘ | **✓** | ✘ | ✘ | ✘ | ✘ |
| **Grand Total (N)** | 86 |  | | | | |
| **Rows (r)** | 34 |  |  |  |  |  |
| **Columns (c)** | 6 |  |  |  |  |  |
| **CCA** | 30.6% |  |  |  |  |  |

**10. OS/PFS of ATB before ICIs initiation, NSCLC**

| **Meta-analysis** | Bandinelli 2020 ^27^ | Chen 2021 ^26^ | Chen 2021 ^26^ | Lurienne 2020 ^25^ | Crespin 2021 ^24^ |
| --- | --- | --- | --- | --- | --- |
| **Overlapping associations** | [-90,0] | [-60,0] | [-30,0] | [-90,0] | [-60,0] |
| **Individual study** |  | | | | |
| Clark 2020 | 1 | 0 | 0 | 0 | 1 |
| Derosa 2018 | 1 | 1 | 1 | 1 | 1 |
| Facchinetti 2020 | 1 | 0 | 0 | 0 | 1 |
| Fidler 2019 | 1 | 0 | 0 | 1 | 0 |
| Hakozaki 2019 | 1 | 0 | 0 | 1 | 1 |
| Kim 2019 | 1 | 1 | 0 | 0 | 1 |
| Pinato 2019 | 1 | 1 | 1 | 1 | 1 |
| Rounis 2019 | 0 | 1 | 1 | 0 | 0 |
| Routy 2018 | 0 | 1 | 0 | 0 | 0 |
| Schett 2019 | 1 | 1 | 0 | 1 | 1 |
| Tomita 2020 | 1 | 0 | 0 | 0 | 1 |
| Cortellini 2021 | 0 | 0 | 0 | 0 | 1 |
| Lu 2020 | 0 | 0 | 0 | 0 | 1 |
| Ruiz 2020 | 0 | 0 | 0 | 0 | 1 |
| Stokes 2021 | 0 | 0 | 0 | 0 | 1 |
| Thompson 2017 | 0 | 0 | 0 | 0 | 1 |
| Tomita 2020 | 0 | 0 | 0 | 0 | 1 |
| **Decision to retain** | ✘ | ✘ | ✘ | ✘ | **✓** |
| **Grand Total (N)** | 37 |  | | | |
| **Rows (r)** | 17 |  |  |  |  |
| **Columns (c)** | 5 |  |  |  |  |
| **CCA** | 29.4% |  |  |  |  |

**11. OS/PFS of ATB after ICIs initiation, NSCLC**

| **Meta-analysis** | Bandinelli 2020 ^27^ | Lurienne 2020 ^25^ | Chen 2021 ^26^ |
| --- | --- | --- | --- |
| **Overlapping associations** | [0,60] | [0,60] | [0,30] |
| **Individual study** | OS/PFS of ATB after ICIs initiation, NSCLC | | |
| Bagley 2019 | 1 | 1 | 0 |
| Barron 2019 | 1 | 1 | 0 |
| Thompson 2017 | 1 | 1 | 1 |
| Hakozaki 2019 | 0 | 0 | 1 |
| Schett 2019 | 0 | 0 | 1 |
| **Decision to retain** | ✘ | **✓** | ✘ |
| **Grand Total (N)** | 9 |  | |
| **Rows (r)** | 5 |  |  |
| **Columns (c)** | 3 |  |  |
| **CCA** | 40% |  |  |

**12. ORR of ATB + ICIs, RCC**

| **Meta-analysis** | Luo 2022 ^28^ | Wu 2021 ^13^ |
| --- | --- | --- |
| **Overlapping associations** | ORR of ATB+ICIs, RCC | |
| **Individual study** |  | |
| Derosa 2021 | 1 | 1 |
| Derosa 2018 | 0 | 1 |
| Guven 2021 | 1 | 0 |
| Lalani 2020 | 1 | 1 |
| Ueda 2019 | 0 | 1 |
| **Decision to retain** | ✘ | **✓** |
| **Grand Total (N)** | 7 |  |
| **Rows (r)** | 5 |  |
| **Columns (c)** | 2 |  |
| **CCA** | 40% |  |

**13. OS/PFS of ATB + ICIs, RCC**

| **Meta-analysis** | Zhou 2022 ^19^ | Tsikala 2021 ^21^ | Xu 2020 ^14^ | Wu 2021 ^6^ | Jiang 2022 ^23^ | Luo 2022 ^28^ |
| --- | --- | --- | --- | --- | --- | --- |
| **Overlapping associations** | OS/PFS of ATB+ICIs, RCC | | | | | |
| **Individual study** |  | | | | | |
| Derosa 2020 | 0 | 0 | 0 | 1 | 0 | 1 |
| Derosa RCC 2018 | 1 | 1 | 1 | 1 | 1 | 0 |
| Guven 2021 | 1 | 0 | 0 | 0 | 0 | 1 |
| Kato 2022 | 0 | 0 | 0 | 0 | 0 | 1 |
| Kulkarni RCC 2020 | 1 | 1 | 0 | 1 | 0 | 1 |
| Lalani 2020 | 0 | 0 | 0 | 1 | 1 | 1 |
| Routy RCC 2018 | 0 | 1 | 1 | 1 | 0 | 0 |
| Ueda RCC 2019 | 1 | 0 | 0 | 1 | 1 | 1 |
| Ueda 2019 | 0 | 0 | 0 | 1 | 0 | 0 |
| **Decision to retain** | ✘ | ✘ | ✘ | **✓** | ✘ | ✘ |
| **Grand Total (N)** | 25 |  | | | | |
| **Rows (r)** | 9 |  |  |  |  |  |
| **Columns (c)** | 6 |  |  |  |  |  |
| **CCA** | 35.6% |  |  |  |  |  |

**14. OS/PFS of ATB + ICIs, melanoma**

| **Meta-analysis** | Wu 2021 ^13^ | Jiang 2022 ^23^ | Zhou 2022 ^19^ |
| --- | --- | --- | --- |
| **Overlapping associations** | OS/PFS of ATB+ICIs, melanoma | | |
| **Individual study** |  | | |
| Elkrief 2019 | 1 | 1 | 1 |
| Pinato 2019 | 1 | 0 | 1 |
| Mohiuddin 2020 | 1 | 0 | 1 |
| Bagley 2019 | 1 | 0 | 0 |
| Swami 2020 | 0 | 1 | 0 |
| Cren 2020 | 0 | 0 | 1 |
| **Decision to retain** | **✓** | ✘ | ✘ |
| **Grand Total (N)** | 10 |  | |
| **Rows (r)** | 6 |  |  |
| **Columns (c)** | 3 |  |  |
| **CCA** | 33.3% |  |  |

**15. OS/PFS of steroids + ICIs, multiple cancer**

| **Meta-analysis** | Petrelli 2020 ^29^ | Wang 2021 ^30^ |
| --- | --- | --- |
| **Overlapping associations** | OS/PFS of steroids+ICIs, multiple cancer | |
| **Individual study** |  | |
| Acharya 2017 | 1 | 1 |
| Arbour 2018 | 1 | 1 |
| Buti 2020 | 0 | 1 |
| Chasset 2015 | 1 | 1 |
| Cortellini 2020 | 0 | 1 |
| De Giglio 2020 | 0 | 1 |
| Drakaki 2020 | 0 | 1 |
| Dumenil 2018 | 1 | 1 |
| Faje 2018 | 1 | 1 |
| Fuca 2018 | 1 | 1 |
| Gauci 2020 | 0 | 1 |
| Hendriks 2019 | 1 | 1 |
| Horvat 2015 | 1 | 1 |
| Johnson 2015 | 1 | 1 |
| Metro 2020 | 0 | 1 |
| Mountzios 2020 | 0 | 1 |
| Pinato 2020 | 0 | 1 |
| Ricciuti 2019 | 1 | 1 |
| Scott 2018 | 1 | 1 |
| Spakowicz 2020 | 0 | 1 |
| Sukari 2019 | 1 | 1 |
| Svaton 2020 | 0 | 1 |
| Thompson 2020 | 0 | 1 |
| Tozuka 2020 | 0 | 1 |
| Vitale 2020 | 0 | 1 |
| Weber 2009 | 1 | 1 |
| Zaragoza 2016 | 1 | 1 |
| **Decision to retain** | ✘ | **✓** |
| **Grand Total (N)** | 41 |  |
| **Rows (r)** | 27 |  |
| **Columns (c)** | 2 |  |
| **CCA** | 51.9% |  |

**16. OS/PFS of probiotics + ICIs, NSCLC**

| **Meta-analysis** | Zhang 2022 ^31^ | Wan 2022 ^32^ |
| --- | --- | --- |
| **Overlapping associations** | OS/PFS of probiotics + ICIs, NSCLC | |
| **Individual study** |  | |
| Dizman 2022 | 0 | 0 |
| Miura 2021 | 0 | 1 |
| Spencer 2021 | 0 | 0 |
| Svaton 2020 | 1 | 1 |
| Takada 2021 | 1 | 1 |
| Takada 2022 | 0 | 1 |
| Tomita 2020 | 1 | 1 |
| **Decision to retain** | ✘ | **✓** |
| **Grand Total (N)** | 8 |  |
| **Rows (r)** | 7 |  |
| **Columns (c)** | 2 |  |
| **CCA** | 14.3% |  |

**17. OS/PFS of NSAIDs + ICIs**

| **Meta-analysis** | Mao 2022 ^33^ | Zhang 2021 ^34^ |
| --- | --- | --- |
| **Overlapping associations** | OS/PFS of NSAIDs + ICIs | |
| **Individual study** |  | |
| Cortellini 2020 | 1 | 1 |
| Failing 2016 | 1 | 1 |
| Kanai 2021 | 1 | 1 |
| Miura 2021 | 1 | 0 |
| Svaton 2020 | 1 | 1 |
| Wang 2020 | 1 | 1 |
| Decision to retain | **✓** | ✘ |
| **Grand Total (N)** | 11 |  |
| **Rows (r)** | 6 |  |
| **Columns (c)** | 2 |  |
| **CCA** | 83.3% |  |

**18. OS/PFS of + β-blockers ICIs**

| **Meta-analysis** | Zhang 2021 ^34^ | Yan 2022 ^35^ | Kennedy 2022 ^36^ |
| --- | --- | --- | --- |
| **Overlapping associations** | OS/PFS of + β-blockers ICIs | | |
| **Individual study** |  | | |
| Alves 2021 | 0 | 1 | 1 |
| Cortellini 2020 | 1 | 1 | 1 |
| Failing 2016 | 1 | 1 | 1 |
| Hong 2020 | 0 | 0 | 1 |
| Kichenadasse 2021 | 0 | 1 | 1 |
| Kokolus 2018 | 0 | 1 | 0 |
| Mellgard 2022 | 0 | 1 | 0 |
| Michael 2020 | 1 | 0 | 0 |
| Oh 2021 | 0 | 1 | 1 |
| Oren 2020 | 0 | 1 | 1 |
| Wang 2020 | 1 | 1 | 1 |
| Wu 2022 | 0 | 1 | 0 |
| **Decision to retain** | ✘ | **✓** | ✘ |
| **Grand Total (N)** | 22 |  | |
| **Rows (r)** | 12 |  |  |
| **Columns (c)** | 3 |  |  |
| **CCA** | 41.7% |  |  |

**19.** **OS/PFS of opioids + ICIs**

| **Meta-analysis** | Mao 2022 ^33^ | Ju 2022 ^37^ |
| --- | --- | --- |
| **Overlapping associations** | OS/PFS of opioids + ICIs | |
| **Individual study** |  | |
| Botticelli 2021 | 1 | 1 |
| Cortellini 2020 | 1 | 1 |
| Gaucher 2021 | 1 | 1 |
| Iglesias 2020 | 1 | 1 |
| Kostine 2021 | 0 | 1 |
| Miura 2021 | 1 | 1 |
| Taniguchi 2020 | 1 | 1 |
| **Decision to retain** | ✘ | **✓** |
| **Grand Total (N)** | 13 |  |
| **Rows (r)** | 7 |  |
| **Columns (c)** | 2 |  |
| **CCA** | 85.7% |  |

**Abbreviations:** ATB, Antibiotics; CCA, Corrected covered area; ICIs, Immune-checkpoint inhibitors; NSAIDs, Nonsteroidal anti-inflammatory drugs; NSCLC, Non-small cell lung cancer; OS, Overall survival; ORR, Overall response rate; PFS, Progression-free survival; PPIs, Proton pump inhibitors; RCC, Renal cell carcinoma; UC, Urothelial carcinoma.

Formula for calculating the CCA (%) = N-r / rc-r.

Where N = number of included publications (sum of checked boxes), r = number of rows (Individual publications), c = number of columns (number of meta-analyses).

**Reference**

1. Li C, Xia Z, Li A, Meng J. The effect of proton pump inhibitor uses on outcomes for cancer patients treated with immune checkpoint inhibitors: a meta-analysis. *Ann Transl Med*. Dec 2020;8(24):1655. doi:10.21037/atm-20-7498

2. Li M, Zeng C, Yao J, Ge Y, An G. The association between proton pump inhibitors use and clinical outcome of patients receiving immune checkpoint inhibitors therapy. *Int Immunopharmacol*. Nov 2020;88:106972. doi:10.1016/j.intimp.2020.106972

3. Liu C, Guo H, Mao H, Tong J, Yang M, Yan X. An Up-To-Date Investigation Into the Correlation Between Proton Pump Inhibitor Use and the Clinical Efficacy of Immune Checkpoint Inhibitors in Advanced Solid Cancers: A Systematic Review and Meta-Analysis. *Front Oncol*. 2022;12:753234. doi:10.3389/fonc.2022.753234

4. Qin BD, Jiao XD, Zhou XC, et al. Effects of concomitant proton pump inhibitor use on immune checkpoint inhibitor efficacy among patients with advanced cancer. *Oncoimmunology*. 2021;10(1):1929727. doi:10.1080/2162402X.2021.1929727

5. Chen B, Yang C, Dragomir MP, et al. Association of proton pump inhibitor use with survival outcomes in cancer patients treated with immune checkpoint inhibitors: a systematic review and meta-analysis. *Ther Adv Med Oncol*. 2022;14:17588359221111703. doi:10.1177/17588359221111703

6. Wu B, Sun C, Sun X, Li X. Effect of proton pump inhibitors on the clinical outcomes of PD-1/PD-L1 inhibitor in solid cancer patients. *Medicine (Baltimore)*. Sep 9 2022;101(36):e30532. doi:10.1097/MD.0000000000030532

7. Deng R, Zhang H, Li Y, Shi Y. Effect of Antacid Use on Immune Checkpoint Inhibitors in Advanced Solid Cancer Patients: A Systematic Review and Meta-analysis. *J Immunother*. Feb-Mar 01 2023;46(2):43-55. doi:10.1097/CJI.0000000000000442

8. Wei N, Zheng B, Que W, Zhang J, Liu M. The association between proton pump inhibitor use and systemic anti-tumour therapy on survival outcomes in patients with advanced non-small cell lung cancer: A systematic review and meta-analysis. *Br J Clin Pharmacol*. Jul 2022;88(7):3052-3063. doi:10.1111/bcp.15276

9. Hu DH, Wong WC, Zhou JX, et al. The Correlation between the Use of the Proton Pump Inhibitor and the Clinical Efficacy of Immune Checkpoint Inhibitors in Non-Small Cell Lung Cancer. *J Oncol*. 2022;2022:1001796. doi:10.1155/2022/1001796

10. Dar S, Merza N, Qatani A, et al. Impact of proton-pump inhibitors on the efficacy of immune checkpoint inhibitors in non-small cell lung cancer: A systematic review and meta-analysis. *Ann Med Surg (Lond)*. Jun 2022;78:103752. doi:10.1016/j.amsu.2022.103752

11. Rizzo A, Santoni M, Mollica V, et al. The Impact of Concomitant Proton Pump Inhibitors on Immunotherapy Efficacy among Patients with Urothelial Carcinoma: A Meta-Analysis. *J Pers Med*. May 20 2022;12(5)doi:10.3390/jpm12050842

12. Zhang L, Chen C, Chai D, et al. Effects of PPIs use on clinical outcomes of urothelial cancer patients receiving immune checkpoint inhibitor therapy. *Front Pharmacol*. 2022;13:1018411. doi:10.3389/fphar.2022.1018411

13. Wu Q, Liu J, Wu S, Xie X. The impact of antibiotics on efficacy of immune checkpoint inhibitors in malignancies: A study based on 44 cohorts. *Int Immunopharmacol*. Mar 2021;92:107303. doi:10.1016/j.intimp.2020.107303

14. Xu H, Xu X, Wang H, Ge W, Cao D. The association between antibiotics use and outcome of cancer patients treated with immune checkpoint inhibitors: A systematic review and meta-analysis. *Crit Rev Oncol Hematol*. May 2020;149:102909. doi:10.1016/j.critrevonc.2020.102909

15. Yu Y, Zheng P, Gao L, et al. Effects of Antibiotic Use on Outcomes in Cancer Patients Treated Using Immune Checkpoint Inhibitors: A Systematic Review and Meta-Analysis. *J Immunother*. Feb-Mar 01 2021;44(2):76-85. doi:10.1097/CJI.0000000000000346

16. Huang XZ, Gao P, Song YX, et al. Antibiotic use and the efficacy of immune checkpoint inhibitors in cancer patients: a pooled analysis of 2740 cancer patients. *Oncoimmunology*. 2019;8(12):e1665973. doi:10.1080/2162402X.2019.1665973

17. Huang L, Chen X, Zhou L, et al. Antibiotic exposure windows and the efficacy of immune checkpoint blockers in patients with cancer: a meta-analysis. *Ann Palliat Med*. Mar 2021;10(3):2709-2722. doi:10.21037/apm-20-2076

18. Wilson BE, Routy B, Nagrial A, Chin VT. The effect of antibiotics on clinical outcomes in immune-checkpoint blockade: a systematic review and meta-analysis of observational studies. *Cancer Immunol Immunother*. Mar 2020;69(3):343-354. doi:10.1007/s00262-019-02453-2

19. Zhou J, Huang G, Wong WC, et al. The impact of antibiotic use on clinical features and survival outcomes of cancer patients treated with immune checkpoint inhibitors. *Front Immunol*. 2022;13:968729. doi:10.3389/fimmu.2022.968729

20. Petrelli F, Iaculli A, Signorelli D, et al. Survival of Patients Treated with Antibiotics and Immunotherapy for Cancer: A Systematic Review and Meta-Analysis. *J Clin Med*. May 13 2020;9(5)doi:10.3390/jcm9051458

21. Tsikala-Vafea M, Belani N, Vieira K, Khan H, Farmakiotis D. Use of antibiotics is associated with worse clinical outcomes in patients with cancer treated with immune checkpoint inhibitors: A systematic review and meta-analysis. *Int J Infect Dis*. May 2021;106:142-154. doi:10.1016/j.ijid.2021.03.063

22. Yang M, Wang Y, Yuan M, et al. Antibiotic administration shortly before or after immunotherapy initiation is correlated with poor prognosis in solid cancer patients: An up-to-date systematic review and meta-analysis. *Int Immunopharmacol*. Nov 2020;88:106876. doi:10.1016/j.intimp.2020.106876

23. Jiang S, Geng S, Chen Q, et al. Effects of Concomitant Antibiotics Use on Immune Checkpoint Inhibitor Efficacy in Cancer Patients. *Front Oncol*. 2022;12:823705. doi:10.3389/fonc.2022.823705

24. Crespin A, Bandinelli P-A, Bescop CL, et al. 278 Systematic review and meta-analysis evaluating the impact of antibiotic use on clinical outcomes of non-small-cell lung cancer patients treated with immune checkpoint inhibitors. *Journal for ImmunoTherapy of Cancer*. 2021;9(Suppl 2):A301-A302. doi:10.1136/jitc-2021-SITC2021.278

25. Lurienne L, Cervesi J, Duhalde L, et al. NSCLC Immunotherapy Efficacy and Antibiotic Use: A Systematic Review and Meta-Analysis. *J Thorac Oncol*. Jul 2020;15(7):1147-1159. doi:10.1016/j.jtho.2020.03.002

26. Chen H, Han KD, He ZJ, Huang YS. How to Choose a Survival Period? The Impact of Antibiotic Use on OS or PFS in NSCLC Patients Treated With Immune Checkpoint Inhibitors: A Systematic Review and Meta-Analysis. *Technol Cancer Res Treat*. Jan-Dec 2021;20:15330338211033498. doi:10.1177/15330338211033498

27. Bandinelli P-A, Cervesi J, Bescop CL, Buffet R, Gunzburg JD, Zalcman G. 671 Update of a systematic review and meta-analysis studying the association between antibiotic use and clinical outcomes of non-small-cell lung cancer patients treated with immune checkpoint inhibitors. *Journal for ImmunoTherapy of Cancer*. 2020;8(Suppl 3):A402-A403. doi:10.1136/jitc-2020-SITC2020.0671

28. Luo Z, Hao S, Li Y, et al. The negative effect of antibiotics on RCC patients with immunotherapy: A systematic review and meta-analysis. *Front Immunol*. 2022;13:1065004. doi:10.3389/fimmu.2022.1065004

29. Petrelli F, Signorelli D, Ghidini M, et al. Association of Steroids use with Survival in Patients Treated with Immune Checkpoint Inhibitors: A Systematic Review and Meta-Analysis. *Cancers (Basel)*. Feb 27 2020;12(3)doi:10.3390/cancers12030546

30. Wang Y, Yang M, Tao M, et al. Corticosteroid administration for cancer-related indications is an unfavorable prognostic factor in solid cancer patients receiving immune checkpoint inhibitor treatment. *Int Immunopharmacol*. Oct 2021;99:108031. doi:10.1016/j.intimp.2021.108031

31. Zhang L, Jin Q, Chai D, et al. The correlation between probiotic use and outcomes of cancer patients treated with immune checkpoint inhibitors. *Front Pharmacol*. 2022;13:937874. doi:10.3389/fphar.2022.937874

32. Wan L, Wu C, Wu Q, Luo S, Liu J, Xie X. Impact of probiotics use on clinical outcomes of immune checkpoint inhibitors therapy in cancer patients. *Cancer Med*. Jan 2023;12(2):1841-1849. doi:10.1002/cam4.4994

33. Mao Z, Jia X, Jiang P, et al. Effect of Concomitant Use of Analgesics on Prognosis in Patients Treated With Immune Checkpoint Inhibitors: A Systematic Review and Meta-Analysis. *Front Immunol*. 2022;13:861723. doi:10.3389/fimmu.2022.861723

34. Zhang Y, Chen H, Chen S, Li Z, Chen J, Li W. The effect of concomitant use of statins, NSAIDs, low-dose aspirin, metformin and beta-blockers on outcomes in patients receiving immune checkpoint inhibitors: a systematic review and meta-analysis. *Oncoimmunology*. 2021;10(1):1957605. doi:10.1080/2162402X.2021.1957605

35. Yan X, Liu P, Li D, et al. Novel evidence for the prognostic impact of beta-blockers in solid cancer patients receiving immune checkpoint inhibitors. *Int Immunopharmacol*. Dec 2022;113(Pt A):109383. doi:10.1016/j.intimp.2022.109383

36. Kennedy OJ, Neary MT. Brief Communication on the Impact of beta-blockers on Outcomes in Patients Receiving Cancer Immunotherapy. *J Immunother*. Sep 1 2022;45(7):303-306. doi:10.1097/CJI.0000000000000433

37. Ju M, Gao Z, Liu X, et al. The negative impact of opioids on cancer patients treated with immune checkpoint inhibitors: a systematic review and meta-analysis. *J Cancer Res Clin Oncol*. Dec 20 2022;doi:10.1007/s00432-022-04513-0
