# Supplementary material for: Impact of concomitant medications on the efficacy of immune checkpoint inhibitors: an umbrella review
Source: Front Immunol. 2023 Sep 29;14:1218386. doi: 10.3389/fimmu.2023.1218386 (PMC10570520; doi:10.3389/fimmu.2023.1218386)
Supplement: Supplementary file 1 [file DataSheet_1.zip › Supplementary_Materials/Table S3.docx]

**Table S3.** List of excluded studies and reasons for their exclusion.

| First Author | Year | Title | Reason for Exclusion |
| --- | --- | --- | --- |
| Akl EA | 2017 | Parenteral anticoagulation in ambulatory patients with cancer | not related to the topic |
| Hangrui Wang | 2022 | Effect of proton pump inhibitors on the efficacy of immune checkpoint inhibitors in cancer patients: a meta-analysis | non-English language (Chinese) |
| A. Garant | 2017 | Concomitant use of corticosteroids and immune checkpoint inhibitors in patients with hematologic or solid neoplasms: A systematic review | no meta-analysis |
| Armani B Hayes | 2020 | Impact of antibiotic exposure on theoverall survival in patients receiving immune checkpoint inhibitors: Does race matter? | no full text |
| Hajir Ibraheim | 2020 | Systematic review with meta-analysis: effectiveness of anti-inflammatory therapy in immune checkpoint inhibitor-induced enterocolitis | not related to the topic |
| Kahale LA | 2017 | Oral anticoagulation in people with cancer who have no therapeutic or prophylactic indication for anticoagulation | not related to the topic |
| AR Li | 2021 | Prognostic role of beta-blockers for Melanoma: A systematic review and meta-analysis | no full text |
| Jiarui Li | 2020 | Impact of corticosteroids use on efficacy of immune checkpoint inhibitors in cancer patients: A meta-analysis. | no full text |
| Shuyan Li | 2020 | A narrative review of synergistic drug administration in unresectable locally advanced non-small cell lung cancer: current landscape and future prospects in the era of immunotherapy | no meta-analysis |
| Xiaofeng Luo | 2021 | Metformin Adjunct With Antineoplastic Agents for the Treatment of Lung Cancer: A Meta-Analysis of Randomized Controlled Trials and Observational Cohort Studies | no analysis of ICIs |
| Natansh D. Modi | 2021 | Commentary: Association Between Antihypertensive Medication Use and Breast Cancer: A Systematic Review and Meta-Analysis | comment |
| J. Pierrard | 2019 | Impact of the gut microbiome on immune checkpoint inhibitor efficacy-a systematic review | no meta-analysis |
| Wu Ye | 2021 | Comparison of monoclonal antibodies targeting CD38, SLAMF7 and PD-1/PD-L1 in combination with Bortezomib/Immunomodulators plus dexamethasone/prednisone for the treatment of multiple myeloma: an indirect-comparison Meta-analysis of randomised controlled trials | not related to the topic |
| Gengwei Huo | 2021 | Effect of antibiotics on treatment efficacy of immune checkpoint inhibitors in non-small cell lung cancer: a meta-analysis | non-English language (Chinese) |
| Smathorn Thakolwiboon | 2021 | Meta-analysis of effectiveness of steroid-sparing attack prevention in MOG-IgG-associated disorder | not related to the topic |
| J. Cervesi | 2020 | A review of meta-analyses on the impact of antibiotics on the efficacy of immune checkpoint inhibitors and cancer patients’ survival | no meta-analysis |
| Bing Dai | 2020 | Impact of hormonal preconditioning on efficacy and safety of immune checkpoint inhibitors in patients with non-small-cell lung cancer: a network meta-analysis | network meta-analysis |
| Ganessan Kichenadasse | 2021 | Effect of concomitant use of antihypertensives and immune check point inhibitors on cancer outcomes | no meta-analysis |
| Sebastiano Buti | 2021 | Effect of concomitant medications with immunemodulatory properties on the outcomes of patients with advanced cancer treated with immune checkpoint inhibitors: development and validation of a novel prognostic index | individual studies |
| Christopher Ma | 2022 | Pharmacological Interventions for the Prevention and Treatment of Immune Checkpoint Inhibitor-Associated Enterocolitis: A Systematic Review | no meta-analysis |
| Yu Chang | 2022 | The association between baseline proton pump inhibitors, immune checkpoint inhibitors, and chemotherapy: a systematic review with network meta-analysis | network meta-analysis |
| Jiuhang Yu | 2022 | Effect of concomitant antibiotics use on patient outcomes and adverse effects in patients treated with ICIs | no meta-analysis |
| Rahmad Aji Prasetya | 2021 | Concomitant use of analgesics and immune checkpoint inhibitors in non-small cell lung cancer: A pharmacodynamics perspective | no meta-analysis |
| Jiao He | 2021 | Effects of antibacterials on the immunotherapy of NSCLC: a meta-analysis | non-English language (Chinese) |
| Sara Elena Rebuzzi | 2021 | Prognostic and Predictive Factors in Advanced Urothelial Carcinoma Treated with Immune Checkpoint Inhibitors: A Review of the Current Evidence | not related to the topic |
| Giovanni Rossi | 2019 | Concomitant medications during immune checkpoint blockage in cancer patients: Novel insights in this emerging clinical scenario | no meta-analysis |
| Yeon-Hee Baek | 2022 | Survival outcomes of patients with nonsmall cell lung cancer concomitantly receiving proton pump inhibitors and immune checkpoint inhibitors | individual studies |
| Milo Gatti | 2021 | Influenza Vaccination and Myo-Pericarditis in Patients Receiving Immune Checkpoint Inhibitors: Investigating the Likelihood of Interaction through the Vaccine Adverse Event Reporting System and VigiBase | no meta-analysis |
| Sha Zhao | 2019 | Antibiotics are associated with attenuated efficacy of anti-PD-1/PD-L1 therapies in Chinese patients with advanced non-small cell lung cancer | individual studies |
| Shintaro Minegish | 2022 | Immune Checkpoint Inhibitors Do Not Increase Short-Term Risk of Hypertension in Cancer Patients: a Systematic Literature Review and Meta-Analysis | not related to the topic |
| J Pierrard | 2019 | Impact of the gut microbiome on immune checkpoint inhibitor efficacy-a systematic review | no meta-analysis |
| Brendan Sieber | 2022 | Concomitant Medication Effects on Immune Checkpoint Inhibitor Efficacy and Toxicity | no meta-analysis |
| Jianyi Yin | 2022 | Chronic Use of Proton Pump Inhibitors Is Associated With an Increased Risk of Immune Checkpoint Inhibitor Colitis in Renal Cell Carcinoma | individual studies |
| Marie Kostine | 2021 | Baseline co-medications may alter the anti-tumoural effect of checkpoint inhibitors as well as the risk of immune-related adverse events | no meta-analysis |
| Eiko Hayase | 2021 | Role of the intestinal microbiome and microbial-derived metabolites in immune checkpoint blockade immunotherapy of cancer | no meta-analysis |
| Mitchell S von Itzstein | 2022 | Association between Antibiotic Exposure and Systemic Immune Parameters in Cancer Patients Receiving Checkpoint Inhibitor Therapy | individual studies |
| Amit A Kulkarni | 2020 | Comparative analysis of antibiotic exposure association with clinical outcomes of chemotherapy versus immunotherapy across three tumour types | individual studies |
| Hui Qiu | 2022 | Different classes of antibiotics exhibit disparate negative impacts on the therapeutic efficacy of immune checkpoint inhibitors in advanced non-small cell lung cancer patients | individual studies |
| Daniel Spakowicz | 2020 | Inferring the role of the microbiome on survival in patients treated with immune checkpoint inhibitors: causal modeling, timing, and classes of concomitant medications | individual studies |
| Tsvetelina Velikova | 2021 | Antibiotic-Related Changes in Microbiome: The Hidden Villain behind Colorectal Carcinoma Immunotherapy Failure | comment |
| Sajad Najafi | 2022 | The impact of microbiota on PD-1/PD-L1 inhibitor therapy outcomes: A focus on solid tumors | comment |
| Eric J Lehrer | 2019 | Treatment of brain metastases with stereotactic radiosurgery and immune checkpoint inhibitors: An international meta-analysis of individual patient data | not related to the topic |
