# Supplementary material for: Impact of concomitant medications on the efficacy of immune checkpoint inhibitors: an umbrella review
Source: Front Immunol. 2023 Sep 29;14:1218386. doi: 10.3389/fimmu.2023.1218386 (PMC10570520; doi:10.3389/fimmu.2023.1218386)
Supplement: Supplementary file 1 [file DataSheet_1.zip › Supplementary_Materials/Table S1.docx]

**Table S1**. Search strategy and history in PubMed

| Search Number | Subject heading | Free-text terms | Results |
| --- | --- | --- | --- |
| #1 | Immune Checkpoint Inhibitors | PD L1 inhibitor* | 161,666 |
|  |  | programmed death ligand 1 inhibitor* |  |
|  |  | PD 1 PD L1 blockade |  |
|  |  | blockade PD 1 PD L1 |  |
|  |  | CTLA-4 |  |
|  |  | anti-CTLA-4 |  |
|  |  | cytotoxic t lymphocyte associated protein 4 inhibitor* |  |
|  |  | PD-1 |  |
|  |  | PD-L1 |  |
|  |  | PD 1 L1 |  |
|  |  | PD-1-PD-L1 |  |
|  |  | ICI |  |
|  |  | ICIs |  |
|  |  | Immunotherapy |  |
|  |  | avelumab |  |
|  |  | durvalumab |  |
|  |  | tremelimumab |  |
|  |  | pembrolizumab |  |
|  |  | camrelizumab |  |
|  |  | ipilimumab |  |
|  |  | tislelizumab |  |
|  |  | SHR-1210 |  |
|  |  | toripalimab |  |
|  |  | penpulimab |  |
|  |  | nivolumab |  |
|  |  | atezolizumab |  |
|  |  | sintilimab |  |
|  |  | Opdivo |  |
|  |  | Keytruda |  |
| #2 | meta-analysis | meta analys* | 403,348 |
|  |  | systematic review* |  |
|  |  | meta-analys* |  |
|  |  | metaanalys* |  |
|  |  | systematic literature review |  |
| #3 | concomitant medications | co-medication | 1,667,606 |
|  |  | baseline |  |
|  |  | baselines |  |
|  |  | co-medications |  |
|  |  | concomitan* |  |
|  |  | gastric acid suppressants |  |
|  |  | proton pump inhibitors |  |
|  |  | PPI |  |
|  |  | H2 antagonists |  |
|  |  | Pantoprazole |  |
|  |  | lansoprazole |  |
|  |  | omeprazole |  |
|  |  | rabeprazole |  |
|  |  | esomeprazole |  |
|  |  | non steroidal anti inflammatory drugs |  |
|  |  | NSAID* |  |
|  |  | cyclooxygenase inhibitors |  |
|  |  | cox 2 inhibitors |  |
|  |  | aspirin |  |
|  |  | corticosteroids |  |
|  |  | steroids |  |
|  |  | Glucocorticoids |  |
|  |  | prednisone |  |
|  |  | immunomodulators |  |
|  |  | psychotropic drugs |  |
|  |  | clozapine |  |
|  |  | chordiazepoxide |  |
|  |  | olanzapine |  |
|  |  | Zyprexa |  |
|  |  | olanza |  |
|  |  | risperidone |  |
|  |  | analgesics |  |
|  |  | opioids |  |
|  |  | Morphine |  |
|  |  | antidiabetics |  |
|  |  | metformin |  |
|  |  | antidiabetic drugs |  |
|  |  | insulin |  |
|  |  | Antibiotics |  |
|  |  | ATB |  |
|  |  | antibacterial |  |
|  |  | broad spectrum antibiotic |  |
|  |  | beta-lactam |  |
|  |  | sulphonamide |  |
|  |  | quinolone |  |
|  |  | fluoroquinolones |  |
|  |  | macrolide |  |
|  |  | aminoglycoside |  |
|  |  | tetracycline |  |
|  |  | captopril |  |
|  |  | beta-blockers |  |
|  |  | angiotensin ii receptor blockers |  |
|  |  | ARB |  |
|  |  | candesartan |  |
|  |  | angiotensin converting enzyme inhibitors |  |
|  |  | ACEI |  |
|  |  | calcium antagonists |  |
|  |  | anticoagulants |  |
|  |  | low molecular weight heparin |  |
|  |  | cumarinic |  |
|  |  | acetylsalicylic acid |  |
|  |  | lipid lowering agents |  |
|  |  | fibrates |  |
|  |  | ezetimibe |  |
|  |  | statin |  |
|  |  | probiotics |  |
| #1 AND #2 AND #3 |  |  | 273 |

**·**Asterisk wildcard (*) - Is used between words where variations may be possible. Example: "meta-analys*" can return results such as, " meta-analysis" or "meta-analyses"

·Search scope is title/abstract
