# Supplementary material for: Impact of concomitant medications on the efficacy of immune checkpoint inhibitors: an umbrella review
Source: Front Immunol. 2023 Sep 29;14:1218386. doi: 10.3389/fimmu.2023.1218386 (PMC10570520; doi:10.3389/fimmu.2023.1218386)
Supplement: Supplementary file 1 [file DataSheet_1.zip › Supplementary_Materials/Table S7.docx]

**Table S7.** Recalculated detailed results of meta-analyses using the by trim-and-fill method.

| **Author** | **CM** | **Cancer type** | **No. of studies** | **Outcome** | **Metric** | **RA p-value** | **RA ES (95% CI)** | **Egger p-Value** | **Level of evidence** | **TFMA ES (95% CI)** | **TFMA p-value** | **No. of added studies ^§^** |
| --- | --- | --- | --- | --- | --- | --- | --- | --- | --- | --- | --- | --- |
| Chen, Baoqing 2022 | PPIs | NSCLC | 16 | OS | HR | 0.0000885 | 1.331[1.154, 1.535] | 0.0371 | Ⅲ | 1.1360 [0.9792, 1.3180] | 0.0924 ^†^ | 6 |
| Zhang 2022 | PPIs | UC | 6 | PFS | HR | 5.06E-09 | 1.535[1.33, 1.773] | 0.0331 | Ⅳ | 1.4369 [1.2773, 1.6165] | < 0.0001 | 3 |
| Wu 2021 | ATB | Multiple | 48 | OS | HR | 1.17E-15 | 1.198[1.146, 1.253] | 4.01E-07 | Ⅲ | 1.0467 [0.9641, 1.1364] | 0.2766 ^†^ | 19 |
| Wu 2021 | ATB | Multiple | 40 | PFS | HR | 1.81E-08 | 1.174[1.11, 1.242] | 0.00808 | Ⅱ | 1.0946 [1.0175, 1.1775] | 0.0153 | 11 |
| Wu 2021 | ATB | NSCLC | 27 | OS | HR | 0.00000113 | 1.255[1.146, 1.376] | 0.0264 | Ⅲ | 1.1331 [1.0099, 1.2712] | 0.0333 | 7 |
| Crespin 2021 | ATB [-60,0] | NSCLC | 13 | OS | HR | 1.99E-09 | 2.277[1.74, 2.98] | 0.000583 | Ⅱ | 1.4205 [0.9422, 2.1418] | 0.0938 ^†^ | 7 |
| Crespin 2021 | ATB [-60,0] | NSCLC | 9 | PFS | HR | 0.0000132 | 1.677[1.329, 2.116] | 0.00855 | Ⅳ | 1.4481 [1.0695, 1.9609] | 0.0167 | 3 |
| Wu 2021 | ATB | RCC | 5 | OS | HR | 0.028 | 1.122[1.013, 1.244] | 0.0242 | Ⅳ | 1.0869 [0.9875, 1.1964] | 0.0886 ^†^ | 2 |
| Wu 2021 | ATB | RCC | 7 | PFS | HR | 1.19E-09 | 1.299[1.194, 1.413] | 0.00228 | Ⅳ | 1.2358 [1.1364, 1.3439] | < 0.0001 | 4 |
| Zhang, Lilong 2022 | ATB | HCC | 4 | PFS | HR | 0.451 | 1.224[0.582, 2.573] | 0.0218 | ns | 0.8525 [0.4791, 1.5169] | 0.5874 | 2 |
| Wang 2021 | Steroids (cancer indication) | Multiple | 8 | PFS | HR | 0.00000206 | 1.737[1.383, 2.183] | 0.0297 | Ⅲ | 1.5268 [1.0531, 2.2134] | 0.0255 | 3 |
| Zhang, Hongchuan 2021 | Steroids | NSCLC | 11 | OS | HR | 1.01E-10 | 1.816[1.516, 2.177] | 0.0498 | Ⅱ | 1.6337 [1.3267, 2.0118] | < 0.0001 | 3 |
| Zhang, Hongchuan 2021 | Steroids | NSCLC | 11 | PFS | HR | 3.37E-08 | 1.695[1.405, 2.044] | 0.0152 | Ⅳ | 1.4382 [1.1380, 1.8176] | 0.0024 | 4 |
| Yan 2022 | Beta blockers | Multiple | 7 | PFS | HR | 0.568 | 0.977[0.903, 1.058] | 0.0369 | ns | 1.0038 [0.9328, 1.0803] | 0.9185 | 3 |
| Ju 2022 | Opioids | Multiple | 5 | PFS | HR | 6.34E-13 | 1.611[1.415, 1.834] | 0.014 | Ⅳ | 1.5506 [1.3765, 1.7466] | < 0.0001 | 2 |
| Mao 2022 | NSAIDs | Multiple | 8 | OS | HR | 0.423 | 0.91[0.723, 1.146] | 0.0164 | ns | 1.0600 [0.7774, 1.4454] | 0.7127 | 3 |
| Mao 2022 | NSAIDs | Multiple | 8 | ORR | OR | 0.193 | 1.398[0.845, 2.316] | 0.0366 | ns | 1.0023 [0.4914, 2.0446] | 0.9949 | 2 |

^†^ The statistical significance of the pooled effect size changed after trim-and-fill analysis.

**^§^** The number of studies added by the trim-and-fill analysis.

**Abbreviation:** ATB, antibiotics; CM, concomitant medications; CI, confidence interval; ES, effect size; HR, hazard ratio; NSAIDs, nonsteroidal anti-inflammatory agents; NSCLC, non-small cell lung cancer; OS, overall survival; OR, odds ratio; ORR, objective response rate; PPIs, proton pump inhibitors; PFS, progression-free survival; RA, re-analysis; RCC, renal cell carcinoma; TFMA, trim-and-fill method analysis; UC, urothelial carcinoma; Ⅱ, highly suggestive evidence (class Ⅱ); Ⅲ, suggestive evidence (class Ⅲ); Ⅳ, weak evidence (class Ⅳ); ns, non-significant (class ns).
