# Supplementary material for: Impact of concomitant medications on the efficacy of immune checkpoint inhibitors: an umbrella review
Source: Front Immunol. 2023 Sep 29;14:1218386. doi: 10.3389/fimmu.2023.1218386 (PMC10570520; doi:10.3389/fimmu.2023.1218386)
Supplement: Supplementary file 1 [file DataSheet_1.zip › Supplementary_Materials/Table S6.docx]

**Table S6.** Detailed results and evidence of excluded meta-analyses due to overlap regarding the impact of concomitant medications on the efficacy of ICIs

| **Author** | **CM** | **Cancer type** | **No. of studies** | **No. of patients (CM+/CM-)** | **Outcome** | **Metric** | **Reported MA model** | **Reported p-Value** | **Reported ES (95% CI)** | **Reported I^2^(%) (p-Value)** | **ES (95% CI) of largest study** | **consistence of individual study (Y/N)** | **RA Model** | **RA p-value** | **RA effect (95% CI)** | **RA I^2^** | **95% PI** | **Egger p-Value** | **TES p-value** | **Level of evidence** | **Concordant direction** ^†^ |
| --- | --- | --- | --- | --- | --- | --- | --- | --- | --- | --- | --- | --- | --- | --- | --- | --- | --- | --- | --- | --- | --- |
| Li, Chao 2020 | PPIs (any exposure window) | Multiple | 5 | 398/769 | OS | HR | Random | NA | 0.966 (0.486–1.447) | 74.3 (0.004) | 1.45 (1.2-1.75) | Y | DL | 0.633 | 0.895[0.566, 1.413] | 65.692 | [0.202, 3.952] | 0.00775 | 0.599 | ns | N |
| Li, Manyu 2020 | PPIs (any exposure window) | Multiple | 7 | 507/975 | OS | HR | Random | 0.73 | 1.05 (0.79-1.40) | 41.0 (0.12) | 1.29 (1.13-1.47) | N | DL | 0.725 | 1.061[0.764, 1.472] | 50.89 | [0.444, 2.536] | 0.0777 | 0.679 | ns | N |
| Liu 2022 | PPIs (any exposure window) | Multiple | 17 | 4597/5381 | OS | HR | Random | NA | 1.29 (1.10-1.50) | 74.1 (<0.001) | 0.96 (0.89-1.02) | N | DL | 0.00116 | 1.283[1.104, 1.491] | 73.921 | [0.759, 2.168] | 0.114 | 0.0042 | Ⅳ | Y |
| Qin 2021 | PPIs (any exposure window) | Multiple | 7 | 1465/2181 | OS | HR | NA | NA | 1.39 (1.26-1.54) | 36.5 (0.16) | 1.52 (1.27-1.83) | N | DL | 0.0000164 | 1.371[1.188, 1.582] | 36.354 | [0.978, 1.921] | 0.323 | 0.341 | Ⅲ | Y |
| Wu 2022 | PPIs (any exposure window) | Multiple | 8 | 1153/1902* | OS | HR | Fixed | NA | 1.43(1.32-1.56) | 11.8(0.338) | 1.26(1.04-1.52) | N | DL | 6.88E-15 | 1.432[1.308, 1.567] | 11.542 | [1.224, 1.675] | 0.357 | 0.319 | Ⅰ | Y |
| Deng 2022 | PPIs (any exposure window) | Multiple | 29 | 7144/9791 | OS | HR | Random | <0.00001 | 1.40(1.25-1.57) | 78(<0.0001) | 0.96(0.89-1.04) | N | DL | 1.04E-08 | 1.399[1.247, 1.57] | 77.805 | [0.853, 2.296] | 0.121 | 0.00000144 | Ⅲ | Y |
| Li, Chao 2020 | PPIs (any exposure window) | Multiple | 5 | 398/769 | PFS | HR | Random | NA | 0.858 (0.388–1.328) | 87.7 (<0.001) | 1.30 (1.1-1.53) | Y | DL | 0.247 | 0.757[0.473, 1.212] | 79.079 | [0.146, 3.93] | 0.0106 | 0.121 | ns | N |
| Li, Manyu 2020 | PPIs (any exposure window) | Multiple | 6 | 460/932 | PFS | HR | Random | 0.51 | 0.90 (0.66-1.23) | 64 (0.02) | 1.17 (1.04-1.32) | N | DL | 0.57 | 0.905[0.643, 1.275] | 69.388 | [0.318, 2.581] | 0.0244 | 0.233 | ns | N |
| Liu 2022 | PPIs (any exposure window) | Multiple | 11 | 1624/2707 | PFS | HR | Random | NA | 1.19 (0.98-1.44) | 75.2 (<0.001) | 1.38 (1.18-1.62) | N | DL | 0.112 | 1.172[0.963, 1.426] | 76.442 | [0.62, 2.214] | 0.187 | 0.0196 | ns | N |
| Qin 2021 | PPIs (any exposure window) | Multiple | 5 | 1314/2025 | PFS | HR | NA | NA | 1.28 (1.17-1.40) | 31.3 (0.21) | 1.38 (1.18-1.62) | N | DL | 0.000161 | 1.261[1.118, 1.422] | 31.341 | [0.926, 1.715] | 0.0185 | 0.373 | Ⅲ | Y |
| Wu 2022 | PPIs (any exposure window) | Multiple | 8 | 1153/1902* | PFS | HR | Fixed | NA | 1.32(1.23-1.42) | 0(0.628) | 1.26(1.07-1.48) | N | DL | 7.59E-15 | 1.323[1.233, 1.42] | 0 | [1.212, 1.445] | 0.877 | 0.272 | Ⅰ | Y |
| Deng 2022 | PPIs (any exposure window) | Multiple | 23 | 3132/4969* | PFS | HR | Random | <0.00001 | 1.34(1.19-1.62) | 72(<0.0001) | 1.47(1.26-1.71) | N | DL | 0.00000263 | 1.342[1.187, 1.517] | 71.665 | [0.834, 2.16] | 0.832 | 0.0000265 | Ⅲ | Y |
| Li, Chao 2020 | PPIs (any exposure window) | NSCLC | 2 | 275/591 | OS | HR | Random | NA | 1.103 (0.353–1.854) | 83.4 (0.014) | 1.45 (1.2-1.75) | Y | HKSJ | 0.859 | 1.086[0.01, 114.32] | 73.321 | < 3 studies | < 3 studies | NA | ns | N |
| Li, Manyu 2020 | PPIs (any exposure window) | NSCLC | 4 | 385/795 | OS | HR | Random | 0.05 | 1.24 (1.00-1.55) | 17 (0.30) | 1.29 (1.13-1.47) | N | HKSJ | 0.201 | 1.299[0.781, 2.161] | 33.146 | [0.459, 3.676] | 0.527 | 0.627 | ns | N |
| Wei 2022 | PPIs (any exposure window) | NSCLC | 5 | 859/1271 | OS | HR | Random | <0.00001 | 1.42 (1.22-1.65) | 17 (0.31) | 1.49 (1.26-1.77) | N | DL | 0.00000385 | 1.42[1.224, 1.647] | 16.468 | [1.02, 1.977] | 0.365 | 0.657 | Ⅳ | Y |
| Hu 2022 | PPIs (any exposure window) | NSCLC | 11 | 5043/7308 | OS | HR | Random | 0.003 | 1.30(1.10-1.54) | 82(<0.0001) | 1.00(0.85-1.17) | N | DL | 0.00261 | 1.299[1.095, 1.539] | 81.852 | [0.741, 2.276] | 0.0594 | 0.0237 | Ⅳ | Y |
| Dar 2022 | PPIs (any exposure window) | NSCLC | 4 | 762/1423 | OS | HR | Random | <0.0001 | 1.46(1.27-1.67) | 0(0.85) | 1.53(1.21-1.93) | Y | HKSJ | 0.605 | 0.895[0.515, 1.555] | 65.692 | [0.213, 3.755] | 0.00775 | 0.599 | ns | N |
| Li, Chao 2020 | PPIs (any exposure window) | NSCLC | 2 | 275/591 | PFS | HR | Fixed | NA | 1.173 (0.815–1.521) | 49.3 (0.16) | 1.30 (1.1-1.53) | Y | HKSJ | 0.456 | 1.191[0.172, 8.241] | 37.408 | < 3 studies | < 3 studies | NA | ns | N |
| Wei 2022 | PPIs (any exposure window) | NSCLC | 4 | 812/1228 | PFS | HR | Random | <0.00001 | 1.29 (1.16-1.44) | 0 (0.60) | 1.32 (1.13-1.54) | N | HKSJ | 0.00941 | 1.293[1.127, 1.483] | 0 | [1.074, 1.556] | 0.355 | 0.559 | Ⅳ | Y |
| Li, Manyu 2020 | PPIs (any exposure window) | NSCLC | 3 | 338/752 | PFS | HR | Random | 0.006 | 1.17 (1.05-1.31) | 0 (0.50) | 1.17 (1.04-1.32) | N | HKSJ | 0.0767 | 1.269[0.939, 1.716] | 0 | [0.521, 3.094] | 0.577 | 0.573 | ns | N |
| Hu 2022 | PPIs (any exposure window) | NSCLC | 7 | 5387/7912 | PFS | HR | Random | 0.001 | 1.25(1.09-1.42) | 56(0.04) | 0.93(0.76-1.13) | N | DL | 0.00112 | 1.247[1.092, 1.424] | 56.081 | [0.861, 1.805] | 0.73 | 0.26 | Ⅳ | Y |
| Dar 2022 | PPIs (any exposure window) | NSCLC | 4 | 762/1423 | PFS | HR | Random | <0.0001 | 1.31(1.17-1.47) | 0(0.92) | 1.34(1.12-1.60) | Y | HKSJ | 0.859 | 1.086[0.01, 114.32] | 73.321 | < 3 studies | < 3 studies | NA | ns | N |
| Li, Manyu 2020 | PPIs (any exposure window) | Melanoma | 2 | 46/154 | OS | HR | Random | 0.34 | 0.67 (0.30-1.52) | 34 (0.22) | 1.01 (0.40-2.55) | Y | HKSJ | 0.515 | 0.674[0.003, 130.964] | 32.758 | < 3 studies | < 3 studies | NA | ns | Y |
| Li, Manyu 2020 | PPIs (any exposure window) | Melanoma | 2 | 46/154 | PFS | HR | Random | 0.02 | 0.50 (0.28-0.91) | 17 (0.27) | 0.30 (0.10-0.90) | N | HKSJ | 0.264 | 0.501[0.01, 23.962] | 17.115 | < 3 studies | < 3 studies | NA | ns | Y |
| Chen, Baoqing 2022 | PPIs (any exposure window) | UC | 3 | 375/682 | OS | HR | Random | 0.09 | 1.54(1.30-1.82) | 0(NA) | 1.52(1.27-1.82) | N | HKSJ | 0.0205 | 1.536[1.174, 2.011] | 0 | [0.694, 3.4] | 0.875 | 0.182 | Ⅳ | Y |
| Rizzo 2022 | PPIs (any exposure window) | UC | 2 | 340/675 | OS | HR | Fixed | NA | 1.55(1.31-1.84) | 0(NA) | 1.52 (1.27-1.83) | Y | HKSJ | 0.0852 | 1.556[0.731, 3.313] | 0 | < 3 studies | < 3 studies | NA | ns | N |
| Chen, Baoqing 2022 | PPIs (any exposure window) | UC | 4 | 375/682* | PFS | HR | Random | <0.001 | 1.63(1.23-2.15) | 45(NA) | 1.38(1.18-1.62) | N | HKSJ | 0.0341 | 1.63[1.072, 2.48] | 44.549 | [0.609, 4.365] | 0.444 | 0.0144 | Ⅳ | Y |
| Rizzo 2022 | PPIs (any exposure window) | UC | 2 | 340/675 | PFS | HR | Fixed | NA | 1.43(1.23-1.66) | 0(NA) | 1.38(1.18-1.62) | Y | HKSJ | 0.178 | 1.547[0.314, 7.62] | 69.434 | < 3 studies | < 3 studies | NA | ns | N |
| Bandinelli 2020 | ATB [-90,0] | NSCLC | 9 | 323/1021 | OS | HR | Random | NA | 2.29(1.37-3.83) | 74(<0.01) | 1.6(1.11-2.31) | N | DL | 0.000148 | 2.272[1.487, 3.472] | 74.363 | [0.577, 8.943] | 0.0731 | 0.242 | Ⅳ | Y |
| Chen, Hua 2021 | ATB [-60,0] | NSCLC | 6 | 230/737 | OS | HR | Fixed | NA | 2.55(1.98-3.27) | 8(0.365) | 2.00(1.30-3.20) | N | DL | 1.96E-12 | 2.578[1.98, 3.356] | 6.632 | [1.654, 4.018] | 0.272 | 0.323 | Ⅳ | Y |
| Chen, Hua 2021 | ATB [-30,0] | NSCLC | 3 | 89/389 | OS | HR | Fixed | NA | 2.93(2.13-4.04) | 0(0.445) | 2.00(1.30-3.20) | Y | HKSJ | 0.0463 | 2.823[1.043, 7.639] | 39.499 | [0.034, 232.662] | 0.318 | 0.066 | Ⅳ | Y |
| Lurienne 2020 | ATB[-90,0] | NSCLC | 5 | 192/635 | OS | HR | Random | NA | 2.49(0.95-6.51) | 82(<0.01) | 2.00(1.30-3.08) | N | DL | 0.00269 | 2.47[1.369, 4.458] | 81.574 | [0.299, 20.399] | 0.525 | 0.494 | Ⅳ | Y |
| Xu 2020 | ATB (Before ICIs initiation) | NSCLC | 3 | 127/388 | OS | HR | Random | 0.09 | 1.81(0.91-3.63) | 92(<0.0001) | 2.70(1.70-4.29) | Y | HKSJ | 0.18 | 1.813[0.512, 6.423] | 91.753 | [0, 7149.047] | 0.091 | 0.000000774 | ns | N |
| Bandinelli 2020 | ATB [-90,0] | NSCLC | 7 | 283/788 | PFS | HR | Random | NA | 1.69(1.12,2.55) | 60(0.02) | 1.2(0.89-1.61) | N | DL | 0.00139 | 1.66[1.217, 2.266] | 60.021 | [0.672, 4.104] | 0.016 | 0.0262 | Ⅳ | Y |
| Chen, Hua 2021 | ATB [-60,0] | NSCLC | 5 | 179/526 | PFS | HR | Random | NA | 1.53(1.22-1.92) | 64.3(0.024) | 1.20(0.90-1.70) | N | DL | 0.00983 | 1.686[1.134, 2.505] | 59.619 | [0.476, 5.965] | 0.359 | 0.0427 | Ⅳ | Y |
| Chen, Hua 2021 | ATB [-30,0] | NSCLC | 2 | 60/222 | PFS | HR | Random | NA | 1.55(1.14-2.10) | 76(0.041) | 1.30(0.90-1.80) | N | HKSJ | 0.383 | 1.81[0.01, 318.135] | 73.579 | < 3 studies | < 3 studies | NA | ns | N |
| Lurienne 2020 | ATB[-90,0] | NSCLC | 4 | 163/545 | PFS | HR | Random | NA | 1.56(0.78-3.13) | 73(0.141) | 1.20(0.90-1.60) | N | HKSJ | 0.134 | 1.559[0.779, 3.122] | 72.548 | [0.263, 9.229] | 0.518 | 0.398 | ns | N |
| Huang, Litang2021 | ATB [-30,0] | Multiple | 9 | 331/1090 | OS | HR | Random | NA | 2.25(1.42-3.55) | 89.0(0<.001) | 2.50(1.60-3.70) | N | DL | 0.000522 | 2.246[1.422, 3.547] | 89.085 | [0.48, 10.502] | 0.000382 | 1.94E-08 | Ⅳ | Y |
| Huang, Litang2021 | ATB [-60,0] | Multiple | 7 | 313/964 | OS | HR | Random | NA | 1.57(1.16-2.11) | 53.2(0.046) | 1.60(1.10-2.30) | N | DL | 0.00442 | 1.561[1.149, 2.12] | 56.03 | [0.661, 3.685] | 0.461 | 0.0303 | Ⅳ | Y |
| Huang, Litang2021 | ATB [-90,0] | Multiple | 2 | 87/61* | OS | HR | Random | NA | 1.53(0.89-2.62) | 71.9(0.059) | 1.12(0.69-1.82) | N | HKSJ | 0.367 | 1.527[0.046, 50.44] | 71.898 | < 3 studies | < 3 studies | NA | ns | N |
| Huang, Xuanzhang 2019 | ATB (Before ICIs initiation) | Multiple | 11 | 4401335 | OS | HR | Fixed | NA | 2.29(1.92-2.73) | 0.0(0.837) | 2.50(1.60-3.70) | N | DL | 1.74E-20 | 2.285[1.919, 2.721] | 0 | [1.868, 2.795] | 0.999 | 0.712 | Ⅳ | Y |
| Wilson 2019 | ATB[-42,0] | Multiple | 7 | 160/825 | OS | HR | Random | <0.0001 | 3.43(2.29-5.14) | 57.3(NA) | 4.40(2.60-7.60) | N | DL | 1.22E-09 | 3.395[2.289, 5.035] | 58.382 | [1.084, 10.63] | 0.823 | 0.384 | Ⅳ | Y |
| Zhou 2022 | ATB[-30,0] | Multiple | 5 | 265/407 | OS | HR | Random | <0.00001 | 2.61(2.11-3.23) | 0(0.54) | 2.9(1.10-7.65) | Y | DL | 1.19E-18 | 2.608[2.107, 3.228] | 0 | [1.845, 3.687] | 0.214 | 0.0185 | Ⅳ | Y |
| Zhou 2022 | ATB[-60,0] | Multiple | 5 | 453/1868 | OS | HR | Random | <0.00001 | 1.97(1.65-2.35) | 29(0.23) | 1.65(1.29-2.12) | Y | DL | 3.82E-14 | 1.97[1.652, 2.348] | 28.86 | [1.263, 3.071] | 0.316 | 0.215 | Ⅳ | Y |
| Zhou 2022 | ATB[-60,0] | Multiple | 4 | 336/400 | PFS | HR | Random | <0.00001 | 1.88(1.61-2.19) | 0(0.58) | 1.71(1.36-2.16) | N | HKSJ | 0.0022 | 1.879[1.533, 2.301] | 0 | [1.428, 2.472] | 0.967 | 0.684 | Ⅳ | Y |
| Huang, Litang2021 | ATB [-30,0] | Multiple | 8 | 233/842 | PFS | HR | Random | NA | 1.70(1.35-2.14) | 31.8(0.174) | 1.30(0.90-1.80) | N | DL | 0.00000412 | 1.688[1.351, 2.11] | 28.235 | [1.031, 2.766] | 0.0364 | 0.0304 | Ⅳ | Y |
| Huang, Litang2021 | ATB [-60,0] | Multiple | 7 | 313/964 | PFS | HR | Random | NA | 1.45(1.04-2.02) | 71.0(0.002) | 1.20(0.90-1.60) | N | DL | 0.0318 | 1.445[1.033, 2.021] | 72.637 | [0.507, 4.117] | 0.53 | 0.326 | Ⅳ | Y |
| Huang, Litang2021 | ATB [-90,0] | Multiple | 2 | 87/61* | PFS | HR | Random | NA | 0.90(0.65-1.26) | 0.0(0.474) | 1.02(0.64-1.63) | Y | HKSJ | 0.564 | 0.906[0.195, 4.213] | 0 | < 3 studies | < 3 studies | NA | ns | N |
| Wilson 2019 | ATB[-42,0] | Multiple | 6 | 131/658 | PFS | HR | Random | NA | 2.10(1.44-3.06) | 54.4(NA) | 1.50(1.00-2.20) | N | DL | 0.0000698 | 2.11[1.46, 3.048] | 51.152 | [0.753, 5.909] | 0.0795 | 0.000687 | Ⅳ | Y |
| Wu 2021 | ATB (Before ICIs initiation) | Multiple | 13 | 382/1349 | PFS | HR | Random | NA | 1.23(1.14-1.32) | 42(0.05) | 1.12(0.96-1.3) | N | DL | 4.24E-08 | 1.226[1.14, 1.319] | 42.419 | [1.004, 1.497] | 0.0657 | 0.025 | Ⅳ | Y |
| Huang, Xuanzhang 2019 | ATB (Before ICIs initiation) | Multiple | 9 | 322/1099 | PFS | HR | Fixed | NA | 1.70(1.43-2.02) | 30.1(0.178) | 1.37(0.76-2.45) | N | DL | 0.000000487 | 1.73[1.397, 2.142] | 26.561 | [1.083, 2.762] | 0.376 | 0.262 | Ⅳ | Y |
| Chen, Hua 2021 | ATB (any exposure window) | NSCLC | 13 | 430/1211 | OS | HR | Fixed | <0.001 | 2.16(1.79-2.60) | 47.8(0.028) | 2.0(1.30-3.20) | N | DL | 1.32E-09 | 2.338[1.777, 3.077] | 47.93 | [1.049, 5.213] | 0.062 | 0.00052 | Ⅳ | Y |
| Lurienne 2020 | ATB (any exposure window) | NSCLC | 21 | 1248/4311 | OS | HR | Random | NA | 1.69(1.25-2.29) | 80(0.364) | 1.16(0.54-2.49) | N | DL | 0.0000584 | 1.66[1.297, 2.126] | 80.353 | [0.572, 4.817] | 0.00642 | 0.000000001 | Ⅲ | Y |
| Xu 2020 | ATB (any exposure window) | NSCLC | 12 | 531/1576 | OS | HR | Random | 0.0007 | 1.73(1.26-2.38) | 88(<0.0001) | 1.32(1.06-1.64) | N | DL | 0.000668 | 1.733[1.262, 2.379] | 87.914 | [0.568, 5.289] | 0.0225 | 0 | Ⅳ | Y |
| Zhou 2022 | ATB (any exposure window) | NSCLC | 17 | 998/2520 | OS | HR | Random | <0.00001 | 2.09(1.69-2.58) | 77(<0.00001) | 1.32(1.12-1.56) | N | DL | 1.15E-11 | 2.088[1.688, 2.583] | 77.543 | [0.933, 4.676] | 0.0141 | 2.53E-08 | Ⅳ | Y |
| Wu 2021 | ATB (any exposure window) | NSCLC | 9 | 326/950 | ORR | OR | Random | NA | 0.84(0.50-1.42) | 54(0.03) | 0.48(0.19-1.20) | N | DL | 0.515 | 0.84[0.497, 1.42] | 54.196 | [0.183, 3.849] | 0.973 | 0.577 | ns | N |
| Xu 2020 | ATB (any exposure window) | NSCLC | 8 | 241/860 | PFS | HR | Random | 0.0004 | 1.39(1.16-1.67) | 90(<0.0001) | 1.50(1.02-2.20) | N | DL | 0.000378 | 1.392[1.16, 1.67] | 90.132 | [0.783, 2.474] | 0.0474 | 6.55E-08 | Ⅳ | Y |
| Chen, Hua 2021 | ATB (any exposure window) | NSCLC | 13 | 397/1123 | PFS | HR | Random | <0.001 | 1.41(1.23-1.61) | 60.3(0.003) | 1.2(0.90-1.70) | N | DL | 0.00019 | 1.547[1.23, 1.946] | 58.745 | [0.751, 3.186] | 0.0239 | 0.274 | Ⅳ | Y |
| Lurienne 2020 | ATB (any exposure window) | NSCLC | 17 | 660/1547 | PFS | HR | Random | NA | 1.47(1.13-1.90) | 69(0.189) | 1.20(0.90-1.60) | N | DL | 0.000775 | 1.453[1.168, 1.806] | 69.286 | [0.648, 3.258] | 0.208 | 0.00124 | Ⅳ | Y |
| Jiang 2022 | ATB (any exposure window) | NSCLC | 4 | 146/682 | PFS | HR | Random | 0.007 | 1.47(1.11-1.95) | 47(0.13) | 1.25(0.85-1.84) | N | HKSJ | 0.0113 | 1.132[1.031, 1.243] | 78.141 | [0.782, 1.639] | 0.129 | 0.000146 | Ⅳ | Y |
| Zhou 2022 | ATB (any exposure window) | NSCLC | 13 | 586/1509 | PFS | HR | Random | <0.00001 | 1.81(1.47-2.24) | 73(<0.001) | 1.14(0.95-1.38) | N | DL | 2.78E-08 | 1.813[1.47, 2.236] | 73.058 | [0.884, 3.718] | 0.0189 | 0.143 | Ⅳ | Y |
| Huang, Litang2021 | ATB (any exposure window) | Multiple | 15 | 1357/4849 | OS | HR | Random | NA | 1.81(1.43-2.28) | 61.5(0.001) | 1.16(0.54-2.47) | N | DL | 0.000000606 | 1.807[1.432, 2.279] | 61.635 | [0.862, 3.787] | 0.333 | 0.00504 | Ⅲ | Y |
| Huang, Xuanzhang 2019 | ATB (any exposure window) | Multiple | 19 | 667/1787 | OS | HR | Fixed | NA | 2.37(2.05-2.75) | 0.0(0.851) | 1.72(0.94-3.16) | N | DL | 1.17E-30 | 2.365[2.043, 2.739] | 0 | [2.02, 2.77] | 0.233 | 0.403 | Ⅳ | Y |
| Petrelli 2020 | ATB (any exposure window) | Multiple | 14 | 987/1520 | OS | HR | Random | <0.0001 | 2.01(1.48-2.74) | 87(<0.0001) | 2.21(1.70-2.87) | N | DL | 0.0000101 | 2.013[1.475, 2.746] | 87.142 | [0.66, 6.139] | 0.00037 | 3.89E-15 | Ⅳ | Y |
| Tsikala 2021 | ATB (any exposure window) | Multiple | 22 | 1349/5581* | OS | HR | Random | <0.0001 | 1.88(1.59-2.22) | 52(0.002) | 1.16(0.54-2.49) | N | DL | 1.74E-13 | 1.876[1.587, 2.218] | 52.214 | [1.068, 3.296] | 0.0931 | 0.000777 | Ⅲ | Y |
| Wilson 2019 | ATB (any exposure window) | Multiple | 21 | 785/2049 | OS | HR | Random | <0.001 | 1.92(1.37-2.68) | 84.8(NA) | 1.70(1.21-2.38) | N | DL | 1.27e-04 | 1.922[1.376, 2.684] | 84.802 | [0.427, 8.649] | 4.43e-01 | 4.85e-08 | Ⅳ | Y |
| Xu 2020 | ATB (any exposure window) | Multiple | 5 | 191/685 | OS | HR | Random | 0.002 | 2.49(1.38-4.48) | 89(<0.0001) | 1.39(1.21-1.60) | Y | DL | 0.00229 | 2.49[1.385, 4.474] | 88.492 | [0.29, 21.402] | 0.135 | 0.00000058 | Ⅳ | Y |
| Xu 2020 | ATB (any exposure window) | Multiple | 20 | 768/2477 | OS | HR | Random | <0.0001 | 1.90(1.55-2.34) | 89(<0.0001) | 1.32(1.06-1.64) | N | DL | 1.21e-09 | 1.902[1.546, 2.341] | 89.275 | [0.846, 4.28] | 2.14e-04 | 0.00e+00 | Ⅳ | Y |
| Yang 2020 | ATB (any exposure window) | Multiple | 29 | 1250/3034* | OS | HR | Random | <0.0001 | 1.76(1.41-2.19) | 79(<0.0001) | 1.32(1.06-1.64) | N | DL | 0.000000521 | 1.756[1.409, 2.188] | 78.569 | [0.614, 5.018] | 0.0813 | 1.47E-13 | Ⅱ | Y |
| Yu 2021 | ATB (any exposure window) | Multiple | 26 | 2136/4020 | OS | HR | Random | <0.0001 | 1.80(1.44-2.26) | 86(<0.0001) | 1.06(0.93-1.20) | N | DL | 0.0000003 | 1.804[1.439, 2.26] | 85.602 | [0.618, 5.262] | 0.00806 | 5.01E-11 | Ⅲ | Y |
| Jiang 2022 | ATB (any exposure window) | Multiple | 13 | 1409/4570 | OS | HR | Fixed | <0.00001 | 1.46(1.32-1.61) | 37(0.08) | 1.25(1.09-1.43) | N | DL | 1.07E-09 | 1.623[1.389, 1.897] | 36.713 | [1.102, 2.391] | 0.00268 | 0.000686 | Ⅱ | Y |
| Zhou 2022 | ATB (any exposure window) | Multiple | 39 | 2174/6761* | OS | HR | Random | <0.00001 | 1.94(1.68-2.25) | 84(<0.00001) | 1.65(1.29-2.12) | N | DL | 1.78E-18 | 1.943[1.675, 2.254] | 83.939 | [0.844, 4.472] | 0.0000295 | 4.52E-09 | Ⅱ | Y |
| Huang, Litang2021 | ATB (any exposure window) | Multiple | 11 | 517/1109 | PFS | HR | Random | NA | 1.81(1.40-2.34) | 55(0.014) | 1.40(1.03-1.92) | N | DL | 0.00000479 | 1.79[1.395, 2.298] | 55.333 | [0.855, 3.75] | 0.0288 | 0.411 | Ⅳ | Y |
| Huang, Xuanzhang 2019 | ATB (any exposure window) | Multiple | 17 | 486/1705 | PFS | HR | Random | NA | 1.84(1.49-2.26) | 56.2(0.002) | 1.37(0.76-2.45) | N | DL | 2.14E-08 | 1.832[1.482, 2.265] | 55.655 | [0.89, 3.775] | 0.0251 | 0.0326 | Ⅳ | Y |
| Petrelli 2020 | ATB (any exposure window) | Multiple | 13 | 442/1117 | PFS | HR | Random | <0.001 | 1.53(1.22-1.93) | 77(<0.0001) | 1.40(1.03-1.91) | N | DL | 0.000277 | 1.543[1.221, 1.95] | 77.138 | [0.741, 3.215] | 0.00479 | 5.32E-08 | Ⅳ | Y |
| Tsikala 2021 | ATB (any exposure window) | Multiple | 17 | 600/1615 | PFS | HR | Random | <0.0001 | 1.93(1.59-2.36) | 53(0.006) | 1.40(1.03-1.91) | N | DL | 8.07E-11 | 1.935[1.586, 2.361] | 52.867 | [1.012, 3.698] | 0.0485 | 0.0187 | Ⅳ | Y |
| Tsikala 2021 | ATB (any exposure window) | Multiple | 6 | 221/495 | PFS | HR | Random | <0.0001 | 1.62(1.33-1.95) | 52(0.07) | 1.56(1.16-2.09) | N | DL | 0.000482 | 1.706[1.264, 2.302] | 51.616 | [0.736, 3.954] | 0.331 | 0.406 | Ⅳ | Y |
| Wilson 2019 | ATB (any exposure window) | Multiple | 16 | 575/1604 | PFS | HR | Random | <0.0001 | 1.65(1.30-2.10) | 73.6(NA) | 1.56(1.16-2.09) | N | DL | 9.66e-05 | 1.655[1.285, 2.132] | 76.271 | [0.635, 4.316] | 1.06e-01 | 5.44e-13 | Ⅳ | Y |
| Yang 2020 | ATB (any exposure window) | Multiple | 29 | 1204/2970 | PFS | HR | Random | <0.0001 | 1.76(1.47-2.12) | 72(<0.0001) | 1.17(0.97-1.41) | N | DL | 1.53E-09 | 1.763[1.467, 2.12] | 72.344 | [0.763, 4.074] | 0.00282 | 0 | Ⅲ | Y |
| Yu 2021 | ATB (any exposure window) | Multiple | 23 | 1688/2998 | PFS | HR | Random | <0.0001 | 1.55(1.26-1.91) | 79(<0.0001) | 0.94(0.83-1.07) | N | DL | 0.000041 | 1.55[1.257, 1.91] | 79.378 | [0.625, 3.844] | 0.00103 | 2.83E-09 | Ⅲ | Y |
| Jiang 2022 | ATB (any exposure window) | Multiple | 14 | 1414/4596 | PFS | HR | Random | <0.00001 | 1.60(1.33-1.92) | 68(0.0001) | 1.16(1.04-1.29) | N | DL | 0.000000431 | 1.601[1.334, 1.921] | 68.247 | [0.879, 2.915] | 0.000454 | 1.09E-08 | Ⅱ | Y |
| Zhou 2022 | ATB (any exposure window) | Multiple | 33 | 1521/3099* | PFS | HR | Random | <0.00001 | 1.83(1.53-2.19) | 86(<0.00001) | 1.14(0.95-1.38) | N | DL | 4.88E-11 | 1.828[1.527, 2.188] | 85.859 | [0.695, 4.806] | 0.0000272 | 8.53E-08 | Ⅲ | Y |
| Xu 2020 | ATB (any exposure window) | Multiple | 3 | 105/376 | PFS | HR | Random | 0.0002 | 1.23(1.10-1.37) | 0(0.71) | 1.22(1.09-1.37) | N | DL | 0.0234 | 1.229[1.071, 1.411] | 0 | [0.818, 1.847] | 0.525 | 0.593 | Ⅳ | Y |
| Xu 2020 | ATB (any exposure window) | Multiple | 14 | 392/1452 | PFS | HR | Random | <0.0001 | 1.53(1.30-1.79) | 90(<0.0001) | 1.22(1.09-1.37) | N | DL | 0.00168 | 1.531[1.212, 1.933] | 90.158 | [0.852, 2.75] | 0.00356 | 1.99E-11 | Ⅳ | Y |
| Xu 2020 | ATB (any exposure window) | Multiple | 8 | 234/843 | ORR | OR | Fixed | <0.0001 | 2.28(1.56-3.33) | 32(0.17) | 2.21(1.01-4.85) | N | DL | 0.00276 | 0.498[0.315, 0.786] | 22.188 | [0.194, 1.278] | 0.264 | 0.278 | Ⅳ | Y |
| Yu 2021 | ATB (any exposure window) | Multiple | 16 | 1353/2306 | ORR | OR | Random | 0.03 | 0.63(0.42-0.95) | 63(0.0004) | 0.94(0.75-1.19) | N | DL | 0.0374 | 0.653[0.437, 0.975] | 60.129 | [0.177, 2.404] | 0.142 | 0.103 | Ⅳ | Y |
| Zhou 2022 | ATB (any exposure window) | Melanoma | 4 | 270/2153 | OS | HR | Random | <0.0001 | 1.94(1.41-2.67) | 55(0.08) | 1.65(1.29-2.12) | N | HKSJ | 0.054 | 1.943[0.979, 3.857] | 54.696 | [0.51, 7.407] | 0.168 | 0.159 | ns | Y |
| Jiang 2022 | ATB (any exposure window) | Melanoma | 2 | 40/203 | PFS | HR | Random | 0.19 | 1.95(0.73-5.25) | 71(0.06) | 1.28(0.8-2.04) | N | HKSJ | 0.412 | 1.951[0.003, 1198.368] | 71.489 | < 3 studies | < 3 studies | NA | ns | Y |
| Xu 2020 | ATB (any exposure window) | RCC | 2 | 36/152 | OS | HR | Random | 0.15 | 1.84(0.81-4.16) | 58(0.12) | 3.50(1.10-11.14) | N | HKSJ | 0.384 | 1.835[0.009, 370.066] | 57.799 | < 3 studies | < 3 studies | NA | ns | N |
| Luo 2022 | ATB (any exposure window) | RCC | 5 | 237/836 | OS | HR | Fixed | NA | 1.69(1.34-2.12) | 25(0.25) | 1.59(1.22-2.09) | N | DL | 0.000961 | 1.756[1.257, 2.452] | 25.579 | [0.765, 4.027] | 0.734 | 0.0292 | Ⅳ | Y |
| Zhou 2022 | ATB (any exposure window) | RCC | 2 | 62/177 | OS | HR | Random | 0.01 | 1.81(1.14-2.87) | 39(0.20) | 1.44(0.88-2.37) | N | HKSJ | 0.24 | 1.809[0.091, 35.841] | 38.5 | < 3 studies | < 3 studies | NA | ns | N |
| Tsikala 2021 | ATB (any exposure window) | RCC | 3 | 76/167 | PFS | HR | Random | <0.0001 | 2.42(1.60-3.68) | 0(0.77) | 3.10(1.40-6.86) | Y | HKSJ | 0.0159 | 2.378[1.479, 3.825] | 0 | [0.584, 9.678] | 0.523 | 0.487 | Ⅳ | Y |
| Xu 2020 | ATB (any exposure window) | RCC | 2 | 36/152 | PFS | HR | Random | 0.0002 | 2.47(1.52-3.99) | 0(0.48) | 3.10(1.40-6.86) | Y | HKSJ | 0.121 | 2.465[0.27, 22.515] | 0 | < 3 studies | < 3 studies | NA | ns | N |
| Jiang 2022 | ATB (any exposure window) | RCC | 4 | 761/2972 | PFS | HR | Random | 0.01 | 2.26(1.17-4.36) | 84(0.0003) | 1.16(1.04-1.29) | Y | HKSJ | 0.105 | 2.261[0.732, 6.982] | 84.197 | [0.12, 42.669] | 0.0142 | 1.58E-09 | ns | N |
| Luo 2022 | ATB (any exposure window) | RCC | 6 | 242/862 | PFS | HR | Random | NA | 1.77(1.25-2.50) | 56(0.04) | 1.24(0.99-1.55) | N | DL | 0.00194 | 1.784[1.237, 2.572] | 56.217 | [0.64, 4.973] | 0.155 | 0.000514 | Ⅳ | Y |
| Zhou 2022 | ATB (any exposure window) | RCC | 4 | 92/208 | PFS | HR | Random | <0.00001 | 3.14(2.16-4.58) | 37(0.19) | 3.1(1.69-5.68) | Y | HKSJ | 0.00978 | 3.144[1.693, 5.841] | 37.011 | [0.855, 11.569] | 0.0348 | 0.173 | Ⅳ | Y |
| Luo 2022 | ATB (any exposure window) | RCC | 3 | 164/770 | ORR | OR | Fixed | NA | 0.58(0.41-0.84) | 0(0.37) | 0.73(0.45-1.18) | N | HKSJ | 0.106 | 0.564[0.236, 1.348] | 6.305 | [0.236, 1.348] | 0.0931 | 0.0274 | ns | N |
| Petrelli 2020 | Steroids | Multiple | 15 | 802/3442* | OS | HR | Random | 0.0001 | 1.54(1.24-1.91) | 64(0.0003) | 1.46(1.16-1.84) | N | DL | 0.000113 | 1.538[1.236, 1.914] | 64.198 | [0.744, 3.181] | 0.413 | 0.00962 | Ⅳ | Y |
| Petrelli 2020 | Steroids | Multiple | 10 | 585/3259 | PFS | HR | Random | 0.03 | 1.34(1.02-1.76) | 75(<0.0001) | 1.31(1.07-1.60) | N | DL | 0.0338 | 1.341[1.023, 1.759] | 75.339 | [0.563, 3.196] | 0.725 | 0.00000037 | Ⅳ | Y |
| Zhang, Yongchao 2021 | NSAIDs | Multiple | 7 | 374/1675 | OS | HR | Fixed | 0.769 | 0.98(0.83-1.15) | 48.45(0.07) | 1.3(0.92-1.83) | Y | DL | 0.386 | 0.894[0.694, 1.152] | 48.538 | [0.459, 1.741] | 0.0372 | 0.802 | ns | Y |
| Zhang, Yongchao 2021 | NSAIDs | Multiple | 5 | 322/1600 | PFS | HR | Fixed | 0.213 | 0.90(0.77-1.06) | 22.12(0.27) | 1.07(0.78-1.47) | N | DL | 0.258 | 0.897[0.743, 1.083] | 21.38 | [0.578, 1.392] | 0.49 | 0.247 | ns | Y |
| Zhang, Yongchao 2021 | Beta blockers | Multiple | 4 | 207/1244* | OS | HR | Fixed | 0.207 | 0.87(0.71-1.08) | 0(0.63) | 0.90(0.68-1.20) | Y | HKSJ | 0.196 | 0.875[0.677, 1.131] | 0 | [0.677, 1.131] | 0.851 | 0.735 | ns | Y |
| Kennedy 2022 | Beta blockers | Multiple | 8 | 1265/4124* | OS | HR | NA | NA | 0.99(0.83-1.18) | NA | 1.05(0.91-1.21) | N | DL | 0.86 | 0.984[0.82, 1.18] | 57.734 | [0.6, 1.613] | 0.113 | 0.162 | ns | Y |
| Zhang, Yongchao 2021 | Beta blockers | Multiple | 4 | 207/1244* | PFS | HR | Random | 0.486 | 0.91(0.66-1.26) | 53.64(0.09) | 0.95(0.74-1.21) | Y | HKSJ | 0.694 | 0.915[0.479, 1.751] | 53.38 | [0.479, 1.751] | 0.987 | 0.8 | ns | Y |
| Kennedy 2022 | Beta blockers | Multiple | 7 | 961/4757 | PFS | HR | NA | NA | 0.97(0.89-1.05) | NA | 1.01(0.90-1.13) | Y | DL | 0.391 | 0.961[0.876, 1.053] | 4.562 | [0.833, 1.108] | 0.00533 | 0.732 | ns | Y |
| Zhang, Lilong 2022 | Probiotics | NSCLC | 3 | 77/559 | OS | HR | Random | NA | 0.53(0.31-0.91) | 41.5(0.181) | 0.61(0.48-0.76) | N | HKSJ | 0.153 | 0.527[0.155, 1.791] | 41.275 | [0.155, 1.791] | 0.763 | 0.0465 | ns | N |
| Zhang, Lilong 2022 | Probiotics | NSCLC | 3 | 77/559 | PFS | HR | Random | NA | 0.53(0.35-0.80) | 46.9(0.152) | 0.50(0.41-0.61) | N | HKSJ | 0.127 | 0.531[0.181, 1.558] | 47.761 | [0.181, 1.558] | 0.654 | 0.438 | ns | N |
| Mao 2022 | Opioids | Multiple | 6 | 490/1565 | OS | HR | Random | NA | 1.67(1.30-2.14) | 69(0.007) | 1.53(1.11-2.11) | Y | DL | 0.0000537 | 1.672[1.303, 2.145] | 68.233 | [0.764, 3.657] | 0.00323 | 0.355 | Ⅳ | Y |
| Mao 2022 | Opioids | Multiple | 4 | 203/1180 | PFS | HR | Fixed | NA | 1.61(1.37-1.89) | 0(0.470) | 0.76(0.43-1.33) | Y | HKSJ | 0.0083 | 1.606[1.261, 2.044] | 0 | [1.261, 2.044] | 0.126 | 0.00402 | Ⅳ | Y |

^†^ Whether the statistical significance was consistent with the results of included meta-analyses.

^*^ Review reported incomplete data on sample size.

**Abbreviation:** ATB, antibiotics; CM, concomitant medications; CI, confidence interval; DL, DerSimonian-Laird method; ES, effect size; HR, hazard ratio; HKSJ, Hartung-Knapp-Sidik-Jonkman method; MA, meta-analysis; NA, not available; N, No; NSAIDs, nonsteroidal anti-inflammatory agents; NSCLC, non-small cell lung cancer; OS, overall survival; ORR, objective response rate; OR, odds ratio; PI, prediction interval; PFS, progression-free survival; PPIs, proton pump inhibitors; RA, re-analysis; RCC, renal cell carcinoma; TES, test of excess significance; UC, urothelial carcinoma; Y, Yes; Ⅰ, convincing evidence (class Ⅰ); Ⅱ, highly suggestive evidence (class Ⅱ); Ⅲ, suggestive evidence (class Ⅲ); Ⅳ, weak evidence (class Ⅳ); ns, non-significant (class ns).
