# Supplementary figures and images for: Impact of concomitant medications on the efficacy of immune checkpoint inhibitors: an umbrella review
Source: Front Immunol. 2023 Sep 29;14:1218386. doi: 10.3389/fimmu.2023.1218386 (PMC10570520; doi:10.3389/fimmu.2023.1218386)

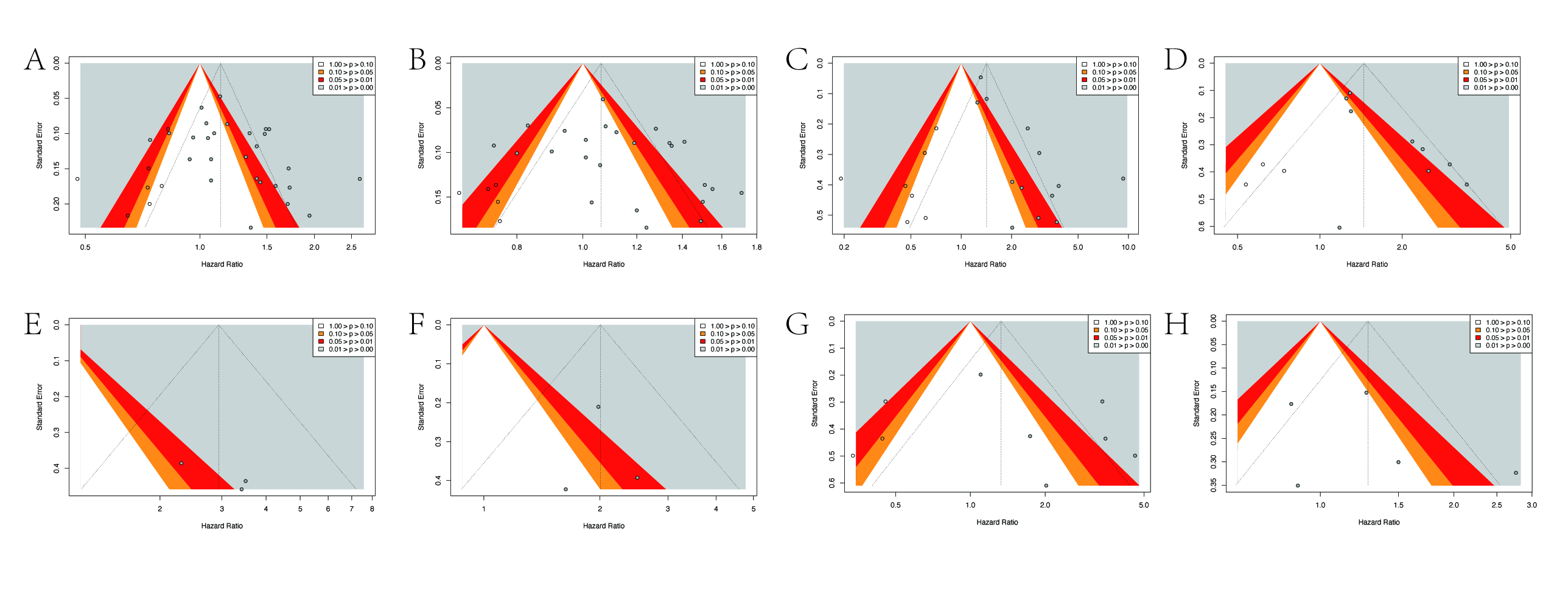

Supplement: Supplementary file 1 [file DataSheet_1.zip › Supplementary_Materials/Fig S6.tif]

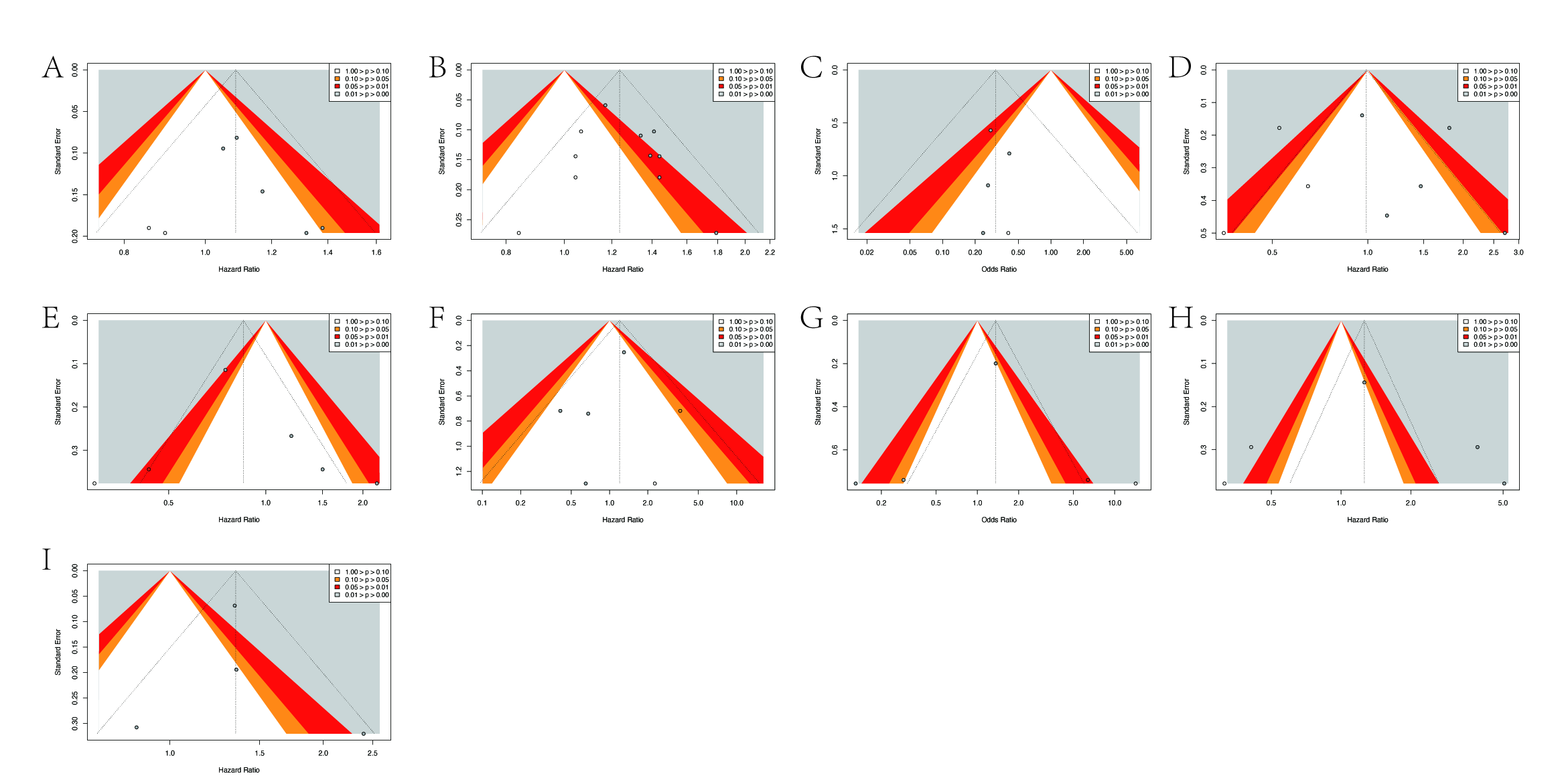

Supplement: Supplementary file 1 [file DataSheet_1.zip › Supplementary_Materials/Fig S7.tif]

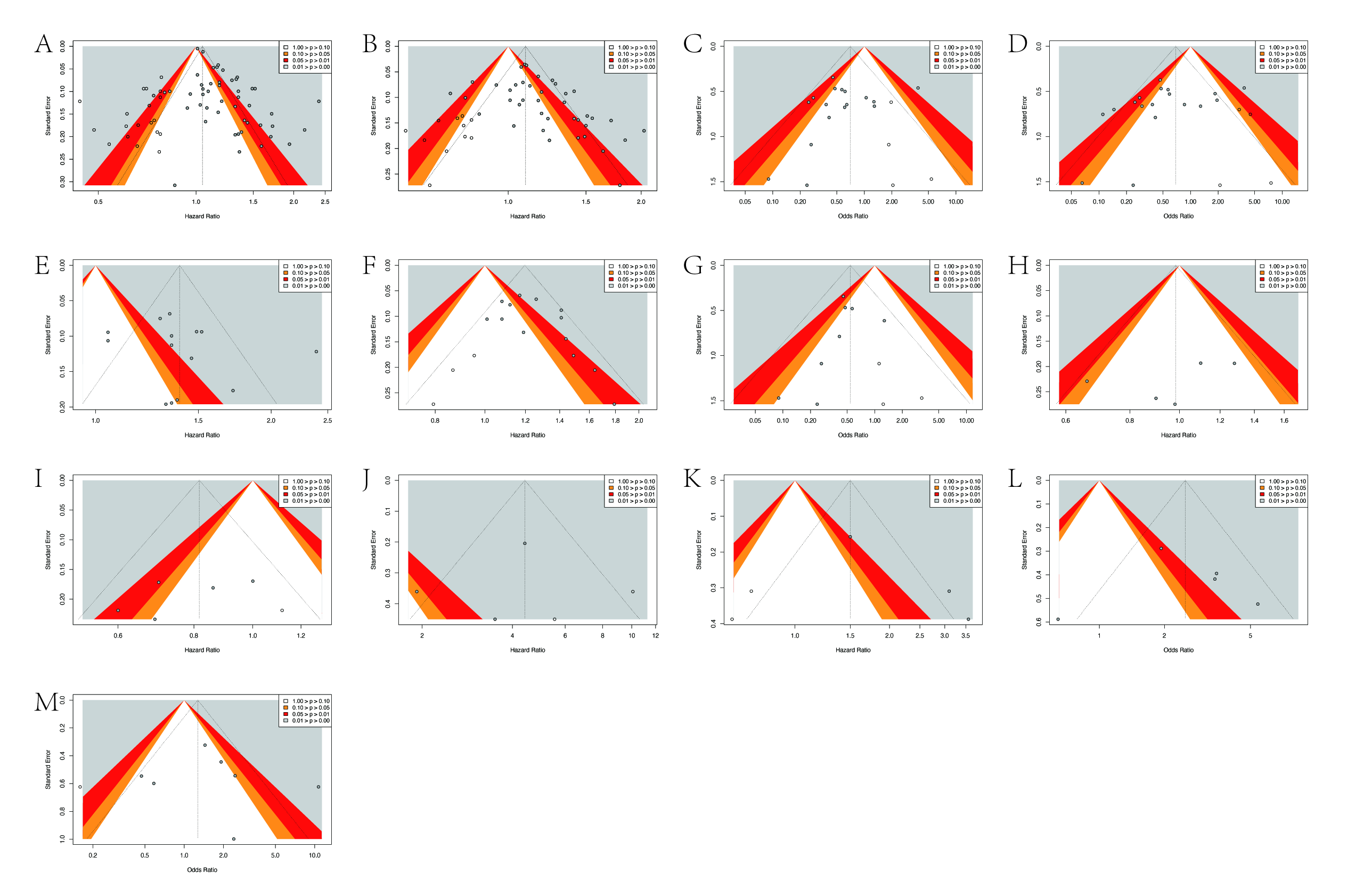

Supplement: Supplementary file 1 [file DataSheet_1.zip › Supplementary_Materials/Fig S5.tif]

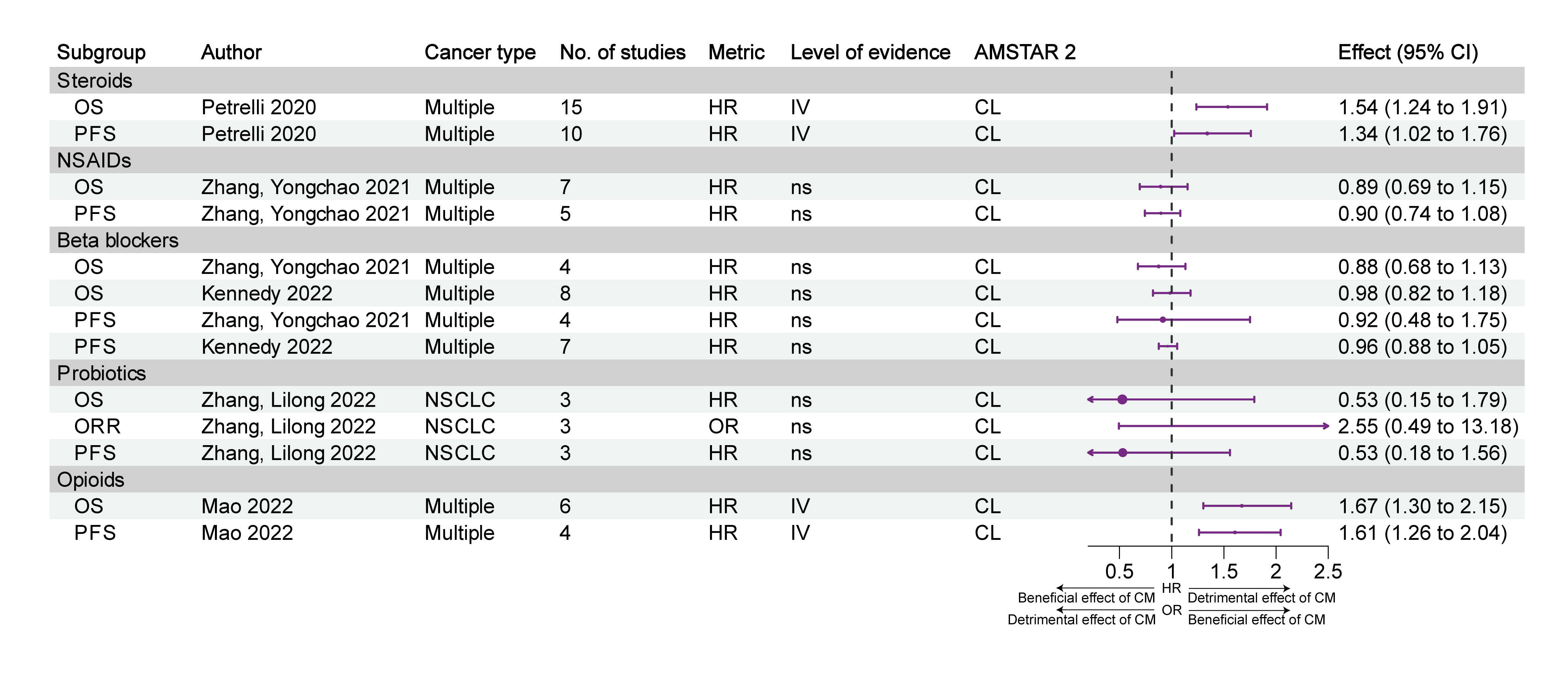

Supplement: Supplementary file 1 [file DataSheet_1.zip › Supplementary_Materials/Fig S4.tif]

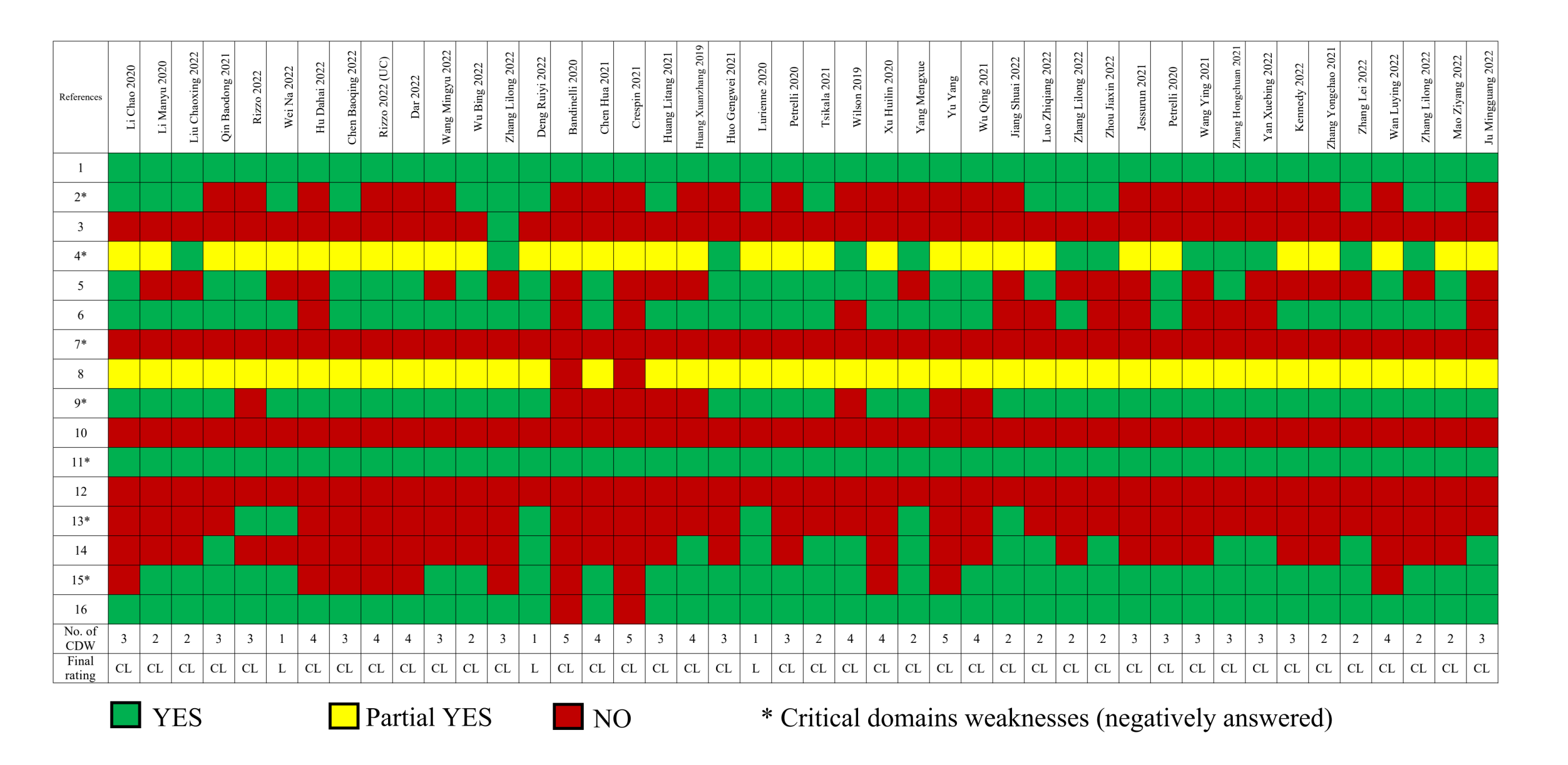

Supplement: Supplementary file 1 [file DataSheet_1.zip › Supplementary_Materials/Fig S1.tif]

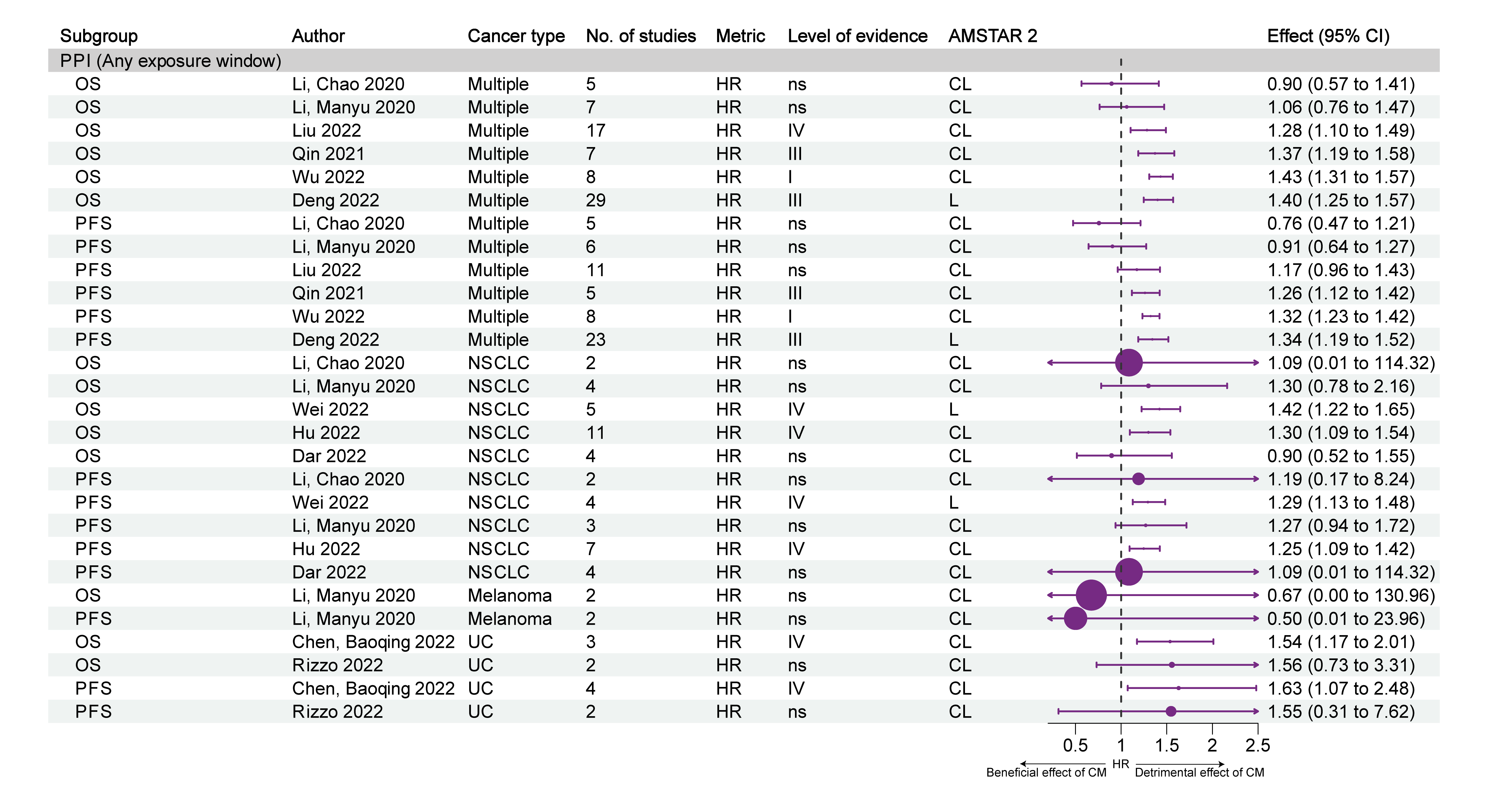

Supplement: Supplementary file 1 [file DataSheet_1.zip › Supplementary_Materials/Fig S3.tif]

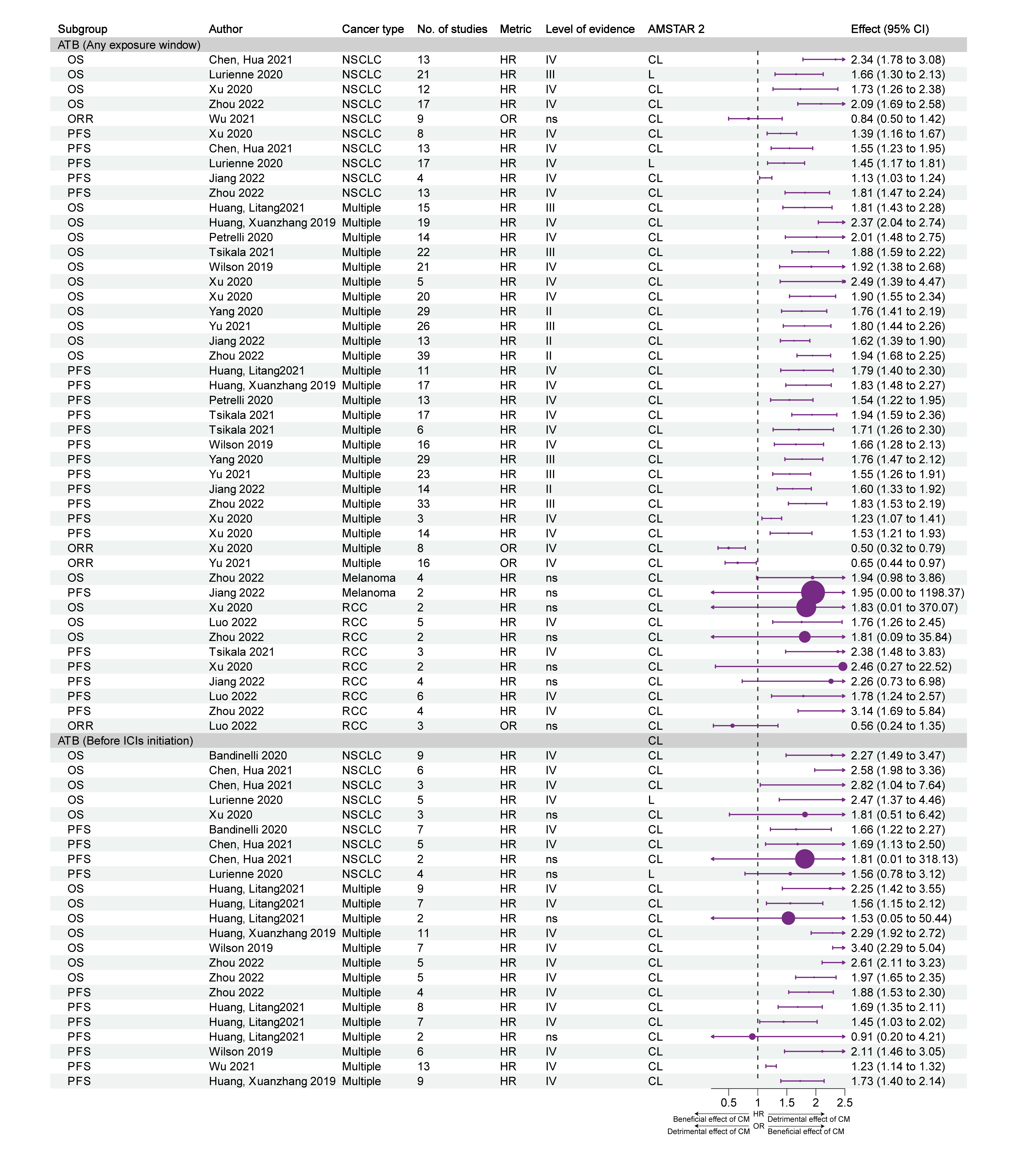

Supplement: Supplementary file 1 [file DataSheet_1.zip › Supplementary_Materials/Fig S2.tif]

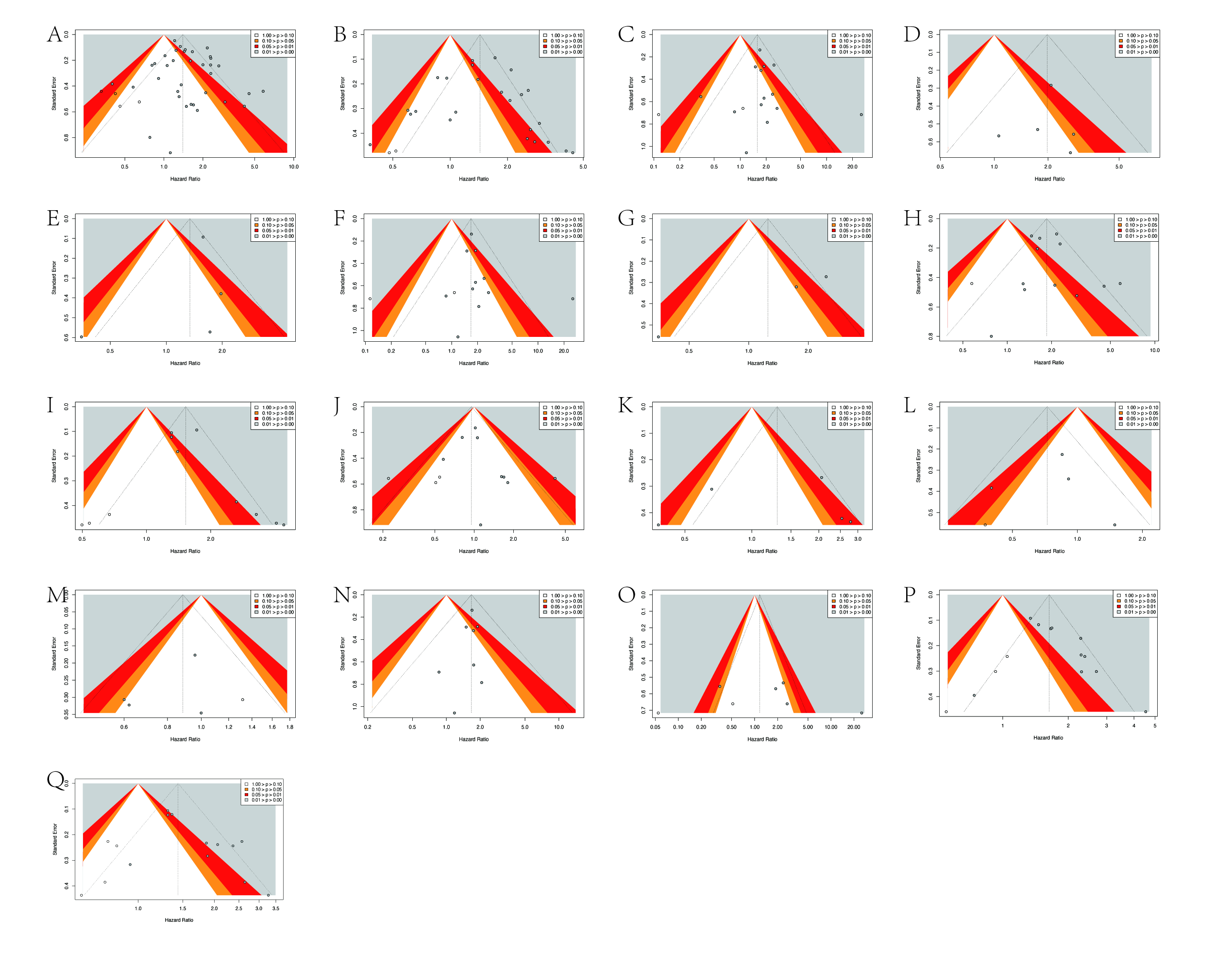

Supplement: Supplementary file 1 [file DataSheet_1.zip › Supplementary_Materials/Fig S9.tif]

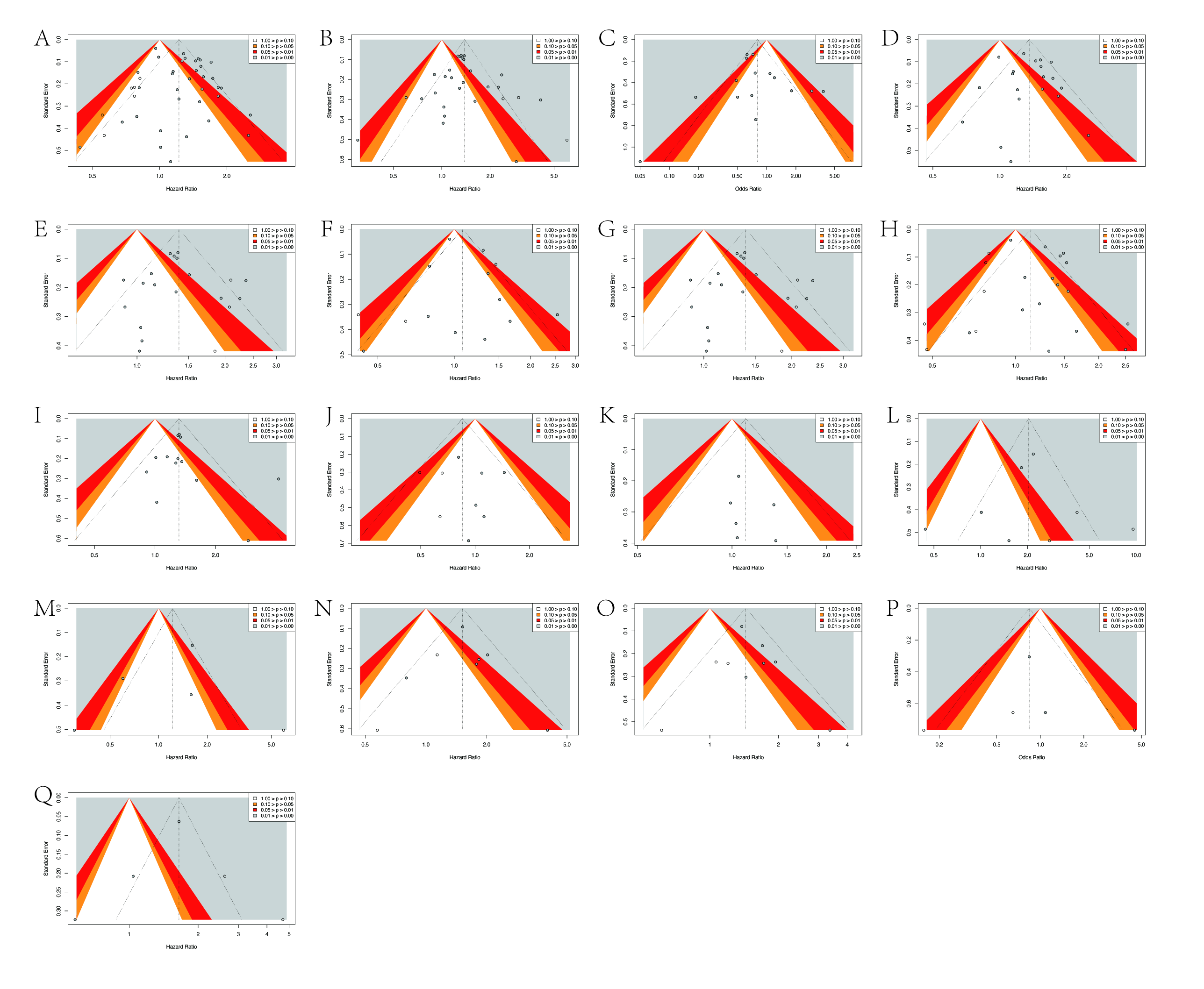

Supplement: Supplementary file 1 [file DataSheet_1.zip › Supplementary_Materials/Fig S8.tif]

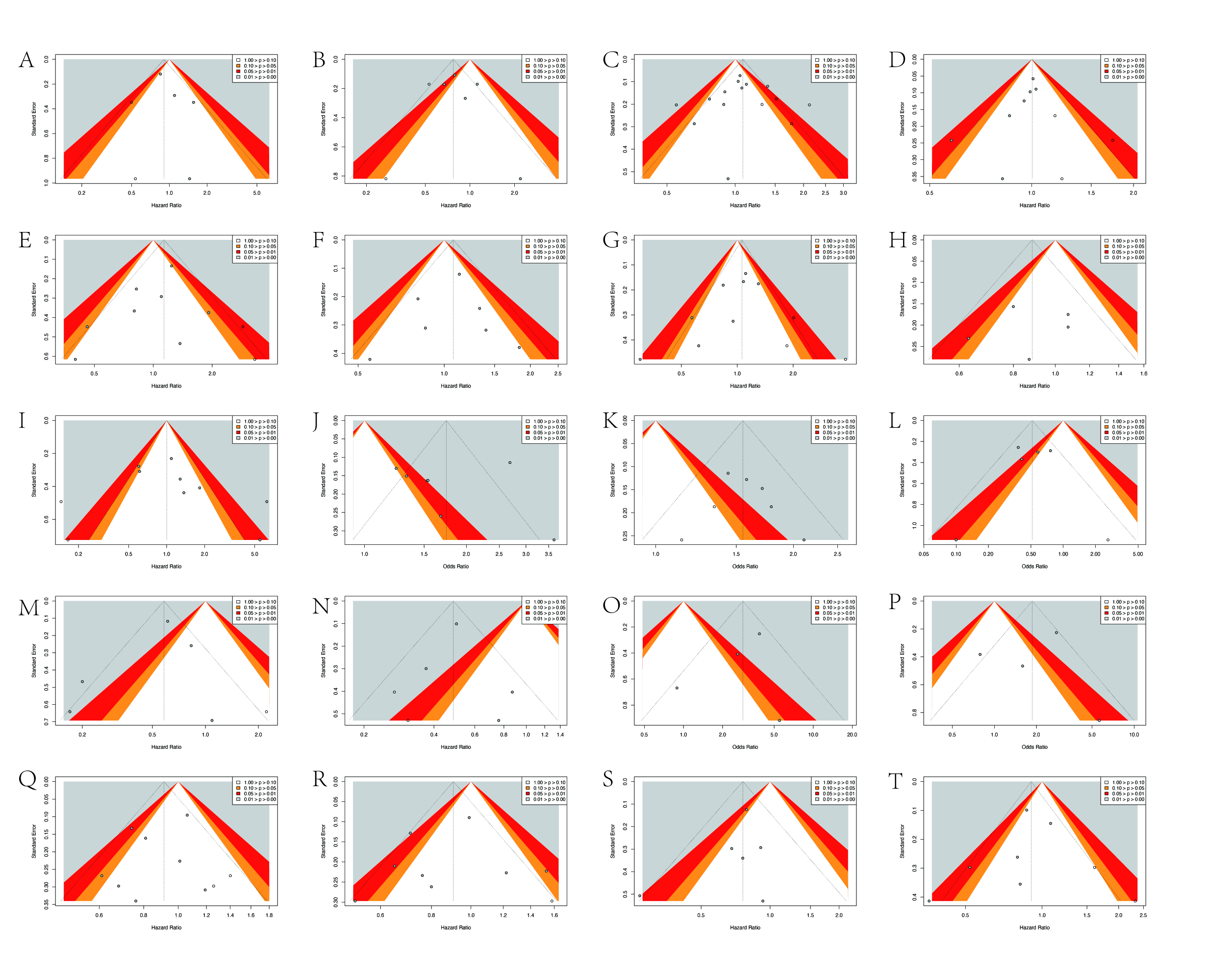

Supplement: Supplementary file 1 [file DataSheet_1.zip › Supplementary_Materials/Fig S10.tif]
